# Reprever: resolving low-copy duplicated sequences using template driven assembly

**DOI:** 10.1093/nar/gkt339

**Published:** 2013-05-08

**Authors:** Sangwoo Kim, Paul Medvedev, Tara A. Paton, Vineet Bafna

**Affiliations:** ^1^Department of Computer Science and Engineering, 9500 Gilman Dr University of California at San Diego, La Jolla, CA 92122, USA and ^2^Genetic Analysis Facility, The Centre for Applied Genomics, The Hospital for Sick Children, 101 College Street, Toronto, ON M5G 1L7, Canada

## Abstract

Genomic sequence duplication is an important mechanism for genome evolution, often resulting in large sequence variations with implications for disease progression. Although paired-end sequencing technologies are commonly used for structural variation discovery, the discovery of novel duplicated sequences remains an unmet challenge. We analyze duplicons starting from identified high-copy number variants. Given paired-end mapped reads, and a candidate high-copy region, our tool, Reprever, identifies (a) the insertion breakpoints where the extra duplicons inserted into the donor genome and (b) the actual sequence of the duplicon. Reprever resolves ambiguous mapping signatures from existing homologs, repetitive elements and sequencing errors to identify breakpoint. At each breakpoint, Reprever reconstructs the inserted sequence using profile hidden Markov model (PHMM)-based guided assembly. In a test on 1000 artificial genomes with simulated duplication, Reprever could identify novel duplicates up to 97% of genomes within 3 bp positional and 1% sequence errors. Validation on 680 fosmid sequences identified and reconstructed eight duplicated sequences with high accuracy. We applied Reprever to reanalyzing a re-sequenced data set from the African individual NA18507 to identify >800 novel duplicates, including insertions in genes and insertions with additional variation. polymerase chain reaction followed by capillary sequencing validated both the insertion locations of the strongest predictions and their predicted sequence.

## INTRODUCTION

Copy number variation (CNV) is a form of structural variation that alters the number of copies of DNA segments and includes events like deletion and duplication. The advent of paired-end sequencing technologies enabled such CNVs to be identified in a near-nucleotide level. So far, a many algorithms including discordant paired-end mapping (PEM) (1–4), read-depth methods (5,6), split-read (7–9) and *de novo* sequence assembly of donor genomes (10–12) have been applied to detect and characterize CNVs.

We focus here on the problem of copy number expansions. The regions with higher copy numbers are duplicons of a reference genome segment that was re-inserted into the donor genome at a different location. The characterization of these sequences involves identifying the location of the inserted breakpoint and the sequence of the duplicated insertion. Insertion breakpoint identification has been addressed earlier (13–15), as also reconstruction of inserted regions using *de novo* assembly techniques. NovelSeq (15) was the first study that provided both solutions in identifying novel (non-reference) sequence insertion. This has been reattempted by Parrish *et al.*. (16), with accuracy of 90–92%. Despite this extensive work on CNVs, the problem of breakpoint location and reconstruction of duplicated sequence (high CNV regions) has not been addressed satisfactorily. As Supplementary Table S1 shows, among all of the insertions, and breakpoints reported by multiple groups on the Yoruban individual NA18507, there is not a single reconstruction targeted on duplicated sequences.

This lack of data suggests that the tools for reconstruction of duplicated sequence are lacking. Reconstruction is phenotypically important, as multiple duplications of mutated genes might also lead to an expansion of mutant proteins [e.g. mutations in *ERBB2* (17)]. Unlike deletion or novel sequence insertion events, however, read mapping in these regions is innately confounded. Multiple copies in reference and donor, repeat elements and segmental duplications frequently obscure mapped read counts. Some duplicates exist as a truncated form of the original copy (18,19) causing uneven depth of coverage. We address these issues explicitly.

**Figure 1.**
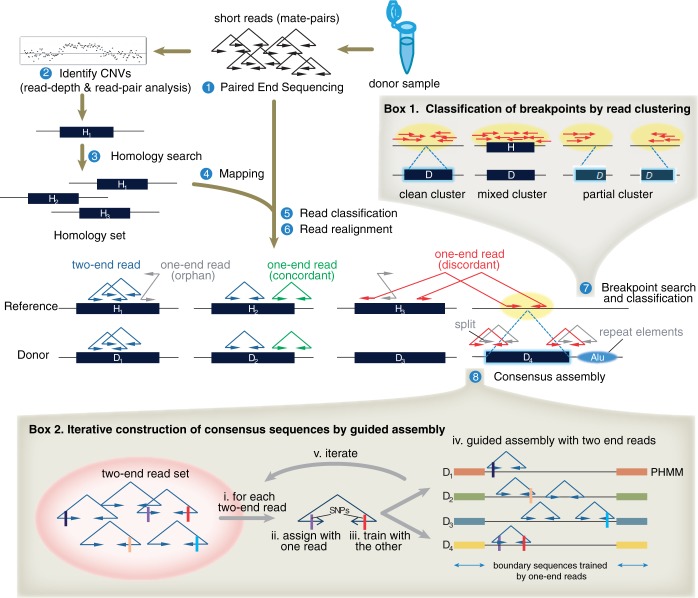
The overall pipeline of Reprever. Insertion breakpoints and consensus sequences of copy count increased regions are assessed through eight steps from donor sample preparation. (1) Donor genome is paired-end sequenced to generate mate pairs. (2) Copy count increased region *H* is predicted from conventional CNV prediction methods, such as read-depth analysis or comparative hybridization. (3 and 4) Search homologs of each copy count increased region *H* in the reference. As read count and mapping is confounded because of the non-unique sequences, we merge and repartition the mate pairs mapped to the homolog set **H** = 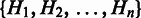. (5) Each mate pair is further classified into five classes by its mapping information (Supplementary Figure S5). (6) Reads with ambiguous or orphan mapping are realigned to be rescued. (7) Insertion locations of extra copies are analyzed by Reprever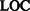 tools. Based on discordant read signatures (red mate pairs), putative breakpoints (yellow ovals) are inferred and interrogated by three independent tests: breakpoint shape (Box 1), breakpoint specificity and breakpoint coverage (data not shown. See ‘Materials and Methods’ section). (8) Once insertion locations are finalized, Reprever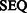 tools reconstruct the sequence at the breakpoint, as well as all the existing homologs using profile hidden Markov model (PHMM) (Box 2). The PHMMs are trained from boundary to center, as mate-pairs are iteratively assigned to train model parameters.

We analyze target CNV regions with a low copy increase in donor using a pipeline called Reprever (repeat resolver). The final goal of Reprever is first, to detect breakpoints of the extra copies in donor, and second, to reconstruct the inserted sequence at the discovered breakpoints. Reprever includes a number of tools that are mainly clustered to Reprever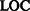 and Reprever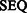 to achieve the goals, respectively (Figure 1). We start this, given a list of candidate regions with increase in copy number (*H*) that can be obtained from any of the conventional CNV identification tools, such as CNVer (4). For each *H*, Reprever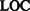 finds the breakpoint considering the homologous regions in the reference. All the paired-end reads of different types (e.g. concordant, discordant or orphan) around the breakpoints are retrieved and saved for the next step (Figure 1, step 1–7 and box 1). Reprever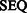, given the list of breakpoints and paired-end reads, reconstructs the inserted sequences (as well as *H*’s homologous sequences) using profile hidden Markov model (PHMM). We explain the details with other optimization techniques in ‘Materials and Methods’ section.

Reprever mainly targets low-copy non-tandem duplicons. This covers most duplicons generated by homologous recombination mechanism. However, there are other mechanisms, including tandem duplications, and also other rearrangements, such as breakage fusion bridge, chromothripsis and retrotransposon-mediated expansions. These have been investigated by other specialized tools (20–22). A series of rigorous tests, including simulated genomes, completed fosmid sequences and polymerase chain reaction (PCR)/capillary sequencing, confirmed the accuracy of Reprever (see ‘Results’ section). Using Reprever, we could also identify many novel duplicates in the first time. Thus, our tool directly addresses the problem of repeat identification, which is one of the most challenging and unexplored parts of SV analysis and should help uncover the functional role of CNV expansions.

## MATERIALS AND METHODS

To identify predicted duplicons, we present two pipelines built around the tools Reprever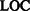 and Reprever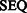, which are applied in order. We will first describe how these two pipelines work in conjunction with detailed methods, then other procedures for data preparation and processing.

### Pipeline for Reprever
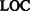


Reprever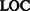 takes two kinds of data for its input; (i) paired-end mapping data, along with the reference genome, and (ii) a candidate list of copy count increased regions. After execution, it outputs (i) multiple insertion breakpoints, one for each duplicon, (ii) confidence score for every breakpoint and (iii) paired-end reads assigned to the input region or any related regions. Reprever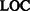 consists of seven steps as shown in Figure 1 and Box 1.

#### Preparing input for Reprever
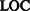


Paired-end mapping data of donor sample (NA18507 and NA12878) are prepared as described earlier in the text. We tested Reprever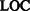 on two types of Illumina Paired-end (100 × 2, 500-bp insert, 40× coverage and 150 × 2, 350-bp insert, 20× coverage) reads. Although Reprever can be applied to paired-end data of any quality, we assume that too low coverage or small insert size may compromise overall performance. As previously described, three candidate CNV region sets are also prepared.

#### Homolog recruitment

The first step in the pipeline implementing Reprever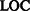 is a recruitment of homologs. Define a homolog of *H* as a genomic region *H_i_* such that (i) the length difference 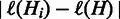 are bounded and (ii) the sequences 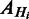 and *A_H_* (in the meaning of answer sequence of *H*) are similar. To recruit homologs, we wrote a script using Blat to query *A_H_* against the reference genome. Regions with >95% sequence similarity and <20% block size were recruited as homologs. The output of recruitment is a homology set H = 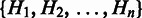, where *H*_1_ is the original candidate region and 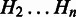 are its homologs.

#### Re-mapping sequencing data

Starting with a homology set H, we searched all mate pairs that have at least one read mapping within 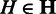 from the whole-genome mapping file (.bam). Multiple mapping information was not included in the whole-genome mapping data, but Phred-scaled mapping quality [‘MAPQ’ in SAM format (23)], read sequence and read quality (‘QUAL’) were preserved for secondary blat search used in breakpoint specificity analysis.

#### Read classification

For a homology set H, we classify the mate pairs mapping to H as follows (see also Supplementary Figure S5):

- Two-end reads have both reads mapping completely within *H*.
- One-end reads have one read mapping within (internal) and the other mapping outside of *H* (external).
- Concordant reads have the two reads mapping to distances that are within the acceptable variation of insert size. Thus, for 500-bp insert reads, we can accept all reads with end points within 300–800 as being concordant and discordant otherwise. A concordant-one-end (respectively, discordant-one-end) read has one end mapping within *H* and the other read mapping concordantly (respectively, discordantly) outside *H*.
- Orphan-one-end reads have one read unmapped.
- Orphan reads are completely unmapped to any portion of the genome.

#### Read realignment

An orphan-one-end read has one mapped and one unmapped read. In this step, the unmapped read is re-analyzed to classify the reasons and rescue if possible. There are many ways a read generated from donor cannot be mapped to the reference (Supplementary Figure S4); excessive variation in the read (>5-bp SNPs and micro insertion/deletion), split over a breakpoint and insertion boundary, repeat elements (e.g. Short Interspersed Nuclear Elements (SINEs), Long Interspersed Nuclear Elements (LINEs), simple tandem repeats) and/or bad sequencing quality cause a mapping loss.

Starting with the set of orphan-one-end reads with one end mapping to candidate region *H*, we first filtered out the reads in which ≥10 nt had <Q20 quality score in Phred-scale. Next, we used a Smith–Waterman alignment to rescue reads that could be aligned to regions in ±1-kb region of the homology set **H**. The read was alignable if (i) the sequence similarity >92 and (ii) alignment score >80% of one in a perfect match. Based on the newly aligned position, the rescued orphan-one-end read was classified as one of the following: two-end read (aligned to *H*); concordant-one-end read (aligned to flanking regions of *H*) and discordant-one-end read (aligned to flanking regions of **H**). For non-alignable orphan sequences, we used a command line version of RepeatMasker (ver 3.3.0) to find out the portion and the kind of included repeat elements.

### Scoring breakpoints

The location of an insertion breakpoint can be inferred from a cluster of external ends of discordant-one-end reads (Supplementary Figures S3 and S5C). We define a cluster of *H* as a set of reads discordantly mapped to 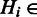 **H** that does not contain an empty region of length *H* in between and is distal from all other reads at least length of *H*. Initially, Reprever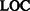 takes all clusters into account and then calculates confidence scores for each cluster using two features. These are breakpoint specificity and breakpoint composition.

#### Breakpoint specificity

Discordant one-end reads are the primary source of breakpoint finding. However, not every discordant one-end read conveys the same amount of information. Although reads that are aligned uniquely in the genome can be used as a critical evidence of insertion breakpoint, some reads that can be aligned to multiple regions merely indicate one possible mapping out of many.

Define a discordant one-end read **r** = (*r*_1_, *r*_2_) and is initially aligned to (*q*_1_, *q*_2_) where *q_i_* is the mapped genomic position of read *r_i_*. We will first identify how specific the mapped positions are. We define a match 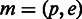 of a read as a pair of mapped position *p* and its edit distance *e* in the alignment. We query each read *r_i_* against reference genome using Blat to retrieve a match set:
(1)
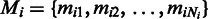

(2)
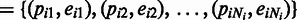

where *N_i_* is the total number of possible mapping positions of *r_i_*, *p_ik_* is the *k*th (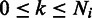) mapped position of *r_i_* and *e_ik_* is the edit distance in the *k*th match. Thus, the match sets of read *r*_1_, *r*_2_ of **r** are defined as
(3)
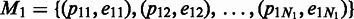

(4)
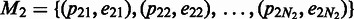

Now we define, a paired match set **M** as a Cartesian product of *M*_1_ and *M*_2_ (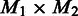). Naturally, each paired match 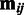 is defined as a pair of two matches (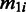, 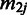) (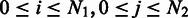). The edit distance of paired match 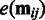 can be calculated as the sum of two edit distances (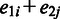).

Depending on the relative positions of two matches, a paired match can be either concordant or discordant. We define a best paired match 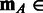 **M** as the paired match with lowest edit distance. Similarly, we define a best concordant paired match 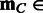 **M** as the concordant paired match with lowest edit distance. Now, for all discordant paired matches, we select ones whose edit distance is lower than 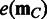. In other words, we always prefer best known concordant paired match to any discordant paired matches unless they represent better alignment. Thus, a preferred discordant paired match set **D** is defined as
(5)
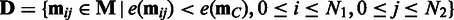

For each **d**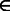 **D**, we calculate a score based on the edit distance e(**d**). We first calculate the size of subset 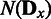, where 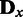 is the subset of **D** all of whose elements have edit distance *x*. Now, for each preferred discordant match pair, a specificity score of **d** is calculated like later in the text:
(6)
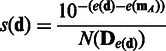

The score is maximum, 1, when the edit distance is the lowest among the all paired match set and there is only one such pair. As edit distance grows, the score decreases asymptotically.

Finally, a breakpoint score is calculated by adding up all scores of corresponding discordant match pairs. Reprever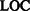 uses 2.0 (which corresponds to at least two unique and unambiguous discordant one-end reads) as a threshold for valid breakpoint.

#### Breakpoint composition

The composition of a breakpoint is described by the size, length and coordinates of the cluster of external ends of one-end mapped reads. The size and length are defined as (respectively) the number of reads included in the cluster and the span (in bp) from the beginning of the first read in the cluster to the end of the last read. For a read with insert size *I* and read length *L*, the length is expected to be 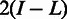. Likewise, the expected size can be easily calculated from the average depth of coverage. Deviation from these expected sizes are suggestive of ambiguous mapping.

Note that forward-strand reads in the cluster should precede all reads on the reverse strand around a true breakpoint (Supplementary Figure S3A). An indication of ambiguous mapping is a cluster in which the forward and reverse strand reads are mixed together in the cluster (Supplementary Figure S3B). This may be caused, for example, when the reference genome already has the homology at the site of predicted breakpoint; this indicates that there is no *true* insertion breakpoint. To quantify this, we classified the discordant one-end reads in a cluster into four classes. Define a potential breakpoint *b* and a match (*p*, *e*) of a read *r*. The read *r* is

1. forward-upstream, where 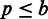 and mapping is forward, *or*
2. reverse-upstream, where 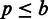 and mapping is reverse, *or*
3. forward-downstream, where 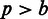 and mapping is forward, *or*
4. reverse-downstream, where 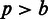 and mapping is reverse.

As described, 1 and 4 support the breakpoint, whereas 2 and 3 do not; moreover, they are counter evidences. We define these reads as conflicting reads.

The conflicting reads are used to penalize the breakpoint score. Briefly, we subtract the score of every conflict read from the breakpoint score (see previous paragraph). If the conflicting reads are unique and unambiguous, it significantly compromises the breakpoint’s confidence. Optionally, we can set a rule for stricter filtration. For example, filtering out all the breakpoints in which any conflict reads exist leaved only 1267 breakpoints from 4533 searched from NA18507.

### Pipeline for Reprever
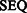


Reprever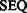 takes Reprever
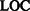
’s output (candidate breakpoints and corresponding paired-end reads) to reconstructs sequences of inserted duplicates, as well as every homologous region 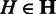 by re-distributing initially mapped mate pairs. We will use a term duplicon to indicate any donor region from given homologs or predicted duplicates, which will be reconstructed by Reprever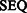 at the end. A brief outline of Reprever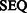 is provided in Figure 1 Box 2 and Supplementary Figure S9. To reconstruct the sequences, it models each duplicon using PHMMs. The algorithm iteratively partitions and aligns mate pairs to one of the PHMMs that has the highest likelihood of generating it. However, as some untrained PHMMs are initially identical, the initial assignment is not straightforward. Fortunately, we can differentiate boundary regions using discordant-one-end reads; these have one read anchored to (presumably unique) flanking regions (Supplementary Figure S9A). Therefore, the initial model parameters of the PHMMs are trained using the discordant-one-end reads. The steps are

Algorithm Reprever
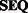

**Input:** Copy count increased region and homologs 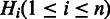, putative insertion breakpoints 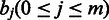, paired-end mapping of donor sequences

**Output:** Consensus sequences of each duplicon.

1. *ClassifyReads*: Partition one-end reads to get a subset 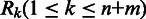 for each duplicon.

2. *CreatePHMMs* 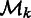, one for each duplicon.

3. **while** (**not** *TerminatingCondition*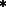)

4.  **do** *Retrain:* for each 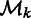: train parameters using *R_k_*.

5.   *Recruit*: for each unassigned two-end read *r*, assign *r* to 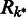 s.t.

6.   
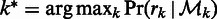

7. **for** each 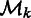

8.  **do** report the ML sequence *I_k_*.

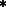
See later in the text for further definition.

#### Creating initial PHMMs

We start with a reference sequence of each duplicon as the template. When there is no reference sequence available (e.g. insertion breakpoints), a subsequence of the longest duplicon is used; we not know which exact homolog is copied to the breakpoint before reconstructing its sequence. The start and end position of the subsequences are deduced from the mapped locations of the discordant one-end reads (Supplementary Figure S10). We model each duplicon with a profile HMM, described by the tuple 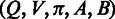 referring to states, symbols, initial state distribution, transition and emission probabilities, respectively. The states include match states, one for each nucleotide of the duplicon, as well as insert/delete states to allow gaps. The output alphabet consists of a single nucleotide (‘A’, ‘T’, ‘G’ and ‘C’), as well as combinations (e.g. ‘M’ = ‘A’ or ‘C’, ‘R’ = ‘A’ or ‘G’ and ‘N’ = any nucleotide) and an empty character (null). The emission and transition probabilities are initialized based on a single observation of the template sequence (Supplementary Method).

#### Retraining PHMMs

Assume that a donor chromosome has *n* + *m* copies of *H* where *n* is the number of pre-existing homologs and *m* is the number of newly inserted copies in the donor. All the mate pairs initially mapped to any of *n* homologs are recruited and partitioned into subsets *R_k_*(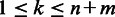). First, concordant-one-end reads from *n* homologs are assigned to the corresponding *R_k_*. Second, each discordant-one-end read *r* is assigned with respect to its external read mapping; if the external read is mapped to flanking regions of *i_th_* homolog or *j_th_* breakpoint, *r* is assigned to *R_i_* or *R_j_*, respectively. After the initial assignment, the reads in *R_k_* are used to retrain the PHMM 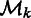.

We apply the read re-alignment procedure (see the corresponding description in Reprever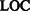 section) to increase sequence coverage and reduce false assignments that comes from mapping ambiguity. The initial whole genome mapping allows at most 5–6 bp difference from the reference genome, which results in massive orphan reads at some duplicates with additional variations. In contrast, a random mapping among equally scored positions (e.g. Burrows-Wheeler Aligner (BWA)) often causes false discordance in mate pairs, which originated from a same homolog; this should be classified to two-end reads. Before training, we could resolve majority of this ambiguity by local realignment. After initial mate pair assignment, we collected orphan and discordant one-end reads to perform pairwise alignment (Smith–Waterman) against the reference sequences of the duplicons. As shown in Supplementary Figure S4, newly aligned mate pairs are reassigned by following criteria:

- *Orphan-one-end* 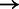 *two-end-reads*: the unmapped (external) read is alignable (see ‘Read Re-alignment’ section) to any 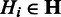.
- *Orphan-one-end* 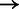 *concordant one-end-reads*: the unmapped read is alignable to any 1-kb flanking regions *L_i_* or *R_i_* where the mapped (internal) read is mapped to *H_i_*.
- *Orphan-one-end* 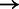 *discordant one-end-reads*: the unmapped read is alignable to any 1-kb flanking regions *L_i_* or *R_i_* where the mapped read is mapped to 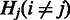.
- *Discordant-one-end* 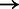 *two-end-reads*: the external read, which originally mapped to *H_j_* with its mate (internal) mapped to 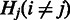, is comparatively alignable to *H_i_* with respect to *H_j_*. The comparativeness can be defined by a maximum difference of sequence similarity/SW score between two different templates.

Training a PHMM includes updating emission and transition probabilities at the site of new observations. Assume that we observed *O_k_* time of an alphabet *v_k_* at a given match state. The total number of observations *O* is 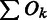. Given a set of background observations *c_k_*, the general form of the emission probability function is
(7)
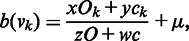

where 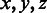 and *w* are weight variables. The updated emission probability 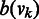 after *O* times of observation should satisfy following constraints.
(8)
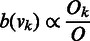

(9)
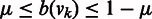

(10)
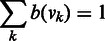

Equation (8) constrains the emission probability to be proportional to the observation frequency. Equation (9) forces the emission probability for each alphabet to converge before 0 or 1 with regard to the sequencing error rate *μ*. The sum of the probabilities for all possible emission should be 1 [Equation (10)]. Using the constraints, the general emission probability formula (Equation 7) can be rewritten (Supplementary Method)
(11)
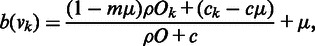

where ρ is the training rate and is empirically trained. Transition probabilities can be trained in a similar way (see Supplementary Methods for detail).

#### Recruiting reads

The initial training of PHMMs using discordant-one-end reads in *R_k_* provides the essential differences in the duplicons at the boundaries. Define the frontiers of each PHMMs as the two regions within which the PHMMs have been trained.

In the recruiting step, we consider the ends of a two-end read 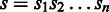 that lies within the trained frontiers and assign *s* to 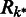 where

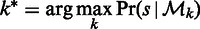


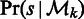
 can be computed using the Viterbi algorithm. Let 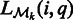 denotes the probability of emitting the first *i* symbols of *s* and ending in state *q*. Then,
(12)
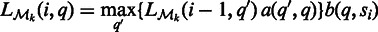

whereas 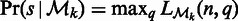. The main trick is in the initial setting. We use the mapping of *s* to *H* to enforce a subset of initial states where *s*_1_ is emitted. This helps speed-up the alignment with no loss of accuracy. Finally, *s* is assigned to 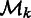 only if (i) it is the ML solution and (ii) the probability exceeds a minimum threshold. Thus, at each stage, some reads remain unmapped, to be considered in subsequent stages.

#### Terminating conditions

There are two terminating conditions: when all two-end reads are assigned to profile HMMs and when there are no two-end reads newly assigned after a round of iteration. The first condition implies that all the PHMMs are separable by existing two-end reads. The second condition usually occurs when there is a non-separable region (e.g. a few hundred base pairs of identical region that is shared by multiple duplicates) causing a lack of variation seeds, which extend the trained region and moves the frontiers toward center. In the second condition, Reprever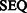 stops the training procedure and reports the most likely sequence inferred so far.

### Data preparation and processing

Here, we briefly describe the procedure for data preparation and processing. For the complete information, please refer to the corresponding Supplementary Method sections.

#### Simulated data and test

One thousand artificial reference ‘genomes’ R were constructed as follows: each genome had five, 50 kb ‘chromosomes’, selected at random from chr1 of hg18. For each genome, an artificial donor genome 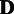 was generated by introducing up to four copies of a random 1–3 kb duplicon (*D_i_*). Each duplicon allows additional variations in both of size and sequence.

To evaluate accuracy in simulated data, we compared each inferred duplicon sequences *I_i_* against the true (but unknown) donor duplicons *A_i_* using BLAST (BLASTN ver. 2.2.25) to compute %-identity, aligned lengths and bit scores. The accuracy of breakpoint location was also calculated from positional difference between the true and inferred breakpoints. Finally, we classified each instance according to the similarity between the template sequence *A_H_* and *A_i_* to investigate performance as a function of divergence.

#### Sequencing data

Paired-end sequencing (SRX016231, Illumina 100 × 2 base and 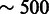-bp insert, 40×) of Yoruban individual (NA18507) was downloaded from NCBI’s Sequence Read Archive website (http://www.ncbi.nlm.nih.gov/sra?term=SRX016231). Human genome reference assembly (NCBI36/hg18) was downloaded from UCSC Genome Browser. To map the sequencing data to the reference genome, a paired-end mapping version of BWA was used. All parameters were set to default except a maximum allowed edit distance. Another whole-genome sequencing data set (SRR034939, 150 × 2 base and 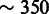-bp insert, 20×) of European individual (NA12878) was downloaded from the Sequence Read Archive (SRA) and processed similarly.

#### Candidate copy number increased regions

Three independent CNV call sets were used as Reprever input. First, a CNV list of 500 regions was downloaded from the online version of the study (24). We selected 100 regions marked with ‘duplication’ out of 500 and further reduced to 85 by filtering out short (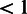 kb) duplications. Second, a list of 206 regions that are predicted to have absolute copy number bigger than 2 (CN 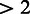) in NA18507 genome was downloaded from the project website (http://www.sanger.ac.uk/research/areas/humangenetics/cnv/highres_discovery.html) (25). A third data set was generated by running CNVer v0.8.1 (4) on the NA18507 sequencing data set. As required by CNVer, we first re-mapped the reads using bowtie to report all good alignments using the suggested parameters. We ran CNVer with parameter min_mps = 5, thus requiring a cluster to contain at least five supporting mappings. Of the initial 5325 CNVs, we selected 2022 copy number increased regions (relative donor copy change >0) and further reduced to 1876 long (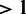 kb) regions.

#### Fosmid clone sequences and validated insertions

A data set of 226 fully sequenced fosmids from NA18507 was downloaded from NCBI GenBank via Human Genome Structural Variation Project homepage (http://hgsv.washington.edu/). We conducted the following procedures to discover insertion sequences that are contained in the fosmid set. (i) Run Blat for each fosmid sequence against the hg18 reference genome to determine mapping sites. (ii) Classify the fosmid match into four classes: normal, deletion, insertion and others. (iii) For insertion matches, extract exact location and sequence and (iv) query inserted sequences to hg18 to check novelty and the type of sequences. Detailed procedure can be found in the Supplementary Method. Another data set of 454 fully sequenced fosmids from NA12878 was downloaded and processed similarly to discover 74 insertions. Of 74, we found seven were unambiguously duplicated (61 novel, 6 undetermined).

## RESULTS

### Performance test using simulated data

The performance of Reprever was tested on 1000 artificial test genomes each of which consists of five 50 kb chromosomes (extracted from Chromosome 1 of hg19) and manipulated to contain up to four duplicates of a randomly selected block. We applied Reprever pipeline to this test set and tested on (i) accuracy of breakpoint number and position and (ii) accuracy of reconstructed sequences (see ‘Materials and Methods’ section for details).

**Figure 2.**
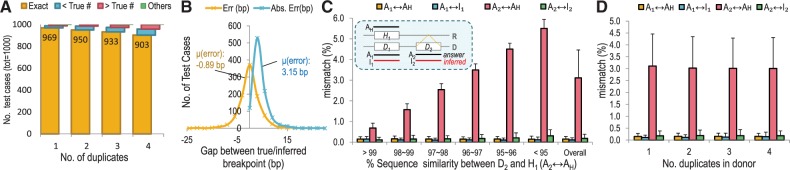
Performance test of Reprever in breakpoint and sequence inference. Totally 1000 simulated genomes are constructed and underwent random duplication up to four copies. (**A**) Reprever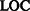 identified up to 97% of the breakpoints. In >90% of four copy insertion cases, Reprever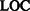 exactly found insertion sites. (**B**) Distribution of error between true/inferred breakpoints. Errors <0 denotes that the inferred breakpoints are located upstream of true breakpoints, errors >0 denotes downstream. The average error size is 3.15 bp. (**C**) Accuracy of reconstructed duplicate sequences in 969 test genomes. Comparisons between the true and inferred sequences (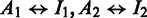) are measured in sequence dissimilarity using BLAST. Compared with the reference sequence *A_H_*, which is the best estimate sequence for extra copies without reconstruction, Reprever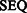-inferred sequences have much less mismatches to the true answers (green bars). The performance is consistent regardless of initial diversity among duplicons (red bars). (**D**) Reprever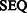 can reconstruct multiple duplicates simultaneously without an accuracy loss.

The exact number of breakpoint could be inferred in 93.9% of test donor genomes. In 90.3% of the cases with four duplicons, Reprever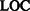 identified all breakpoints; the number improved to 97% going with *n* = 1 duplicons (Figure 2A). The average size of gap between actual and inferred breakpoint position was 3.15 bp, predicting >98% of breakpoints in 15-bp error (Figure 2B). For the test genomes whose breakpoints are successfully identified, we used Reprever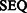 to reconstruct the duplicon. Let *A_H_* represent the sequence of the high CNV region *H*. In the donor, we have up to four mutated copies of *H*, each represented by *D_i_*. Let *A_i_* and *I_i_* represent the true, and reconstructed, sequences, respectively, of the *i*th duplicon. Reprever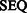 reconstructed each *I_i_* from the paired-end mappings of the donor to the reference.

Figure 2C shows the results on the consensus reconstruction of the duplicons in 969 test cases (*n* = 1) each with a single duplication in the donor. The inferred and true duplicons (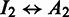) have only 0.18% mismatch rate on average versus the original template (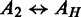), which has 3.11%. Thus, Reprever resolves 94.2% of sequence discrepancy (Figure 2C pink versus green bar). Moreover, reconstruction influenced sequence accuracy of orthologous regions (e.g. *D*_1_) by re-distributing reads into their origins to accept only relevant variants. Reprever could successfully reconstruct multiple duplicates without losing accuracy (Figure 2D).

Two factors affect reconstruction. If duplicons are too similar (nearly identical), differentiating among the duplicate sequences is hard. Alternatively, if the similarity is too low, reads from the duplicated regions are not likely to be mapped in the reference, suggestive of an insertion, rather than a duplication. As shown in Figure 2C, Reprever infers the duplicons with high accuracy (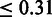
% divergence) regardless of the degree of initial divergence (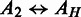).

### Validation with sequenced fosmid clones

**Figure 3.**
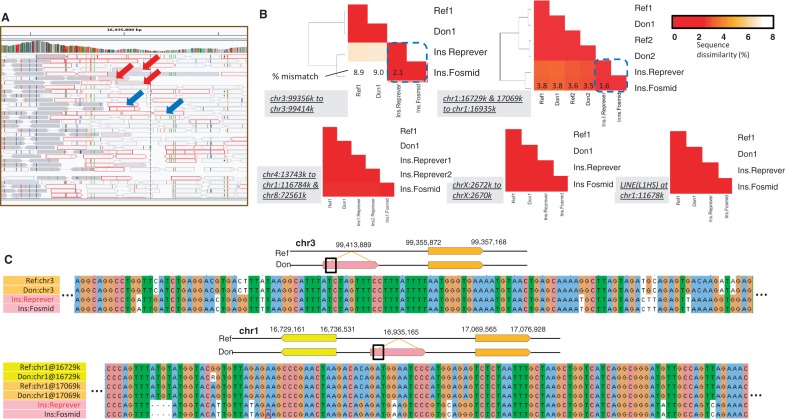
Reconstruction of fosmid-validated duplications. Totally 1000 simulated genomes are constructed and underwent random (**A**) (discordant) paired-end reads mapped around a validated insertion site chr1:16935160 (from chr1:17069565–17076928 and chr1:16729161–16736531). There are only a few discordant reads that bridge the insertion site to its template (red arrows). Reprever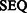 automatically recruits neighboring orphan reads (blue arrows) to increase read coverage for reconstruction. (**B**) Comparison of reconstructed and true (fosmid) insertion sequences. The reconstructed sequences (Ins.Reprever) are much closer to the true insertions (Ins.fosmid) compared with the template sequences (Ref1 and Ref2). Reconstructed donor sequences at the template sites (Don1, Don2) are almost identical to the templates. (**C**) Multiple sequence alignment visualization of sequence variation between templates and reconstructed sequences. Highly varied regions including deletions are reconstructed perfectly.

We tested Reprever’s ability to reconstruct duplicated insertions using high quality completely sequenced fosmid clones, which have been used in the past to confirm many structural variations. Although high quality, the available fosmid data are sparse and provide only a handful of examples. We downloaded 226 fosmid clones generated from a Yoruban genome (NA18507, library ABC8) from GenBank via Human Genome Structural Variation Project (http://hgsv.washington.edu/). We found 16 insertions from the 226 fosmid clones (see ‘Materials and Methods’ section). The 16 insertions include four previously reported insertions by Kidd *et al.* (26), who used the early version of the fosmid data (22 fosmid clones). Comparing against the reference using Blat (27) revealed that 12/226 (4/22) were novel insertions that were not duplicated, two undetermined because of clone mapping ambiguity (see Supplementary information for details), leaving two for Reprever analysis. Note that, the breakpoint regions were highly noisy and require extensive orphan rescue procedures that are supported by Reprever (Figure 3A). The two duplicated regions were reconstructed by Reprever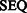.

We next downloaded 454 complete fosmid sequences from the European (CEPH) individual NA12878 (library ABC12), chosen for availability of both fosmid clones, as well as high-coverage paired-end sequencing data (SRR539363, 150 × 2 paired-end read, 350 insert size, ∼20× coverage). Kidd *et al.*. have reported 30 non-Variable Number Tandem Repeat (VNTR) insertions in NA12878 (28). We analyzed these 30 insertions, as well as an additional 44 insertions derived from the 454 sequences (‘Materials and Methods’ section). Of these 74, we found 61 to be novel, 6 undetermined and 7 to be duplicated. Reprever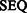 was used to successfully reconstruct 6/7 of duplicated sequences leaving one tandem duplication. The full list of analyzed fosmid clone is available in Supplementary Data set S1.

We tested how the reconstructed sequences diverged from their original templates. Figure 3B shows sequence dissimilarity matrices among the template and reconstructed sequences. BLASTN is used to calculate the sequence identity. The assembled sequence (Ins.Reprever) variates from its original template (Ref1 or Ref1 to RefN when multiple templates are used). Note that Reprever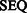 also reconstructs donor sequences at the template sites as well (Don1 to DonN). The reconstructed sequence of the 1.2-kb insertion at chr3:99413889 of NA18507 (Figure 3B upper left) showed 97.9% similarity with the fosmid clone, which has been greatly increased from 91.1% of the template sequence. Note that the Don1 remains almost unchanged to make a clear cluster shown in the dendrogram. Similarly, the 6-kb insertion at chr1:16935165 of NA12878 (Figure 3B upper right) was successfully reconstructed achieving 98.4% sequence similarity to the fosmid (from 96.2% of Ref1 and 96.4% of Ref2). In other cases, including repeat element (LINE) insertion, Reprever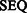 hardly changed template sequences, which turned out the inserted sequence is almost identical to its template (Figure 3B lower and Supplementary Table S2 for details).

Closer inspection of the variated regions confirms the accuracy of reconstruction. We ran multiple sequence alignment of the sequence groups using clustalW (29). Figure 3C depicts the most variable sites of the two reconstructed sequences. As shown, most variations were perfectly corrected including a 4-bp deletion (Figure 3C lower left). We also found that the remaining mismatches after reconstruction (1∼4%) can be explained by the unreachable central region bounded by long inseparable sequences. We expect that longer read length and insert size will increase the coverage to resolve remaining regions.

### Application to NA18507 genome

The Yoruban individual NA18507 has been re-sequenced and analyzed many times and serves as a useful template for duplicon discovery and reconstruction. We used four independent gain call sets for initial input of Reprever: (i) 1876 copy number increased regions predicted by CNVer in this study (SRX016231, 2 × 100-bp reads, 40× coverage), (ii) 85 previously predicted regions on Chr 1 by Yoon *et al.*. (24), (iii) 206 regions predicted to have absolute copy number greater than two using high-resolution oligonucleotide array by Conrad *et al.*. (25) and (iv) 1000 randomly selected regions sized to match the CNVer call set (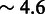 ± 0.5 kb) as a negative set (see ‘Materials and Methods’ section for preparation procedure and Supplementary Data set S2 for the whole list). Note that the array-based call was made using different reference genome (NA10851, a CEPH male), and the absolute copy number may not indicate a relative copy number increase against hg18 (e.g. the reference genome may also have increased copies).

First, we attempted to discover the location of extra copies that originated from the given regions (see ‘Materials and Methods’ section). Reprever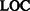 identified 4533 (4102, 174, 180 and 77 from CNVer, Yoon, Conrad and random call set, respectively) breakpoints from 1263 (1103, 54, 67 and 39 like earlier in the text) of the given regions (Table 1). Although there is no true gold standard of high-CNV regions, the significantly lower breakpoint acceptance rate of the random set (3.9%, 39/1000) compared with the other (58.8, 63.5 and 32.5%) indirectly represents the robustness of Reprever in filtering potential false-positive CNV calls. Conversely, the use of commonly called regions significantly increased the breakpoint finding rate. In particular, we assume that the high resolution (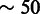 bp) of the array-based call set can provide more exact boundary of duplication events when combined with other call sets. As shown in Table 1, Reprever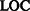 accepted 10 of 11 (90.9%) of Conrad calls that are also predicted by CNVer and Yoon. Although Reprever does not evaluate given CNV inputs to be true or false, the positive correlation between the rate of breakpoint discovery and the level of evidences shows that the availability of highly accurate CNV regions would increase the chance of finding the insertion location of their extra copies.

**Table 1.** Reprever analysis on NA18507 data given three independent gain calls and one negative call

Call set (size)	n.accepted (rate)	n.breakpoint
Each call set		
CNVer (1876)	1103 (58.8%)	3993
Yoon^a^ (85)	54 (63.5%)	168
Conrad (206)	67 (32.5%)	173
Random (1000)	39 (3.9%)	77
Overlapping call set^b^		
CNVer + Yoon (27)	22 (81.5%)	63
CNVer + Conrad (96)	55 (57.3%)	194
Yoon + CNVer (16)	9 (56.3%)	30
Yoon + Conrad (3)	3 (100.0%)	11
Conrad + CNVer (9)	8 (88.9%)	9
Conrad + Yoon (2)	2 (100.0%)	2
CNVer + Yoon + Conrad (2)	2 (100.0%)	4
CNVer*Yoon (12)	10 (83.3%)	13
CNVer*Conrad (9)	8 (88.9%)	24
Yoon*Conrad (2)	2 (100.0%)	3

For each call set, Reprever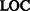 scans potential insertion breakpoints of the given regions.

n.accepted, number of regions that have at least one matching breakpoint; n.breakpoint, number of total breakpoints.

^a^This call set was targeted only to Chr 1.

^b^We define an overlapping call set 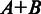 as the subset of call set *A*, which takes every region 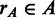 in the condition of existence of a region 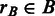, which overlaps at least 50% of *r_A_*. Overlapping call set 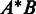 is similarly defined but contains regions where the overlap is satisfied reciprocally. In other words, at least 50% of *r_B_* is also in *r_A_*, and vice-versa.

As described previously, we identify other (homologous) regions on the reference genome for each given region (Figure 1 and ‘Materials and Methods’ section). We found that the consideration of homologs including read recruitment from the homologous regions significantly increased read coverage around the breakpoint. Of 906 594 discordant paired-end reads anchored around breakpoints, 78.8% (713 948) were collected from homologs. For example, a duplication of chr1:553585–560412 into chr8:63177985 could be reconstructed with higher coverage by considering a homolog (chrM:3915–9756) of the given region (Supplementary Figure S1). In this case, 70.6% (24/34) of discordant one-end anchored reads were collected from the homologs to provide richer information about breakpoint location and sequence.

Each predicted breakpoint was scored by Reprever using several independent features. We used the (i) quantity, (ii) specificity and (iii) composition of discordant one-end reads to calculate scores (see ‘Materials and Methods’ section and Supplementary Figures S2 and S3). Briefly, each discordant read was highly weighted if it is the only possible alignment in the genome. Otherwise, it was penalized by the matching accuracy (edit distance) and the number of other possible mappings. The composition represents coordinates and positions of discordant reads around breakpoints. Intuitively, we expect all the anchored reads in the upstream of a breakpoint to be mapped forward, whereas the downstream reverse. Reads of conflict coordination were used to penalize the breakpoints. This score can be used for further prioritization of discovered breakpoints. For example, we automatically prioritized 1267 (1153, 40, 74 from CNVer, Yoon and Conrad) highly confident breakpoints with good (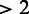) score and perfect (no conflicting reads) composition for sequence reconstruction.

We tested the potential impact on genes caused by duplicon insertions. Not surprisingly, a majority of the breakpoints (81.3%, 1017 of 1267 highly confident ones) are intergenic. However, in some cases, the insertion breakpoint location is directly within a gene and may impact the phenotype (Table 2, full list in Supplementary Table S3). In many of these cases, the CNV and its insertion breakpoints are clustered in regions with known duplications, suggesting non-allelic homologous recombination (NAHR) as the mode of CNV formation. For example, we observed four duplication events on *1q21.1* inserting into genes from the neuroblastoma breakpoint (NBPF) family (Table 2). CNVs in this region have been shown to produce aberrant transcripts in the NBPF family and increase susceptibility to neuroblastoma (30). We detected even more complex signatures that cause alternative transcripts with potential copy number change. For example, we found a clear breakpoint signature between the second and third exon of *MUC20*, which encodes a member of mucin protein family and has multiple transcript variants. A previous study reported CNVs in this region among different ethnicities (6). We observed a gene conversion between two exons, which is potentially caused from an NAHR within its segmental duplication pair that explains the accompanying copy number changes (Supplementary Figure S7).

**Table 2.** Predicted breakpoints in gene region

Breakpoint	Region	Gene	Score	CNV source
chr1:16766464	Intron	*NBPF1*	62.07	CNVer
chr1:16775357	CDS	*NBPF1*	33.02	CNVer
chr1:16778388	CDS	*NBPF1*	100.07	CNVer
chr1:16784684	CDS	*NBPF1*	37.8	CNVer
chr1:16792907	Intron	*NBPF1*	105.0	CNVer
chr1:21665540	Intron	*NBPF3*	38.91	CNVer
chr1:143665870	Intron	*PDE4DIP*	39.2	CNVer
chr1:143989906	Intron	*NOTCH2NL*	25.3	CNVer
chr1:144008986	CDS	*NBPF10*	43.25	CNVer
chr1:144011688	Intron	*NBPF10*	24.3	CNVer
chr1:144013697	Intron	*NBPF10*	27.01	CNVer
chr1:147009280	CDS	*NBPF16*	22.42	CNVer
chr1:147965460	Intron	*LOC729130*	30.57	CNVer
chr2:91467172	Intron	*LOC389000*	21.46	CNVer
chr2:95963046	Intron	*LOC400986*	37.5	CNVer
chr2:95965655	Intron	*LOC400986*	95.25	CNVer
chr2:95967599	Intron	*LOC400986*	117.56	CNVer
chr3:197148075	CDS	*LOC727978*	26.93	CNVer
chr3:197149586	Intron	*LOC727978*	37.05	CNVer
chr3:197150946	Intron	*LOC727978*	24.0	CNVer
chr4:191100462	Intron	*FRG1*	21.48	CNVer
chr4:191105726	Intron	*FRG1*	33.2	CNVer
chr5:98898838	Intron	*LOC728104*	29.67	CNVer
chr6:24791875	Intron	*ACOT13*	31.0	Conrad
chr9:66729560	Intron	*LOC653458*	38.62	CNVer
chr11:89298719	Intron	*LOC729384*	28.56	Conrad
chr12:69819920	Intron	*TSPAN8*	89.0	Conrad
chr15:26137336	CDS	*HERC2*	25.98	CNVer
chr16:33492794	Intron	*LOC401847*	31.81	CNVer
chr22:18976441	Intron	*LOC729461*	31.7	CNVer

Top 30 scored breakpoints are listed here. The entire list including intergenic regions is available in Supplementary Table S3.

CDS, coding DNA sequence.

Finally, using Reprever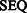, we reconstructed the sequence of 885 insertion locations from 1267 highly confident breakpoints. The reconstructed duplicons ranged between 17 and 18418 bp (average: 1689 bp) in length and contained 37 mismatches (1.4 gaps), for a divergence of 2.2%. These rates are much higher than reported single-nucleotide variation (SNV) frequencies [0.107 and 0.014% for SNPs and indels (31)], suggesting that the majority of the duplications are ancient events.

### Confirmation of reconstructed duplicons

From the highly confident NA18507 duplicons, we selected regions of top 30 scores. Of the 30, in only 10 regions we could design primers for PCR amplification; note that the inserted duplicated sequence with repeat elements around the breakpoints significantly reduces the sequence uniqueness for primers. Although we do not attempt a strict statistical validation of the predicted regions, mainly because of the unavoidable effects from the accuracy of initial CNV calls and sparseness of amplifiable regions, confirmation of the selected regions gives a good practical picture of Reprever analysis. We could identify 9/10 duplicons exist in the NA18507 genome. Here, we will describe four regions in detail by comparing with PCR–Sanger sequencing results (see Supplementary Figure S12 for full result).

#### CNV duplicons

**Figure 4.**
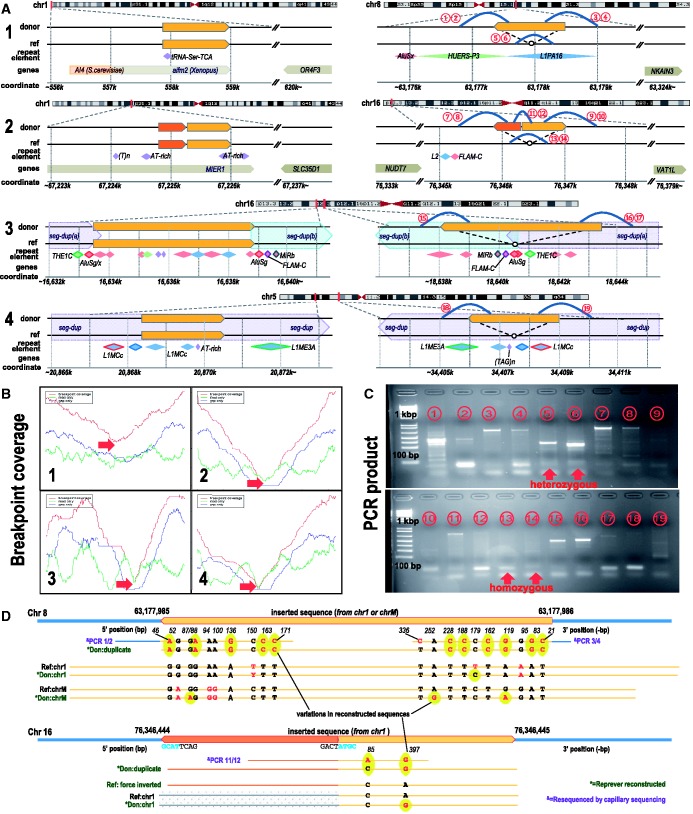
(**A**) Four cases are illustrated as examples. (1) A gain call is made at chr1:553585-560412 by CNVer (and chr1:554301–560300 by Yoon *et al.*). Reprever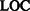 found that a ∼1.3-kb sub-region is duplicated into chr8:63177985. (2) Similarly, a gain call at chr1:67224800–67226000 by Yoon is explained by Reprever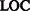 showing a duplication at chr16:76346444. Note that the inserted sequence has a partial inversion of 400-bp 5′ segment (orange block). (3 and 4) Two gain calls were made CNVer (chr16:16633477–16638823 and chr5:20868352–20870585). Insertion locations found by Reprever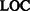 are located in bigger segmental duplication regions (dotted blue/red blocks). The arrangement of repeat elements (colored diamonds) show that the copy number increase in the donor genome actually resulted from deletions in the reference genome. (**B**) Concordant read counts around the putative breakpoints (red arrows). Coverage is calculated using whole-clone (red), end reads (green) and the gap between end reads (blue) of mate pairs. The relative read count reduction predicts the allele zygosity (1/3 heterozygous and 2/4 homozygous). (**C**) PCR amplification of duplicated regions. Primers are designed to capture duplication boundaries (encircled red numbers in A). (**D**) (upper) The inserted sequence of the first case (at chr8:63177988) is identified by Sanger sequencing (annotated with PCR 1/2 and PCR 3/4). Totally 12 SNVs are found. Three consensus sequences reconstructed by Reprever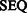 (green) recovered 11 of 12 variations (92%) for inserted sequence and found four additional variations in the homologs (highlighted in yellow). (lower) The partial inversion of the inserted segment in the second case (at chr16:76346444) is confirmed by Sanger sequencing with two SNVs. Reprever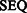 recovered one of them.

Figure 4A shows the four selected region in their genomic context. In the first example, CNVer predicted a copy number increase of a 6.8 kb segment in chr1, and Yoon had also called a 6 kb CNV in a nearby location. In creating the homologous set of reference sequences, Reprever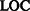 identified a homologous region with 98.7% sequence similarity in the mitochondrial chromosome of the reference (chrM:3915–9756). The Reprever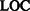 breakpoint analysis predicted an inverted duplication at chr8:63177988 landing on a LINE element (L1PA16). However, the reconstruction revealed a much smaller inserted segment, comprising only 1.3-kb subregion of the original CNV. We suspect that the original CNV call was due to the mtDNA duplication.

In example 2, a 1.2-kb CNV region was identified to be unique in the reference. Using Reprever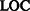, an insertion breakpoint was identified in chromosome 16 (chr16:76346444). Although the breakpoint signature was clean, there was an abnormal pattern of paired-end mappings within the CNV. Analysis revealed it to be a partial 400-bp inversion in the duplicon (orange blocks in Figure 4A2). Using the reference CNV sequence (after inversion) as template, Reprever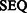 was used to reconstruct the duplicon.

In a similar fashion, the remaining two CNV candidates were analyzed using Reprever. Unlike the previous cases, they are located in known segmental duplication regions (dotted arrow-shaped blocks, Figure 4A3 and 4) making the whole analysis even harder because of the identical sequences around the breakpoint. Nevertheless, the remapping procedure in Reprever’s specificity analysis uses confirms the breakpoints, and by extension, the change in copy number. The arrangements of repeat elements around the original segments and breakpoints suggest that the event is more likely a deletion in the reference genome instead of a duplication in the donor; two repeat element arrangements in Figure 4A completely match except the ∼5-kb deleted region between two AluSg elements (red lozenges), indicating a forward slippage in the replication. We can see a similar pattern in the last case (Figure 4A4).

Read coverage around the four breakpoints are shown in Figure 4B. We see a clear drop of read count at each predicted breakpoint (red arrows). The ML estimate of zygosity (see ‘Materials and Methods’ section) reveals examples 1 and 3 to be heterozygous and 2 and 4 as homozygous.

#### PCR amplification

A redundant set of 19 primer pairs were designed to capture the boundaries of the duplicons (Figure 4C; the corresponding regions are denoted as red encircled numbers in Figure 4A). For example, primer pair 1 consists of one primer sequence (∼25 bp) from the 5′ region upstream of the breakpoint Chr 8 and the other from the negative strand of inserted 1.3-kb Chr 1 segment. Amplification validates the novel insertion. As shown in the figure, we could amplify many products, most of which are the predicted size. In cases where non-specific products were unavoidable, desired products were gel extracted before sequencing (see ‘Materials and Methods’ section).

We also included a few primer pairs to test zygosity. Primer pairs 5/6 and 13/14 span the breakpoints allowing two possible explanations. If the duplication is heterozygous, these primer pairs will be amplified and generate product from one normal (reference type) allele. But in the homozygous duplication, PCR will fail due to the ‘oversized’ insertion between two primers. Both of the primer pairs 5 and 6 gave amplified products, whereas the primer pairs 13 and 14 did not, showing that cases 1 and 2 are heterozygous and homozygous, respectively, as predicted by breakpoint coverage. We could not design zygosity-testing primer pairs for the third and fourth cases because of non-unique sequences.

#### Sanger sequencing

We performed Sanger sequencing on 10 amplified PCR products (region 1, 2, 3, 4, 5, 6, 8, 11, 12 and 16) of 19 regions. The nine remaining regions could not be amplified, or the sequence was of poor quality. All of the confirmed sequences have close to the predicted structure, for example, a partial match to a breakpoint flanking region, followed by the inserted duplicate region. This confirms the PCR products did not arise from non-specific binding or from the amplification of similar genomic regions.

Figure 4D shows the results of multiple sequence alignment among the reference sequences, reconstructed donor sequences and the Sanger sequencing results. The reconstructed sequence shows a dramatic improvement in sequence size and identity. In the first case (chr1:553585–560412), we found 12 SNVs at the PCR products, 11 of which are recovered by Reprever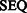 (yellow ovals). We also found four additional SNVs in homolog sequences. The size of the reconstructed duplicate is only ∼10 bp shorter at both ends (∼20 bp in 1.3 kb), which is greatly more accurate than that of the original CNV candidate region (∼6 kb). Note that the alternative, which is to treat the reference CNV region as a representative of the inserted duplicon, would be grossly more inaccurate. The breakpoint location (chr8:63177985) was exactly predicted in Reprever. In the second case (chr1:67224800–67226000), the partial inversion of the inserted segment was confirmed by Sanger sequencing with only a 10-bp positional error. Note a small inverted microhomology around the breakpoints of the inverted region. Reprever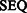 recovered one SNV (G
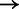
A) of two. The high sequence similarity (99.7%) between the PCR product and the reference region (forced to partially inverted for comparison) shows that the duplication is a recent event. By contrast, most of the other reconstructions have a higher amount of divergence than SNP frequencies, suggesting that the duplication predated the common ancestor, and the duplicon was subsequently deleted in the reference genome.

## DISCUSSION

Reconstructing the sequence of duplicated regions in a donor genome, and identifying the breakpoints where the duplicon is inserted, continues to be a challenging problem. The difficulty increases with multiple duplicons in the donor and in the reference. For example, consider the CNVer prediction of 1-kb region with copy number chaining from two to three in the NA18507 donor. A homolog search reveals 150 reference loci to the region with 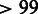
% identity. The number increases with a less stringent definition of homology. Even if the example is extreme, it reveals why it is difficult to predict the locations of CNV expansions.

Although we do not attempt to settle the CNV identification question using Reprever, we do provide a tool for a deeper analysis of a candidate CNV expansion. A key contribution of our approach is that in addition to looking at reads mapping to the CNVs, we locate the insertion breakpoints, and carefully analyze the reads mapping to the breakpoints. We propose three distinct ways of categorizing breakpoints and certifying their validity. By extension, a valid CNV must contain at least one valid insertion breakpoint. The approach helps because the flanking region around the breakpoint is often more specific than the sequence inside the duplicon. As a gold standard of known duplication breakpoints and duplicons is created, we will use these analyses as features to train breakpoint validity.

The case of duplicated insertions is tricky. As the duplicated sequence diverges from the original, it rapidly becomes a ‘pure’ insertion, best assembled by *de novo* methods. In that case, reads sampled from the donor duplicon will not map to the original region, which in turn will not be classified as a CNV expansion. Reprever reclassifies and re-maps reads from the CNV and recruits orphan into a template-driven assembly. Although not as general as *de novo* reconstruction, it provides highly accurate reconstructions for duplicons. In simulations and real data, it does a good job of reconstruction even when the duplicon is highly diverged. Nevertheless, it is possible that we miss the reconstruction of other diverged duplicons at the boundary, specifically when they contain larger insertion/deletion events as well. Our future work will investigate this using more sophisticated simulations and other sequenced genomes.

Finally, the detailed impact on phenotype is potentially important. Recent work has uncovered associations between CNV expansions and diseases. By investigating where the duplicons inserted (do they disrupt or mutate functional regions) and what they contain (is an entire gene and its regulatory sequence duplicated?), we can provide a deeper insight into the causal mechanism for associations. Such discoveries will help with a deeper understanding of the role of CNVs in regulating diversity and disease.

## AVAILABILITY

The packaged software of Reprever with source codes and example data is available at https://github.com/sak042/Reprever.

## SUPPLEMENTARY DATA

Supplementary Data are available at NAR Online: Supplementary Tables 1–5, Supplementary Figures 1–12, Supplementary Methods, Supplementary Data sets 1 and 2 and Supplementary References (32–38).

## FUNDING

Funding for open access charge: National Institute of Health [U54-HL108460, 5R01-HG004962]; National Institute of Child Health and Human Development [1P01HD070494-01]; National Science Foundation [CCF-1115206].

*Conflict of interest statement*. None declared.

## Supplementary Material

Supplementary Data
