# Supplementary material for: Reprever: resolving low-copy duplicated sequences using template driven assembly
Source: Nucleic Acids Res. 2013 May 8;41(12):e128. doi: 10.1093/nar/gkt339 (PMC3695505; doi:10.1093/nar/gkt339)
Supplement: Supplementary Data [file supp_41_12_e128__index.html]

Reprever: resolving low-copy duplicated sequences using template driven assembly — Reprever: resolving low-copy duplicated sequences using template driven assembly — Supplementary Data 

# Reprever: resolving low-copy duplicated sequences using template driven assembly

## Supplementary Data

files

**Files in this Data Supplement:**

- Supplementary Data - pdf file
- Supplementary Data - xlsx file
- Supplementary Data - xlsx file
