# Supplementary material for: Reprever: resolving low-copy duplicated sequences using template driven assembly
Source: Nucleic Acids Res. 2013 May 8;41(12):e128. doi: 10.1093/nar/gkt339 (PMC3695505; doi:10.1093/nar/gkt339)
Supplement: Supplementary Data [file supp_gkt339_nar-00468-n-2013-File006.pdf]

Supplementary Information for "REPREVER: Resolving low-copy duplicated sequences using template driven assembly"

Sangwoo Kim[1], Paul Medvedev[1], Tara A. Paton[2], and Vineet Bafna[1*]

[1]Department of Computer Science and Engineering, University of California, San Diego, 9500 Gilman Drive, La Jolla, CA, United States

[2]The Centre for Applied Genomics, The Hospital for Sick Children, 101 College Street, Toronto, ON, Canada

e-mail:[1]{sak042, pmedvedev, vbafna}@cs.ucsd.edu, [2]tpaton@sickkids.ca

[*]To whom correspondence should be addressed

Contents

1 Supplementary Figures **2**

2 Supplementary Tables **15**

3 Supplementary Methods **32**
3.1 Parameter initialization for profile Hidden Markov Model 32
3.2 Parameter training for profile Hidden Markov Model 32
3.3 Training transition probabilities 33
3.4 Prediction of breakpoint zygosity 33
3.5 PCR amplification of duplicated regions: 34
3.6 Sanger sequencing of duplicons: 35
3.7 Complete information of data preparation and processing 35
3.7.1 Simulated data and test: 35
3.7.2 Sequencing data: 35
3.7.3 Candidate copy number increased regions: 36
3.7.4 Fosmid clone sequences and validated insertions 36
3.8 Comparison with split-read based algorithms 36

4 References **38**

List of Figures

S1 One-end anchored reads from different homologs forming an insertion location 3
S2 Breakpoint specificity 4
S3 Breakpoint composition 5
S4 Analysis on unmapped reads 6
S5 Classification of paired end mapping with respect to a high copy region H 7
S6 A landscape of copy number increase in NA18507 8
S7 Complex copy number variation in *MUC20* 9
S8 Zygosity test for breakpoint 10
S9 An overview of duplicate identification process 11
S10 Getting a template sequence of a duplicate (D_2) from the original copy (H) 12
S11 An example profile HMM for sequence modeling 13
S12 PCR experiments on high confident NA18507 duplicons 14

List of Tables

S1 Prior studies for long ($>$ 1kb) insertion identification 15
S2 Accuracy of Reprever reconstructed sequences of fosmids 16
S3 Breakpoints in gene region 17
S4 Primer pairs for PCR amplification 31
S5 Comparion with split-based algorithms 37

1 Supplementary Figures

Figure S1: One-end anchored reads from different homologs forming an insertion location. One-end anchored reads (*discordant-one-end* reads, see Methods) from the originally predicted region (Chr 1, blue arrows) and its homolog (Chr M, beige arrows) form a complementary cluster at Chr 8. Increased coverage around breakpoints improves breakpoint accuracy and reconstruction performance.

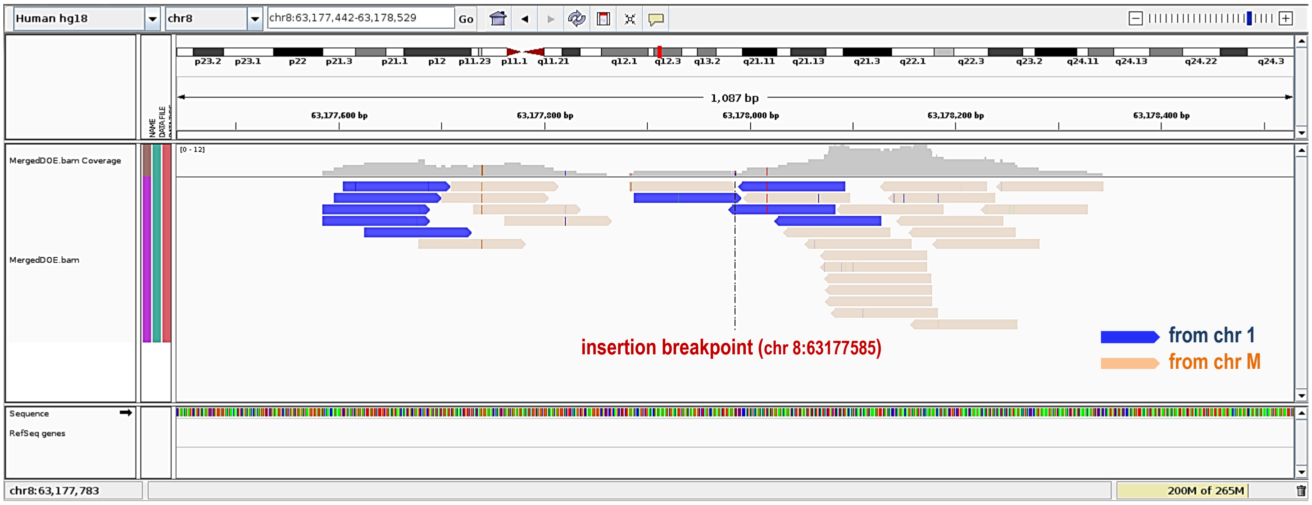

Figure S2: Breakpoint specificity. Mapping around the breakpoint is confounded by non-unique flanking sequences. A. A breakpoint (right yellow dot) can be detected by the discordant-one-end reads mapped around it. However due to the homologous sequences (L_1, L_2), the signal around the breakpoint (L_3) can be weaken (blue arrows at L_1 and L_2). B. An example of a *false* breakpoint signal due to the non-unique flanking sequences. A clean shaped cluster (at D_3) is formed simply by mapping ambiguity around, but there is no actual duplication.

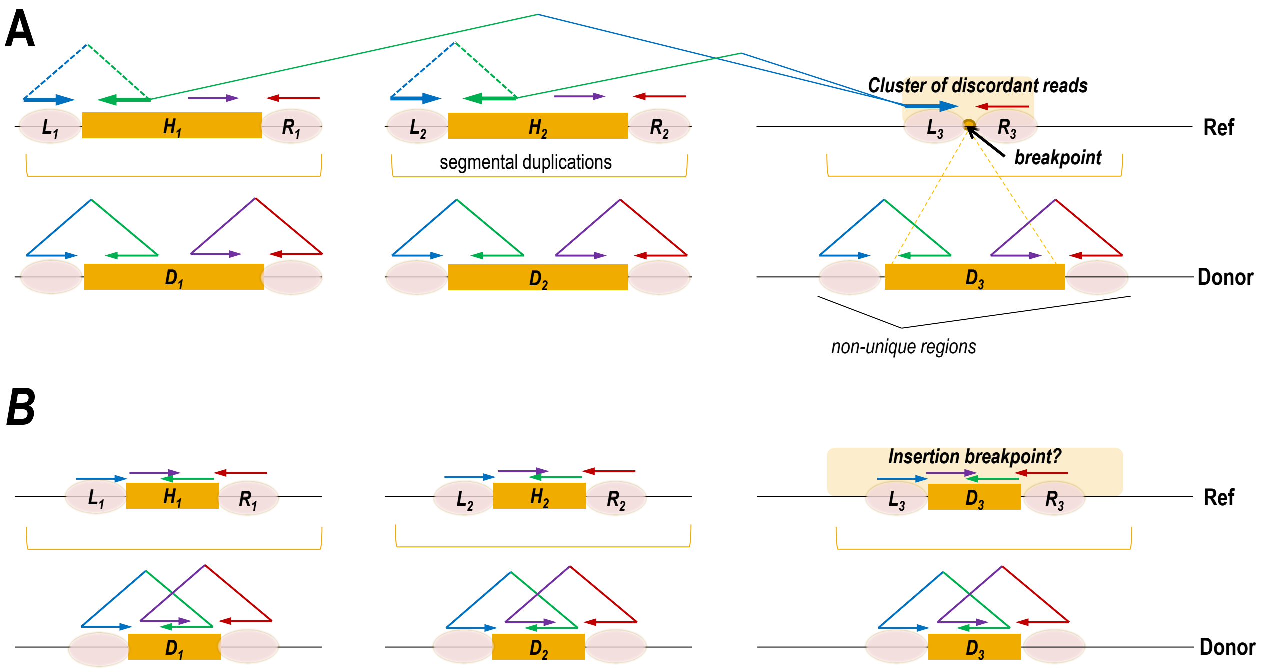

Figure S3: Breakpoint composition. Discordant-one-end reads form clusters around insertion locations. A. A standard clean shaped cluster is shown around a true duplication breakpoint (yellow dot). When the flanking sequences are unique (pink ovals), all the forward reads (blue arrows) are mapped just prior to the breakpoint, and reverse strand read (red arrows) just posterior to it. B. A mixed shaped cluster can be generated at a *false* breakpoint, where the reference genome has the same sequence(H_2) at the locus. We can see some forward reads (blue arrows) are mapped at the 3' region of the block (just from mapping ambiguity caused by sequence similarity to H_1), and reverse reads (red arrows) at the 5' region. C. The overlap length is defined as the span between the leftmost reverse-strand read and the rightmost forward-strand read. The cluster size is defined as the span from the beginning of the first read to the end of the last read. Using these two measures, we can classify the clean shape from the mixed shape.

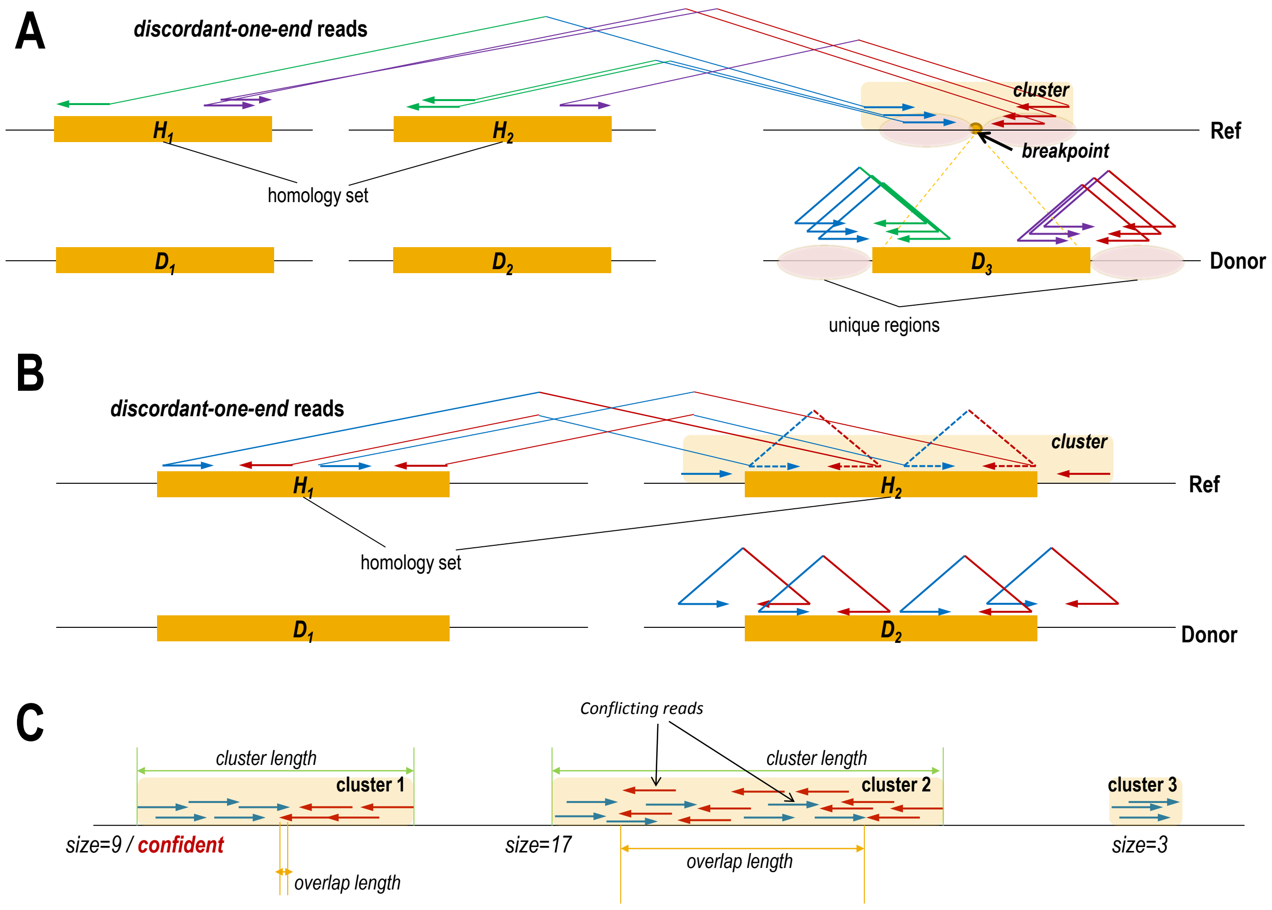

Figure S4: Analysis on unmapped reads. A. Possible causes of unmapped (*orphan-one-end* reads are depicted. Reads (red arrows) sequenced from additional variations (e.g. SNPs, micro-insertions/deletions), repeat elements (e.g. SINEs, LINEs) and/or across breakpoints can be unmapped in the whole genome mapping. Reads with bad sequencing quality (e.g. bad Phred quality score) can be also unmapped. B. Rescued unmapped reads from $\text{REPREVER}_{\text{LOC}}$. Reads with a certain sequencing quality (less than 10 nucleotides with <Q20 Phred Score) are aligned pairwise to each $H \in \mathbf{H}$ and its flanking regions; reads of >92 similarity and >400 SW score are rescued and reassigned. Unlike our expectation, bad sequencing quality is the most important cause of mapping loss. Only 0.7% orphan reads came from non-homologous regions. C. The portion of repeat elements in unmapped reads are analyzed using RepeatMasker. Even in unmapped reads, the portion is not much higher than that in mapped reads (57% versus $\sim$ 50%) meaning that repeat elements in flanking regions are not a major cause of mapping loss.

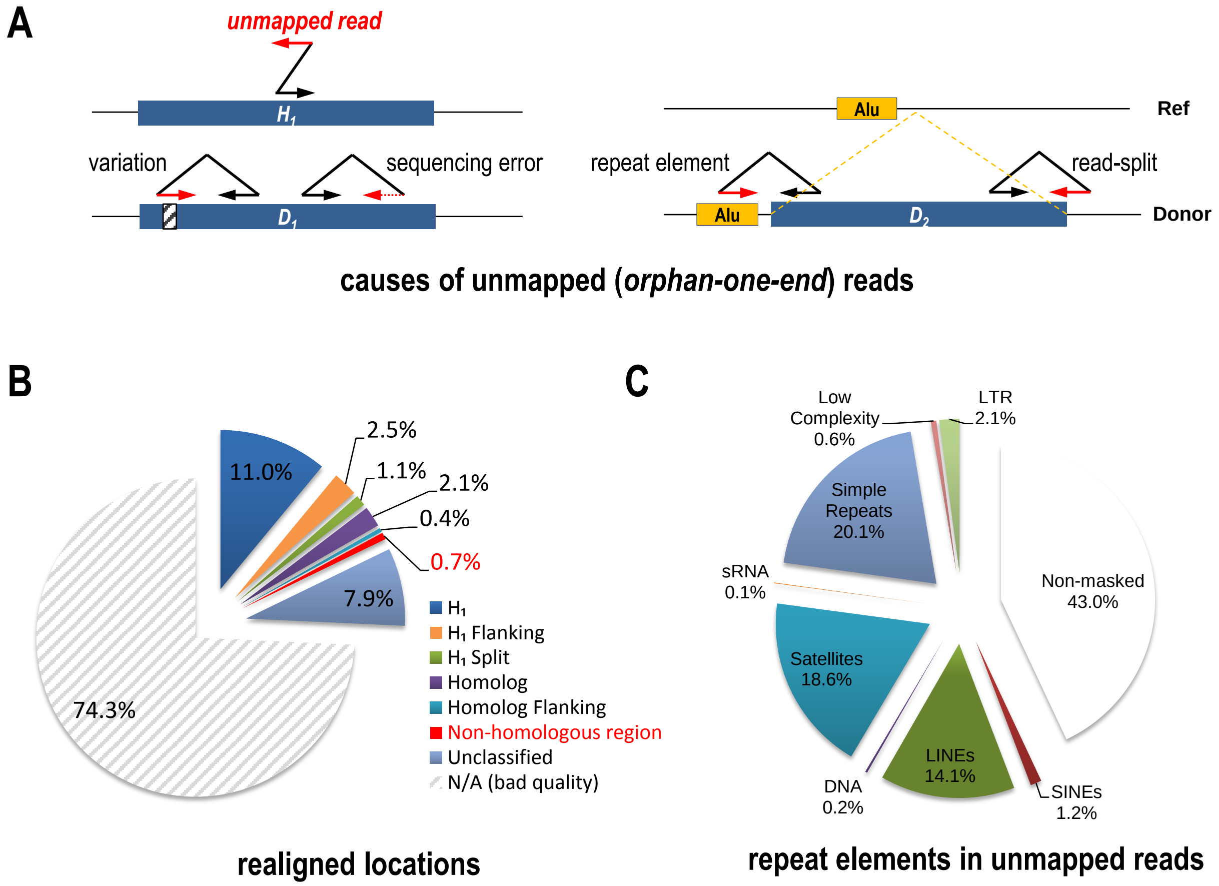

Figure S5: Classification of paired end mapping with respect to a high copy region H. (a) A *Two-end* read is a paired end read that is successfully mapped inside H. It can be from the same region (blue) or any duplicates (red). (b) When only one read is mapped to H and the mapping is concordant, it is classified as *concordant-one-end* read. The read mapped inside H is *internal* (the right read of blue and the left read of red), while the other is *external*. (c) When the *one-end* read is discordant (by insert size or coordinate), it is classified as *discordant-one-end* read. It can be generated from boundaries of duplicates. Discordant one-end reads play an important role because its external reads can be used to infer breakpoints while the internal reads provide variation information in the duplicate boundaries. (d) When the external read is not mapped (orphan), the paired end read is called *orphan-one-end* read. It can be generated from excessive variation inside duplicates (blue), repeat elements in flanking regions (magenta), or split-read generation at the boundaries (red).

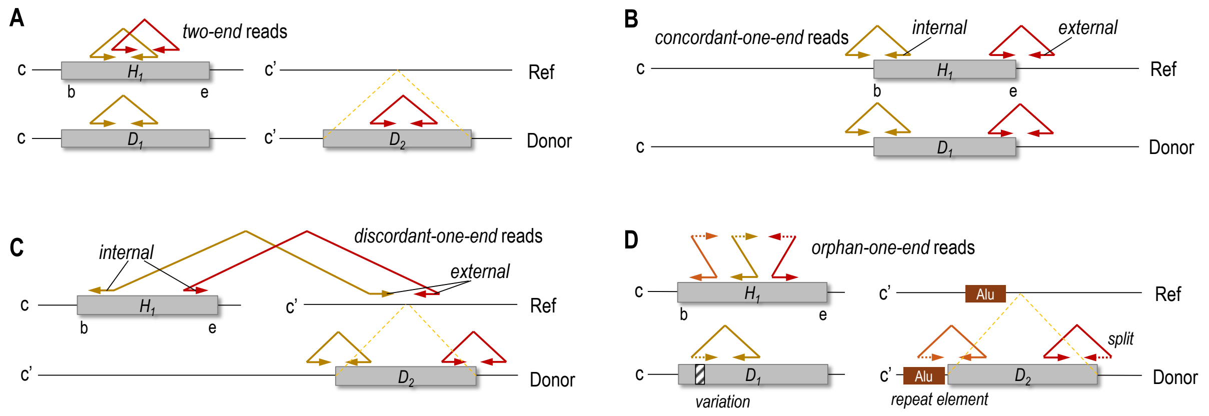

Figure S6: A landscape of copy number increase in NA18507. Totally 990 duplication events whose insertion locations were identifiable are drawn (center circle). Each edge connects a original copy location to its corresponding insertion location; edge color corresponds to the original site. We found no specific preference in chromosomes or chromosomal locations for duplication site. Five sub-networks are also drawn including stronger candidates (top), inter/intra-chromosome duplication (right up and down), and homo/heterozygous duplication (left up and down). Networks are drawn using Circos (Krzywinski et al., 2009).

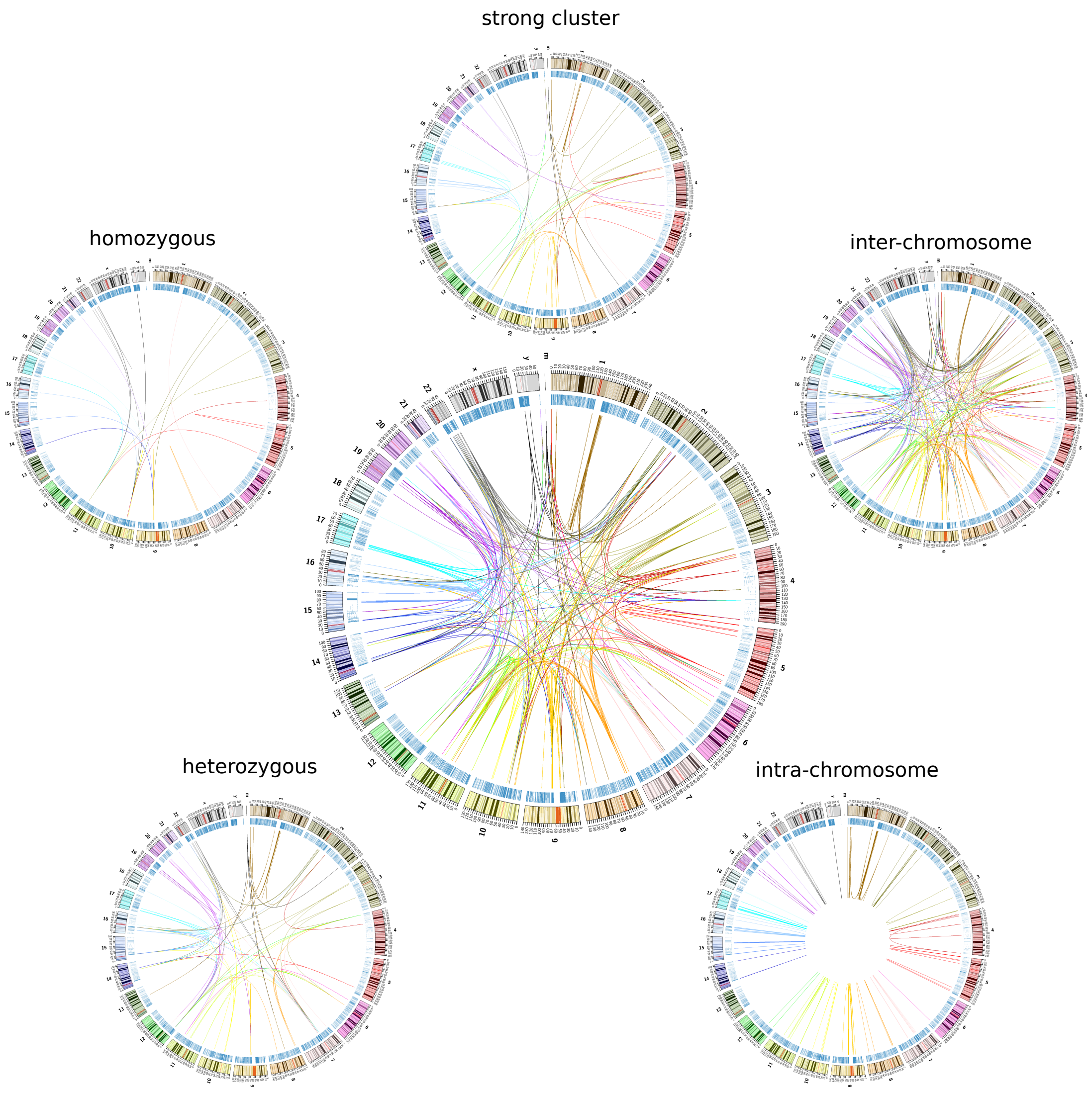

Figure S7: Complex copy number variation in *MUC20* explained by discordant reads. A. Many alternative transcripts are found in *MUC20* around the exon 2 and 3. Short tandem repeats are shown in the intron region. B. Discordant-one-end reads from REPREVER$_{LOC}$ (red and blue solid arrows) represent a breakpoint near the intron 2. Non-unique sequences caused from segmental duplication provide a secondary mapping place for each read (lined arrows), however, mate-pairs remain discordant due to the abnormally large insert size. C. A non allelic homologous recombination (NAHR) can occur between two short tandem repeats (yellow dashed line). A few repeat units also can be omitted during the recombination. D. The NAHR explains and resolves all the discordance: i) the gain call made by CNVer, ii) increased insert size of mate-pairs, iii) discordant mapping across the two homologs predicted from breakpoint specificity.

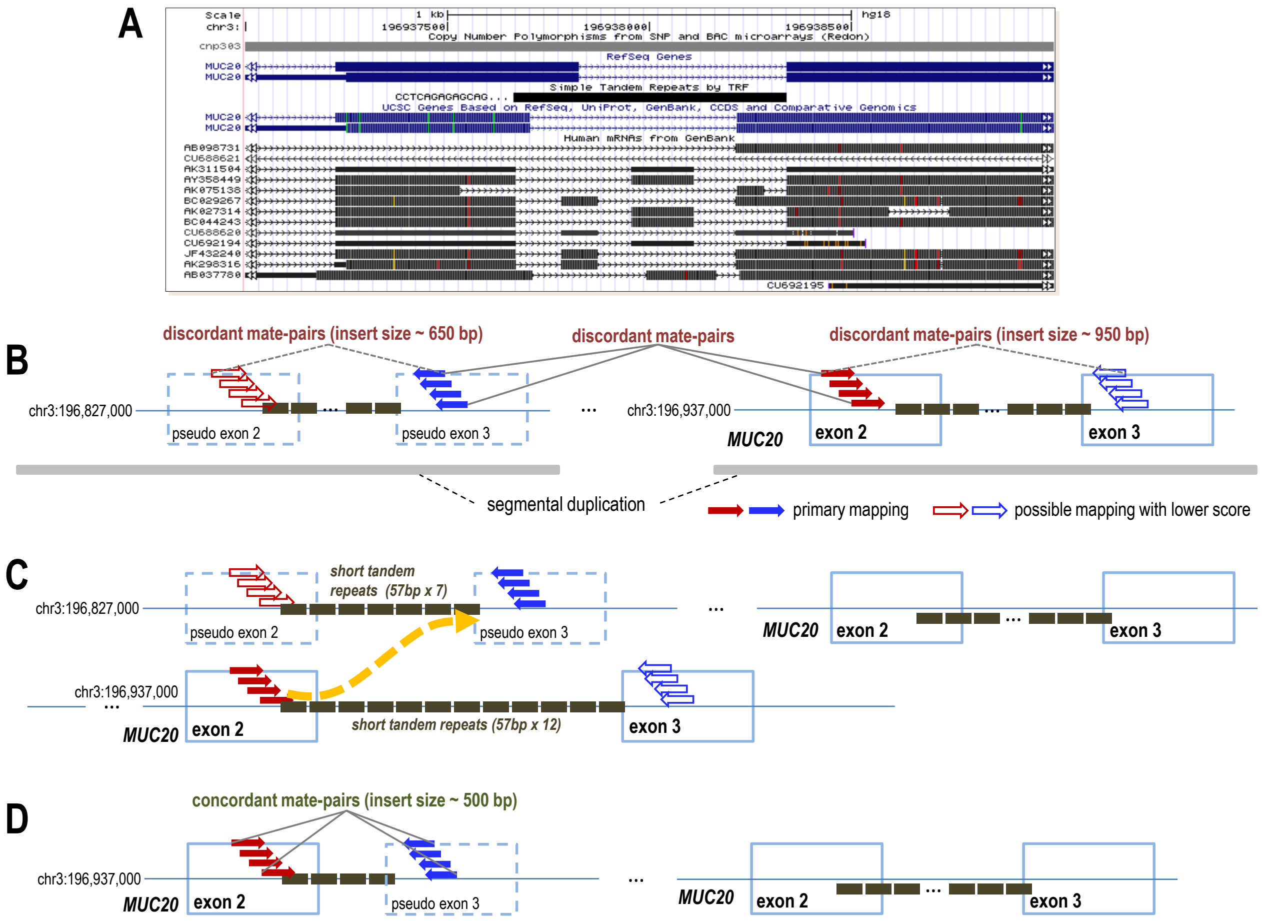

Figure S8: With an significant sized insertion, mate-pairs generated across the breakpoint in the donor genome are not mapped *concordantly* in the reference genome. Left: There is no change of the breakpoint coverage when no duplication is occurred. Middle: Mate-pairs generated in or across the inserted block D (red lines) are not mapped in the reference causing roughly 50% reduction of coverage at the breakpoint. Right: Likewise, we can observe a "V" shaped reduction of coverage around the homozygous breakpoint.

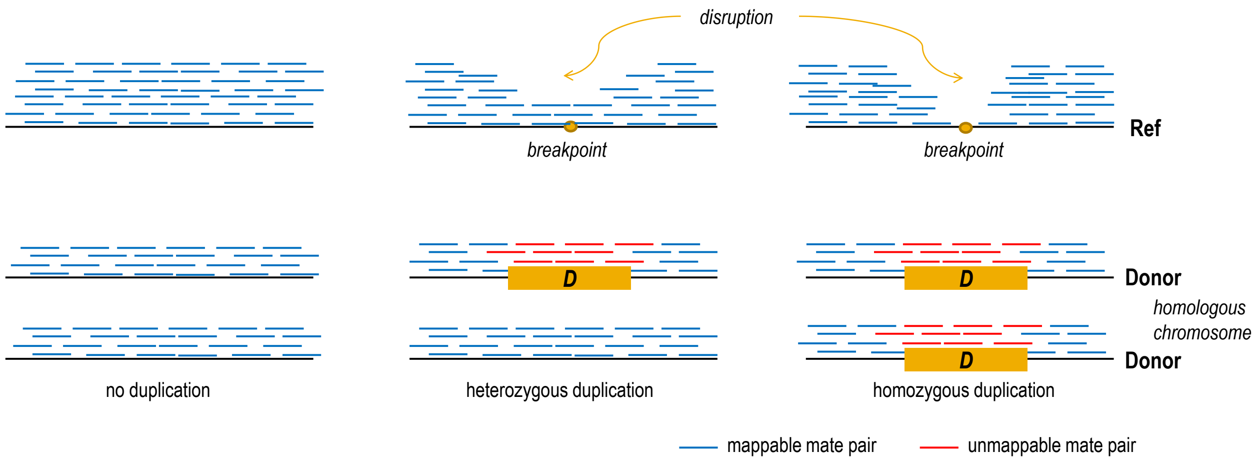

Figure S9: An overview of duplicate identification process. (a) Boundary regions of duplicates can be reconstructed from discordant-one-end reads where one (*external*) sub-read is mapped to the flanking region of breakpoints and the other sub-read (*internal*) is mapped to the boundary regions of the duplicates (D_1 to D_N). If there are any variations including SNPs and micro-indels in the boundaries (colored narrow boxes), we can differentiate the duplicates by training PHMMs using assigned reads (right). (b) After training boundary regions, two-end reads are used for reconstruction of central regions. For each two-end read, one sub-read is used to determining where it is from (e.g. the one in the figure is assigned to D_N) and the other end is used for extending trained regions (dotted ovals below each duplicate). This is an iterative process and is repeated until 1) all the reads are assigned to any duplicates or 2) when trained regions cannot be extended.

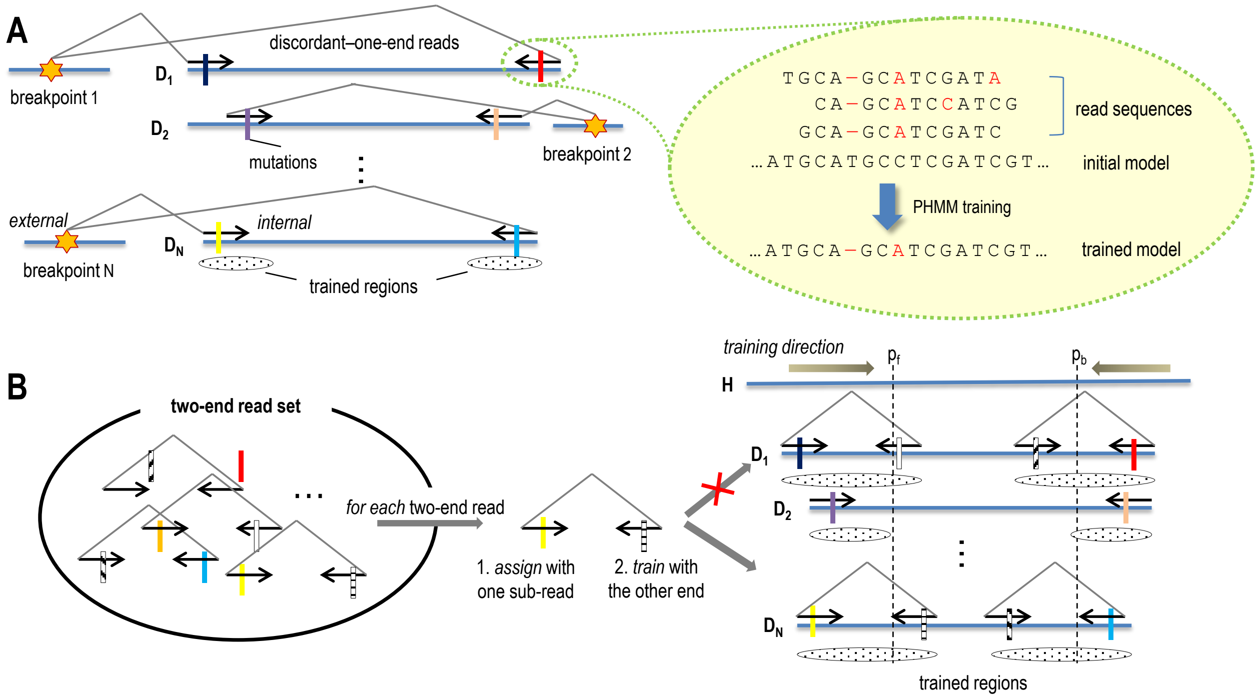

Figure S10: Getting a template sequence of a duplicate (D_2) from the original copy (H). In case of the duplicate is truncated when it is first generated, a subsequence of H is used as the template. The range of subsequence can be inferred from internal sub-reads; the new start point (b') is given by the leftmost position of all reverse internal sub-reads (red), and the new end point (e') is by the rightmost position of all forward internal sub-reads (green).

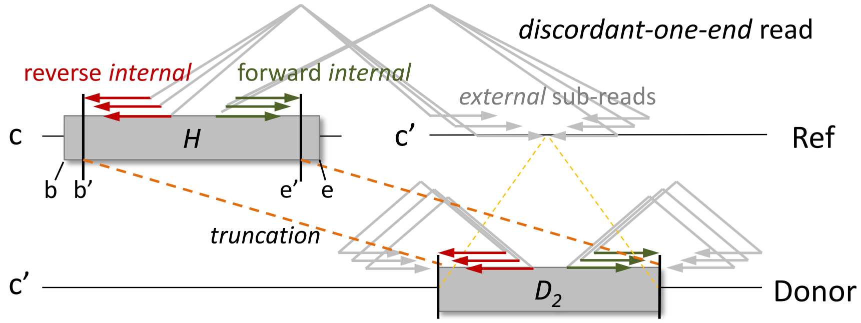

Figure S11: An example profile HMM for sequence modeling. A PHMM can model a nucleotide sequence by updating probabilistic parameters. A series of observation changes parameters of match states (M_1 to M_i) to determine the most probable alphabet (nucleotide) at each state. Observed insertion or deletion can changes transition parameter. The most probable sequence (T-AAC/G) is generated from state changes (red lines) and values of emission functions (read numbers).

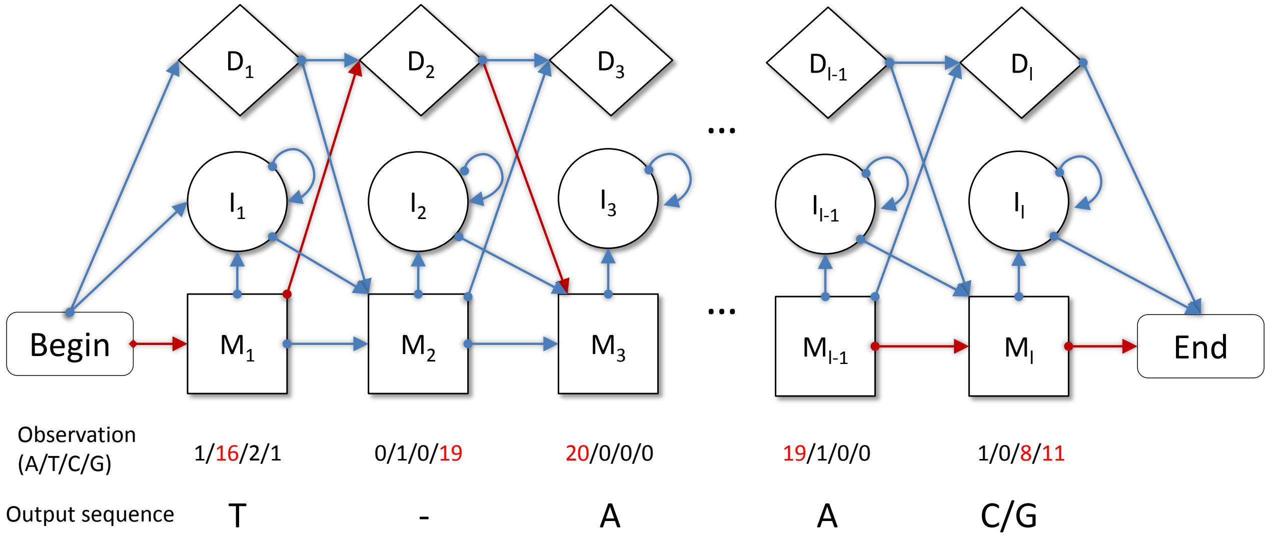

Figure S12: PCR experiments on high confident NA18507 duplicons. Out of 10 total regions, six regions not presented in the main manuscript are shown. From the 12 primer pairs, we successfully confirmed the amplification at 8 pairs (21, 22, 24, 25, 26, 27, 28, 29). Detailed primer information is available in Table S4.

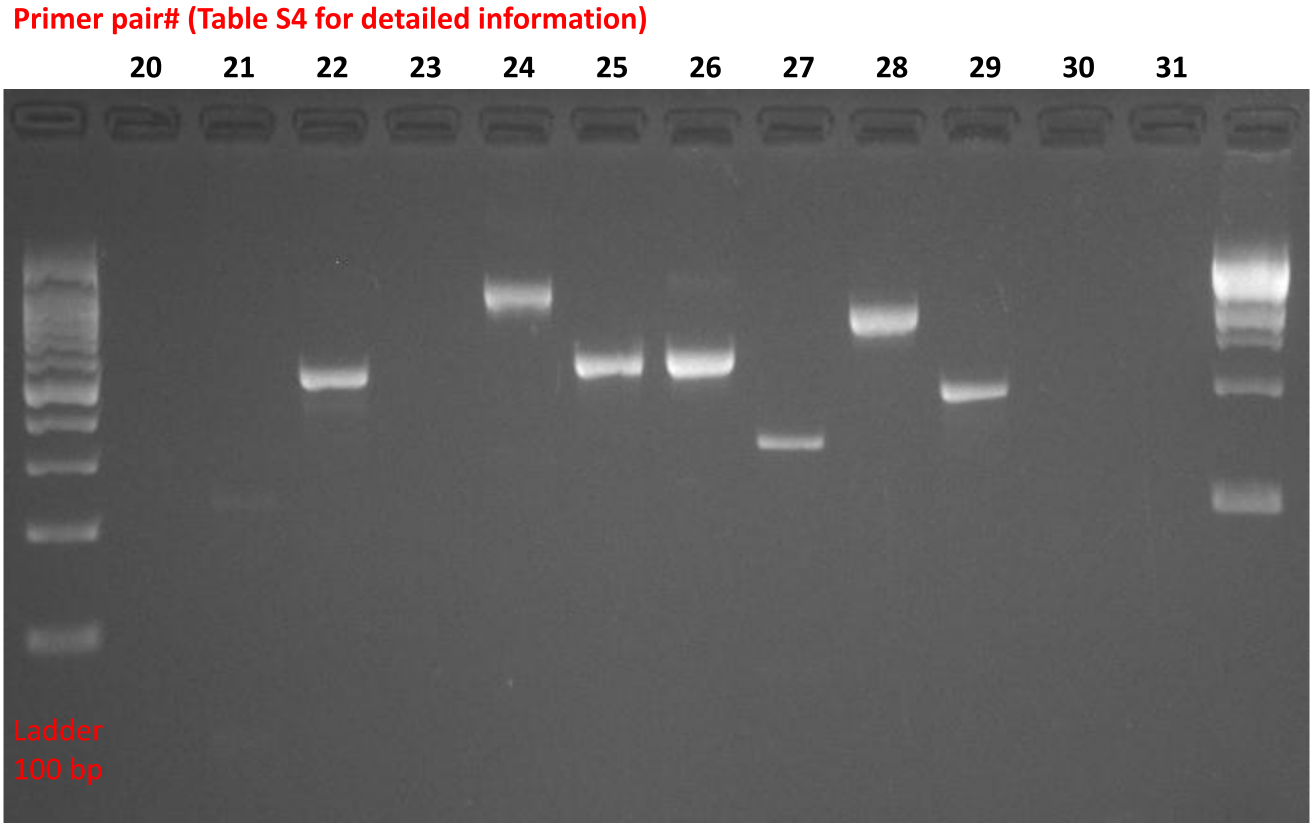

2 Supplementary Tables

Table S1: The method for Insertion breakpoint varies widely depending on the size and contents of the inserted sequences. For example, small insertions ($\sim$a few bp) can be found from mismatches in alignment without considering any discordant mapping coordinates. Insertions of larger ($>$ 1kb) sequences exist in fewer numbers and harder to be identified. There have been a few trials to reconstruct inserted sequences using local assembly around the breakpoint. However, most of them are targeted to novel sequences (which do not exist in the reference sequence). Insertion of duplicated sequences (exist in reference sequence in one or many copies) has been rarely validated either by computational or experimental ways. [a] Inserted sequence or its homologs exist in the reference. Here we define homologs as sequences whose length is within 90% 110% of the original sequence, and >95% sequence identity. [b] The number of assembled sequences are dependent on the 1) tools that are used in assembly (e.g. Velvet, Euler) and 2) stringency for contig assembly. [c] we additionally found Blat matches from 777/2898 inserted sequences, which are not analyzed in the original study and yet to be validated.

		Insertion breakpoint		Inserted sequence		
Study	Method	all	long ($>$ 1kb)	all	duplicated[a]	Reference
Bentley *et al*	read pair + local assembly	5704	61	2345	*NA*	(Bentley et al., 2008)
McKernan *et al*	read pair	1515	$<$ 10	*NA*	*NA*	(McKernan et al., 2009)
Ye *et al*	split-read	142908	*NA*	*NA*	*NA*	(Ye et al., 2009)
Harijasouliha *et al*	read pair + local assembly	4115	778	78 to 130[b]	*NA*	(Hajirasouliha et al., 2010)
Kidd *et al*	fosmid sequencing	101	101	4	0	(Kidd et al., 2008)
Li *et al*	whole genome assembly	87457	2898	2898	*NA*[c]	(Li et al., 2011)

Table S2: Accuracy of Reprever reconstructed sequences of fosmids. Sequences are reconstructed by REPREVER from the original form (ref to ins.Reprever). Compared with the true sequence identified by full fosmid sequencing, the REPREVER constructed sequences show much high similarity (in the first two duplicons). While the reconstruction separates two or more sequences by their variations, some repeat elements (e.g. LINEs) or identical sequences (chrX:26720703) are hardly differentiated. For those six trivial repeat element duplications, we are listing three as examples. ins.Reprever: Reprever reconstructed sequences. Following numbers are used to separate multiple duplicated regions. ref: reference sequences. Following numbers are used to separate multiple homologs in the reference. len.match: alignment length, n.mismatch: number of mismatches. n.gap.open: number of gap openings, seq.sim: sequence similarity in percent, bit.score: bit-score from blast2

Duplication site	Sequence Comparison	len.match	n.mismatch	n.gap.open	seq.sim (%)	bit.score
chr3:99355872-99357168	ins.Reprever↔ fosmid	1267	21	3	**97.87**	2300
	ref↔ fosmid	1296	103	4	91.13	1641
chr1:16729161-16736531	ins.Reprever↔ fosmid	5559	68	7	**98.35**	10100
	ref1↔ fosmid	5572	183	13	96.16	9028
	ref2↔ fosmid	5570	179	12	96.37	8990
chr4:137434000-137441000 (LINE)	ins.Reprever1↔ fosmid	2791	6	0	**99.79**	5313
	ins.Reprever2↔ fosmid	2841	6	0	99.79	5414
	ref1↔ fosmid	2841	6	0	99.79	5412
chrX:26720703-26731669	ins.Reprever↔ fosmid	4851	0	0	**100.0**	9141
	ref↔ fosmid	4851	0	0	100.0	9141
chr6:56866310-56869100	ins.Reprever↔ fosmid	957	15	0	**98.43**	1680
	ref↔ fosmid	957	15	0	98.43	1677

Table S3. All predicted breakpoints in gene region. CDS: coding DNA sequence. near-gene-3/near-gene-5: intergenic region but genes are near at 5' or 3', utr-5/utr-3: 5' or 3' untranslated region.

Breakpoint	Region	Gene	Score	Duplicated from	CNV source
chr1:80400	near-gene-3	none	4.29	chr1:226555-227613	CNVer
chr1:230488	intergenic	none	2.92	chr1:241242611-241243715	CNVer
chr1:243425	intergenic	none	3.41	chr1:241255523-241256807	CNVer
chr1:394135	intergenic	none	4.44	chr1:222174594-222177176	CNVer
chr1:523668	intergenic	none	47.31	chr5:180809672-180811063	CNVer
chr1:544697	intergenic	none	4.67	chr1:544284-545519	CNVer
chr1:561724	intergenic	none	2.54	chr1:407734-408951	CNVer
chr1:576435	intergenic	none	4.65	chr1:393017-394466	CNVer
chr1:581711	intergenic	none	2.85	chr1:581601-582500	Yoon
chr1:2599616	intergenic	none	3.09	chr1:2675149-2681655	CNVer
chr1:12830861	utr-5	HNRNPCL1	10.5	chr1:12865901-12869700	Yoon
chr1:12830861	utr-5	HNRNPCL1	5.0	chr1:12812801-12815300	Yoon
chr1:12928207	intron	PRAMEF6	7.6	chr1:13515701-13518000	Yoon
chr1:13038539	intron	PRAMEF23	6.6	chr1:13515701-13518000	Yoon
chr1:13233395	intron	PRAMEF5	6.6	chr1:13515701-13518000	Yoon
chr1:13237077	intron	PRAMEF5	7.84	chr1:13296001-13297200	Yoon
chr1:13296337	intron	PRAMEF9	8.91	chr1:13296001-13297200	Yoon
chr1:13517134	intron	PRAMEF15	8.91	chr1:13296001-13297200	Yoon
chr1:16706168	intergenic	none	2.38	chr1:17101501-17107700	Yoon
chr1:16711652	intergenic	none	48.26	chr1:16940845-16941986	CNVer
chr1:16728070	intergenic	none	3.41	chr1:16869002-16870352	CNVer
chr1:16740890	intergenic	none	3.27	chr1:16739901-16741500	Yoon
chr1:16766464	intron	NBPF1	62.07	chr1:146666279-146668660	CNVer
chr1:16775357	CDS	NBPF1	33.02	chr1:144764979-144766184	CNVer
chr1:16778388	CDS	NBPF1	100.07	chr1:143331886-143333077	CNVer
chr1:16782962	intron	NBPF1	5.5	chr1:143326974-143328066	CNVer
chr1:16784684	CDS	NBPF1	37.8	chr1:143331886-143333077	CNVer
chr1:16787450	intron	NBPF1	13.44	chr1:144764979-144766184	CNVer
chr1:16789978	intron	NBPF1	12.63	chr1:143326974-143328066	CNVer
chr1:16790731	intron	NBPF1	7.72	chr1:143331886-143333077	CNVer
chr1:16792907	intron	NBPF1	105.0	chr1:21665442-21666464	CNVer
chr1:16827144	intergenic	none	35.0	chr1:17138815-17144035	CNVer
chr1:16849368	intergenic	none	32.67	chr1:16954883-16956219	CNVer
chr1:16854111	intergenic	none	4.0	chr1:16946002-16952712	CNVer
chr1:16854111	intergenic	none	2.5	chr1:16944901-16955500	Yoon
chr1:16858715	intergenic	none	4.27	chr1:17101501-17107700	Yoon
chr1:16859425	intergenic	none	19.97	chr1:17100686-17102047	CNVer
chr1:16864326	intergenic	none	11.81	chr1:16940845-16941986	CNVer
chr1:16864684	intergenic	none	6.34	chr1:17095777-17096866	CNVer
chr1:16870173	near-gene-5	none	13.15	chr1:17076474-17077687	CNVer
chr1:16870594	near-gene-5	none	11.5	chr1:16705701-16716100	Yoon
chr1:16934003	near-gene-5	none	11.5	chr1:16705701-16716100	Yoon
chr1:16935082	near-gene-5	none	13.15	chr1:17076474-17077687	CNVer
chr1:16940120	intergenic	none	6.34	chr1:17095777-17096866	CNVer
chr1:16941013	intergenic	none	10.31	chr1:16940845-16941986	CNVer
chr1:16946184	intergenic	none	5.27	chr1:17101501-17107700	Yoon
chr1:16955056	intergenic	none	19.6	chr1:16944901-16955500	Yoon
chr1:16955621	intergenic	none	10.14	chr1:16954883-16956219	CNVer
chr1:17049075	intergenic	none	2.67	chr1:17048249-17049434	CNVer
chr1:17055322	intergenic	none	2.71	chr1:21610608-21611619	CNVer
chr1:17061704	intergenic	none	2.0	chr1:147551384-147562101	CNVer
chr1:17074390	intergenic	none	3.0	chr1:17067701-17079000	Yoon
chr1:17078417	intergenic	none	5.92	chr1:17078749-17093217	Conrad
chr1:17096425	intergenic	none	8.14	chr1:16940845-16941986	CNVer
chr1:17101863	intergenic	none	3.5	chr1:16946002-16952712	CNVer
chr1:17128098	intron	CROCC	4.91	chr1:16946002-16952712	CNVer
chr1:17128098	intron	CROCC	4.91	chr1:16944901-16955500	Yoon
chr1:21665540	intron	NBPF3	38.91	chr1:21665442-21666464	CNVer
chr1:24410609	intergenic	none	4.91	chr1:24411201-24411600	Yoon
chr1:104057613	intergenic	none	3.34	chr1:103963451-103964799	CNVer
chr1:116997376	intron	IGSF3	3.0	chr22:19817056-19833887	CNVer
chr1:120483042	intergenic	none	3.57	chr1:120480951-120483801	CNVer
chr1:120557986	intergenic	none	3.0	chr1:120554067-120569504	CNVer
chr1:120603456	intergenic	none	3.34	chr1:120603310-120604637	CNVer
chr1:120654135	intron	"FAM72B,LOC100132913"	2.54	chr1:204317082-204319528	CNVer
chr1:120841417	intergenic	none	2.0	chr16:33758958-33762607	CNVer
chr1:120843028	intergenic	none	4.88	chr7:64653493-64654919	CNVer
chr1:121053201	intergenic	none	35.19	chr17:22177077-22178361	CNVer
chr1:121053201	intergenic	none	14.32	chr17:22179452-22180782	CNVer
chr1:121173746	intergenic	none	2.26	chr1:121078438-121079461	CNVer
chr1:121177171	intergenic	none	3.91	chr1:121125844-121127373	CNVer
chr1:121186679	intergenic	none	8.38	chr4:67946975-67949285	CNVer
chr1:141495450	intergenic	none	6.31	chr3:75981170-75999076	CNVer
chr1:141598054	intergenic	none	6.87	chr1:141991684-141995184	CNVer
chr1:141897117	intergenic	none	5.81	chr3:75981170-75999076	CNVer
chr1:141973422	intergenic	none	7.0	chr4:49306569-49320341	CNVer
chr1:141997763	intergenic	none	5.57	chr1:142025320-142026647	CNVer
chr1:142026368	intergenic	none	6.39	chr1:141996906-142000118	CNVer
chr1:142109986	intron	LOC100132733	8.03	chr9:45243942-45250716	CNVer
chr1:142118572	intergenic	none	8.14	chr4:49213211-49214569	CNVer
chr1:142125160	intergenic	none	7.05	chr4:49017970-49020759	CNVer
chr1:142188934	intergenic	none	3.5	chr1:142081233-142082456	CNVer
chr1:142370694	intergenic	none	11.48	chr3:75981170-75999076	CNVer

chr1:142436568	intergenic	none	3.84	chr1:143278470-143284266	CNVer
chr1:142468300	intergenic	none	4.0	chr1:17090756-17092275	CNVer
chr1:142545063	intergenic	none	18.34	chr1:142545001-142546700	Yoon
chr1:142552050	intergenic	none	2.29	chr1:146558401-146559100	Yoon
chr1:142619075	intron	FAM72D	3.94	chr1:204307346-204311980	CNVer
chr1:142641181	intron	LOC100133095	4.53	chr1:204283629-204291142	CNVer
chr1:142641181	intron	LOC100133095	4.53	chr1:142639574-142642992	CNVer
chr1:142689404	intron	LOC100133095	2.79	chr1:120574865-120585820	CNVer
chr1:142689404	intron	LOC100133095	2.79	chr1:204239481-204240785	CNVer
chr1:142779566	intergenic	none	14.0	chr1:142779610-142780768	CNVer
chr1:142805526	intergenic	none	2.59	chr1:142805391-142806746	CNVer
chr1:142858703	intron	RP3-377D14.1	7.96	chr1:143326974-143328066	CNVer
chr1:142860757	intron	RP3-377D14.1	10.99	chr1:144764979-144766184	CNVer
chr1:142884448	intron	RP3-377D14.1	2.6	chr1:144070468-144072513	CNVer
chr1:143013297	intergenic	none	3.67	chr1:17082591-17090134	CNVer
chr1:143014095	intergenic	none	5.71	chr1:17078749-17093217	Conrad
chr1:143193526	intergenic	none	3.67	chr1:17082591-17090134	CNVer
chr1:143194324	intergenic	none	5.71	chr1:17078749-17093217	Conrad
chr1:143316917	intergenic	none	8.0	chr1:146720981-146722375	CNVer
chr1:143327405	intergenic	none	19.08	chr1:143326974-143328066	CNVer
chr1:143330263	intergenic	none	32.08	chr1:146489592-146490634	CNVer
chr1:143387905	utr-3	LOC653513	2.43	chr1:143386293-143389738	CNVer
chr1:143393224	near-gene-5	none	2.5	chr1:143393044-143397164	CNVer
chr1:143395789	intergenic	none	2.0	chr1:143393044-143397164	CNVer
chr1:143523011	intergenic	none	8.68	chr1:143331886-143333077	CNVer
chr1:143524449	intergenic	none	24.91	chr1:144760444-144762311	CNVer
chr1:143525706	intergenic	none	10.2	chr1:144764979-144766184	CNVer
chr1:143534661	intergenic	none	2.16	chr1:146666279-146668660	CNVer
chr1:143539451	intergenic	none	19.35	chr1:146661361-146662377	CNVer
chr1:143539513	intergenic	none	19.81	chr1:142902854-142903931	CNVer
chr1:143548193	intergenic	none	24.32	chr1:144087650-144088981	CNVer
chr1:143553241	intergenic	none	33.72	chr1:144945273-144953111	CNVer
chr1:143663002	intron	PDE4DIP	2.53	chr1:143386293-143389738	CNVer
chr1:143665870	intron	PDE4DIP	39.2	chr1:143665501-143669098	CNVer
chr1:143668190	intron	PDE4DIP	2.5	chr1:143393044-143397164	CNVer
chr1:143671110	intron	PDE4DIP	2.0	chr1:143393044-143397164	CNVer
chr1:143897939	intergenic	none	2.34	chr1:120432333-120439967	CNVer
chr1:143918883	near-gene-5	none	3.8	chr1:120414897-120417723	CNVer
chr1:143989906	intron	NOTCH2NL	25.3	chr1:120333775-120344306	CNVer
chr1:144005415	intron	LOC100132406	10.16	chr1:143326974-143328066	CNVer
chr1:144008986	CDS	LOC100132406	43.25	chr1:146489592-146490634	CNVer
chr1:144008986	CDS	LOC100132406	4.12	chr1:144015301-144022900	Yoon
chr1:144011688	intron	LOC100132406	24.3	chr1:143326974-143328066	CNVer
chr1:144013697	intron	LOC100132406	27.01	chr1:144764979-144766184	CNVer
chr1:144014865	intron	LOC100132406	5.65	chr1:146489592-146490634	CNVer
chr1:144079476	intergenic	none	11.83	chr1:142902854-142903931	CNVer
chr1:144079476	intergenic	none	11.49	chr1:146661361-146662377	CNVer
chr1:144088065	intergenic	none	13.89	chr1:144087650-144088981	CNVer
chr1:144745902	intron	NBPF11	5.9	chr1:142902854-142903931	CNVer
chr1:144745902	intron	NBPF11	5.9	chr1:146661361-146662377	CNVer
chr1:144750500	CDS	NBPF11	2.42	chr1:146666279-146668660	CNVer
chr1:144757590	intron	NBPF11	11.16	chr1:146489592-146490634	CNVer
chr1:144764035	CDS	NBPF11	9.23	chr1:146489592-146490634	CNVer
chr1:144764035	CDS	NBPF11	2.09	chr1:144015301-144022900	Yoon
chr1:144774966	intron	NBPF11	7.92	chr1:146836481-146838038	CNVer
chr1:144927199	intron	NBPF12	6.3	chr1:146661361-146662377	CNVer
chr1:144929098	intron	NBPF12	9.64	chr1:146661361-146662377	CNVer
chr1:144940776	intergenic	none	20.81	chr1:144087650-144088981	CNVer
chr1:145050812	intergenic	none	6.0	chr1:142870650-142873029	CNVer
chr1:145050839	intergenic	none	4.0	chr1:146471501-146476000	Yoon
chr1:145050887	intergenic	none	6.0	chr1:144040239-144045077	CNVer
chr1:145053143	intergenic	none	2.32	chr1:144021355-144022844	CNVer
chr1:145053143	intergenic	none	2.15	chr1:143532087-143534135	CNVer
chr1:145053143	intergenic	none	2.32	chr1:144015301-144022900	Yoon
chr1:146039999	intergenic	none	2.08	chr1:144082083-144083808	CNVer
chr1:146042950	intron	XXyac-YX155B6.1	6.74	chr1:142902854-142903931	CNVer
chr1:146042950	intron	XXyac-YX155B6.1	6.74	chr1:146661361-146662377	CNVer
chr1:146054643	intron	XXyac-YX155B6.1	11.16	chr1:146489592-146490634	CNVer
chr1:146061088	CDS	XXyac-YX155B6.1	10.15	chr1:146489592-146490634	CNVer
chr1:146061088	CDS	XXyac-YX155B6.1	3.01	chr1:144015301-144022900	Yoon
chr1:146072069	intron	XXyac-YX155B6.1	7.92	chr1:146836481-146838038	CNVer
chr1:146299600	intergenic	none	25.84	chr1:143278470-143284266	CNVer
chr1:146330914	intergenic	none	4.0	chr1:17090756-17092275	CNVer
chr1:146491333	splice-5	NBPF14	19.76	chr1:144764979-144766184	CNVer
chr1:146619176	intron	NBPF20	3.74	chr1:142902854-142903931	CNVer
chr1:146619176	intron	NBPF20	3.74	chr1:146661361-146662377	CNVer
chr1:146703940	splice-5	NBPF20	10.95	chr1:144764979-144766184	CNVer
chr1:146705496	intron	NBPF20	7.96	chr1:143326974-143328066	CNVer
chr1:146707160	intron	NBPF20	7.91	chr1:143331886-143333077	CNVer
chr1:146721044	intergenic	none	6.0	chr1:146720981-146722375	CNVer
chr1:146811342	intergenic	none	5.0	chr1:146802488-146812680	CNVer
chr1:146834534	intron	NBPF15	5.25	chr1:146720981-146722375	CNVer
chr1:146847193	CDS	NBPF15	19.41	chr1:144764979-144766184	CNVer
chr1:146860489	intron	NBPF15	4.08	chr1:142902854-142903931	CNVer
chr1:146860489	intron	NBPF15	3.7	chr1:146661361-146662377	CNVer
chr1:146867083	intergenic	none	2.0	chr1:146610778-146613102	CNVer
chr1:146924259	intergenic	none	2.61	chr1:142545001-142546700	Yoon
chr1:146928574	intergenic	none	5.71	chr7:64204600-64205715	CNVer
chr1:146928996	intergenic	none	2.02	chr7:64687738-64692238	CNVer

chr1:146929030	intergenic	none	7.64	chr16:33742239-33743375	CNVer
chr1:146934622	intergenic	none	2.2	chr7:61411792-61425541	CNVer
chr1:146937256	intergenic	none	2.74	chr16:32012045-32013573	CNVer
chr1:147005950	near-gene-5	none	3.05	chr1:143331886-143333077	CNVer
chr1:147007541	intron	NBPF16	9.22	chr1:144760444-144762311	CNVer
chr1:147009280	CDS	NBPF16	22.42	chr1:144764979-144766184	CNVer
chr1:147022533	intron	NBPF16	6.42	chr1:142902854-142903931	CNVer
chr1:147022533	intron	NBPF16	6.04	chr1:146661361-146662377	CNVer
chr1:147090027	intergenic	none	5.18	chr1:147089712-147090923	CNVer
chr1:147090652	intergenic	none	3.02	chr7:64204600-64205715	CNVer
chr1:147090941	intergenic	none	4.99	chr16:33742239-33743375	CNVer
chr1:147099166	intergenic	none	3.74	chr16:32012045-32013573	CNVer
chr1:147109554	intergenic	none	4.34	chr1:146929658-146949571	CNVer
chr1:147110284	near-gene-3	none	4.06	chr7:57928826-57935924	CNVer
chr1:147113469	intron	LOC645146	4.93	chr16:32040890-32058514	CNVer
chr1:147113877	intron	LOC645146	9.54	chr7:64649866-64652666	CNVer
chr1:147117708	intron	LOC645146	4.75	chr7:64653493-64654919	CNVer
chr1:147118875	intron	LOC645146	2.0	chr16:33758958-33762607	CNVer
chr1:147119217	intron	LOC645146	5.69	chr16:32943979-32947198	CNVer
chr1:147290289	intergenic	none	27.62	chr1:147089712-147090923	CNVer
chr1:147296943	intergenic	none	5.93	chr16:32968567-32977402	CNVer
chr1:147297379	intergenic	none	5.3	chr7:64211541-64213222	CNVer
chr1:147393906	intergenic	none	2.0	chr1:146725998-146727456	CNVer
chr1:147487210	intergenic	none	28.47	chr1:16705701-16716100	Yoon
chr1:147492113	intergenic	none	3.39	chr1:16705701-16716100	Yoon
chr1:147528785	near-gene-3	none	2.65	chr1:147913129-147918258	CNVer
chr1:147561632	intron	LOC100131974	2.89	chr1:147551384-147562101	CNVer
chr1:147606097	intergenic	none	2.34	chr1:147990883-147994800	CNVer
chr1:147607398	intergenic	none	2.0	chr1:147990883-147994800	CNVer
chr1:147607464	intergenic	none	3.0	chr1:147607648-147610104	CNVer
chr1:147881316	intergenic	none	4.67	chr1:17082591-17090134	CNVer
chr1:147912364	intergenic	none	2.0	chr1:147526838-147529189	CNVer
chr1:147916670	intron	LOC729130	2.34	chr1:147913129-147918258	CNVer
chr1:147927270	intron	LOC729130	4.35	chr1:147926825-147929569	CNVer
chr1:147965460	intron	LOC729130	30.57	chr1:147578926-147581084	CNVer
chr1:148002883	intergenic	none	3.03	chr1:148001298-148003381	CNVer
chr1:148010043	intergenic	none	3.0	chr1:148008659-148020018	CNVer
chr1:150543274	intron	”FLG,LOC100128468”	10.84	chr1:150546324-150548270	CNVer
chr1:204297963	near-gene-5	none	8.06	chr1:120632932-120634987	CNVer
chr1:204623799	intron	SRGAP2	12.45	chr1:142805391-142806746	CNVer
chr1:204626373	intron	SRGAP2	2.91	chr1:120809705-120811788	CNVer
chr1:204636666	intron	SRGAP2	3.0	chr1:120820509-120827073	CNVer
chr1:222165603	intergenic	none	9.05	chr1:402839-404008	CNVer
chr1:222178167	intergenic	none	6.15	chr1:241255523-241256807	CNVer
chr1:241245899	intergenic	none	5.38	chr1:567363-568977	CNVer
chr1:241245899	intergenic	none	2.21	chr1:222166885-222168016	CNVer
chr1:241255837	intergenic	none	2.71	chr1:241255523-241256807	CNVer
chr2:72540475	intron	EXOC6B	2.8	chr11:93606508-93612450	CNVer
chr2:87460602	CDS	LOC730259	2.4	chr16:33232577-33234387	CNVer
chr2:88069816	intergenic	none	3.34	chr2:86983834-86985421	CNVer
chr2:88855856	intergenic	none	56.4	chr2:88855580-88856634	CNVer
chr2:88858976	intergenic	none	4.0	chr2:88857969-88859821	CNVer
chr2:88861389	intergenic	none	18.82	chr2:88860743-88862181	CNVer
chr2:88862914	intergenic	none	7.7	chr2:97207601-97209992	CNVer
chr2:88863829	intergenic	none	5.91	chr2:97217539-97218730	CNVer
chr2:89931954	intergenic	none	6.48	chr9:67044163-67045630	CNVer
chr2:90985126	intergenic	none	77.84	chr16:32960738-32963917	CNVer
chr2:90989632	intergenic	none	4.33	chr16:32988816-33003467	CNVer
chr2:90989632	intergenic	none	4.29	chr1:146929658-146949571	CNVer
chr2:90991545	intergenic	none	31.22	chr9:69462514-69465468	CNVer
chr2:90994957	intergenic	none	130.06	chr16:32950741-32951758	CNVer
chr2:91002329	intron	LOC339562	6.48	chr7:64653493-64654919	CNVer
chr2:91005208	intron	LOC339562	8.38	chr7:61464579-61475813	CNVer
chr2:91009760	intron	LOC339562	2.06	chr7:61464579-61475813	CNVer
chr2:91019983	intron	LOC339562	7.33	chr16:32960738-32963917	CNVer
chr2:91031689	intron	LOC339562	2.24	chr16:33758958-33762607	CNVer
chr2:91043220	utr-3	LOC339562	4.6	chr9:69425990-69433984	CNVer
chr2:91085367	near-gene-5	none	3.06	chr15:19597380-19613724	CNVer
chr2:91214445	intergenic	none	9.59	chr9:45243942-45250716	CNVer
chr2:91339937	intergenic	none	2.07	chr7:61464579-61475813	CNVer
chr2:91359823	near-gene-5	none	3.02	chr16:33758958-33762607	CNVer
chr2:91408043	intergenic	none	6.6	chr9:46608158-46622405	Conrad
chr2:91437467	intron	LOC389000	16.36	chr10:42088872-42089949	CNVer
chr2:91448574	intron	LOC389000	6.49	chr10:38987061-38988298	CNVer
chr2:91451837	intron	LOC389000	11.54	chr10:38990419-38991608	CNVer
chr2:91454972	intron	LOC389000	8.93	chr10:38993653-38994719	CNVer
chr2:91463970	intron	LOC389000	6.76	chr10:39002877-39003896	CNVer
chr2:91465823	CDS	LOC389000	8.19	chr10:42066095-42067205	CNVer
chr2:91467172	intron	LOC389000	21.46	chr10:39005828-39006982	CNVer
chr2:91525618	intergenic	none	7.83	chr16:33265033-33266295	CNVer
chr2:91528062	intergenic	none	2.46	chr22:15378002-15385727	CNVer
chr2:91544986	intergenic	none	2.19	chr15:18598093-18607933	CNVer
chr2:91555069	intergenic	none	9.18	chr9:67053302-67055968	CNVer
chr2:91595954	intergenic	none	3.02	chr16:33758958-33762607	CNVer
chr2:91617963	intergenic	none	13.05	chr7:53190063-53193070	CNVer
chr2:91617963	intergenic	none	4.56	chr1:147097193-147101336	CNVer
chr2:91620889	intergenic	none	18.52	chr16:32950741-32951758	CNVer
chr2:91627332	intergenic	none	4.79	chr7:61399743-61404853	CNVer
chr2:91627529	intergenic	none	4.61	chr16:34032757-34035162	CNVer

chr2:94745290	intergenic	none	4.6	chr9:42502910-42504305	CNVer
chr2:95867830	intergenic	none	5.67	chr2:97474200-97485211	CNVer
chr2:95877629	intergenic	none	16.5	chr2:88883245-88888495	CNVer
chr2:95887070	intron	LOC400986	4.27	chr2:97272741-97274055	CNVer
chr2:95963046	intron	LOC400986	37.5	chr2:88857969-88859821	CNVer
chr2:95965655	intron	LOC400986	95.25	chr2:88855580-88856634	CNVer
chr2:95967599	intron	LOC400986	117.56	chr2:88853868-88854904	CNVer
chr2:95978734	intron	LOC400986	16.99	chr2:97176540-97178359	CNVer
chr2:97176956	intergenic	none	14.69	chr2:97176540-97178359	CNVer
chr2:97183172	intergenic	none	48.43	chr2:97176540-97178359	CNVer
chr2:97196116	intergenic	none	57.26	chr2:88855580-88856634	CNVer
chr2:97543246	intron	ANKRD36B	5.39	chr2:97207601-97209992	CNVer
chr2:110343229	intergenic	none	2.34	chr2:110342773-110348620	Conrad
chr2:113874637	intergenic	none	5.17	chr2:113870126-113876423	CNVer
chr2:113984664	intergenic	none	10.79	chr9:70119907-70121600	CNVer
chr2:113984957	intergenic	none	25.29	chr9:68476584-68479189	CNVer
chr2:130409314	near-gene-5	none	4.0	chr14:19171146-19179499	CNVer
chr2:130498713	intergenic	none	12.55	chr14:18459973-18469836	CNVer
chr2:131652677	intergenic	none	3.96	chr14:19165798-19167632	CNVer
chr2:131785747	near-gene-3	none	5.25	chr14:18459973-18469836	CNVer
chr2:131788143	intergenic	none	3.0	chr14:18459973-18469836	CNVer
chr2:132236717	intron	C2orf27	9.67	chr9:69896618-69897954	CNVer
chr2:132236717	intron	C2orf27	8.95	chr14:18891569-18893565	CNVer
chr2:132241881	near-gene-3	none	2.86	chr9:69903225-69904875	CNVer
chr2:132246522	intron	LOC100128267	2.5	chr9:69907762-69908820	CNVer
chr2:132257939	intergenic	none	11.67	chr9:42273695-42275717	CNVer
chr2:132267859	near-gene-3	none	2.86	chr9:69903225-69904875	CNVer
chr2:132271822	intron	MGC50273	8.1	chr9:42252636-42255626	CNVer
chr2:132273188	intron	MGC50273	13.78	chr9:45318666-45323798	CNVer
chr2:132273209	intron	MGC50273	9.44	chr9:69896618-69897954	CNVer
chr2:132273209	intron	MGC50273	7.81	chr14:18891569-18893565	CNVer
chr2:132294697	near-gene-3	none	4.48	chr9:45302391-45304625	CNVer
chr2:132295104	near-gene-3	none	12.03	chr9:69910950-69917457	CNVer
chr2:132490257	intergenic	none	11.0	chr9:69462514-69465468	CNVer
chr2:132499566	intergenic	none	15.05	chr7:64597627-64598678	CNVer
chr2:132518254	intergenic	none	23.43	chr7:64617298-64618615	CNVer
chr2:132521673	intergenic	none	7.31	chr7:61399743-61404853	CNVer
chr2:132524267	intergenic	none	4.72	chr7:61399743-61404853	CNVer
chr2:132524298	intergenic	none	7.67	chr16:32022274-32023327	CNVer
chr2:132524298	intergenic	none	5.0	chr16:32964971-32967881	CNVer
chr2:132527823	intergenic	none	7.41	chr1:147089712-147090923	CNVer
chr2:132528863	intergenic	none	16.25	chr16:33742239-33743375	CNVer
chr2:132700282	intergenic	none	38.37	chr21:13288477-13291647	CNVer
chr2:132756945	intergenic	none	4.1	chr4:49002486-49004520	CNVer
chr2:132832639	intergenic	none	14.1	chr9:45240562-45242725	CNVer
chr2:132833870	intergenic	none	8.0	chr9:45240562-45242725	CNVer
chr3:36816044	intergenic	none	2.0	chr10:42074411-42076988	CNVer
chr3:75584009	intergenic	none	3.08	chr8:12355582-12357292	CNVer
chr3:75589830	intergenic	none	4.0	chr8:12370301-12372031	CNVer
chr3:75595645	intergenic	none	2.5	chr8:12372363-12378948	CNVer
chr3:75633064	intergenic	none	7.82	chr8:8038885-8042037	CNVer
chr3:75635598	intergenic	none	8.27	chr4:4086972-4088634	CNVer
chr3:75682844	intergenic	none	3.0	chr11:3426105-3434703	CNVer
chr3:75685233	intergenic	none	8.31	chr8:8093234-8101863	CNVer
chr3:75685233	intergenic	none	6.97	chr4:9270327-9272896	CNVer
chr3:75685233	intergenic	none	4.67	chr11:3426105-3434703	CNVer
chr3:75689183	intergenic	none	13.7	chr8:8093234-8101863	CNVer
chr3:75689307	intergenic	none	7.67	chr4:9275791-9277046	CNVer
chr3:104821389	intergenic	none	2.13	chr22:15401415-15402820	CNVer
chr3:104821389	intergenic	none	2.13	chr15:18594643-18595685	CNVer
chr3:125201665	intergenic	none	5.5	chr3:127155055-127161736	Conrad
chr3:126927508	intergenic	none	4.0	chr3:126927150-126928480	CNVer
chr3:126949876	intergenic	none	5.2	chr3:126927150-126928480	CNVer
chr3:127044507	intergenic	none	7.9	chr8:12416498-12420432	CNVer
chr3:131321781	intergenic	none	2.55	chr8:8104169-8105213	CNVer
chr3:131326982	intergenic	none	11.4	chr8:12370301-12372031	CNVer
chr3:188067403	intergenic	none	5.4	chr20:3960496-3964421	Conrad
chr3:196825780	intergenic	none	4.57	chr3:196824149-196827185	CNVer
chr3:196879334	intergenic	none	13.27	chr3:196878463-196879952	CNVer
chr3:196897770	intergenic	none	25.17	chr3:197174126-197175161	CNVer
chr3:196937624	CDS	MUC20	9.0	chr3:196827581-196830267	CNVer
chr3:196940631	intron	MUC20	2.0	chr6:57730820-57739182	Conrad
chr3:196961769	intron	MUC4	8.0	chr3:196827581-196830267	CNVer
chr3:197148075	CDS	LOC727978	26.93	chr3:198844375-198845433	CNVer
chr3:197149586	intron	LOC727978	37.05	chr3:198845894-198847376	CNVer
chr3:197150946	intron	LOC727978	24.0	chr3:198848089-198852676	CNVer
chr3:197154717	near-gene-3	none	9.53	chr3:197213947-197216604	CNVer
chr3:197159273	intergenic	none	9.54	chr3:197158926-197160025	CNVer
chr3:197169727	intergenic	none	3.1	chr3:197169245-197170447	CNVer
chr3:197174281	intergenic	none	18.6	chr3:197174126-197175161	CNVer
chr3:197187905	intergenic	none	15.84	chr3:196881250-196884352	CNVer
chr3:197198264	intergenic	none	7.07	chr3:197194250-197198978	CNVer
chr3:197209100	intergenic	none	11.98	chr3:198845894-198847376	CNVer
chr3:198829259	intergenic	none	2.95	chr3:196878463-196879952	CNVer
chr3:198840371	near-gene-5	none	8.34	chr3:196867225-196868813	CNVer
chr3:198845146	CDS	LOC728210	2.3	chr3:198844375-198845433	CNVer
chr4:10323	intergenic	none	12.42	chr4:191260143-191262009	CNVer
chr4:4023461	intergenic	none	31.99	chr8:12354011-12355074	CNVer
chr4:4026728	intergenic	none	11.28	chr8:8104169-8105213	CNVer

chr4:4027608	intergenic	none	2.4	chr8:12358848-12360629	CNVer
chr4:4031476	intergenic	none	11.58	chr4:9275791-9277046	CNVer
chr4:4178189	intergenic	none	13.32	chr3:126927150-126928480	CNVer
chr4:9212716	intergenic	none	4.25	chr4:4086972-4088634	CNVer
chr4:9245484	intergenic	none	5.3	chr8:8058579-8064372	CNVer
chr4:9278597	intergenic	none	3.09	chr8:12358848-12360629	CNVer
chr4:9281104	intergenic	none	12.63	chr8:8104169-8105213	CNVer
chr4:9284366	intergenic	none	6.9	chr8:12354011-12355074	CNVer
chr4:48807959	intergenic	none	3.25	chr4:49327780-49343325	CNVer
chr4:48852361	intergenic	none	5.24	chr4:49327780-49343325	CNVer
chr4:48893822	intergenic	none	2.69	chr4:48884270-48896909	CNVer
chr4:48917092	intergenic	none	48.1	chr1:142012764-142014141	CNVer
chr4:48926877	intergenic	none	21.25	chr1:142021635-142023680	CNVer
chr4:48942416	intergenic	none	27.25	chr1:142184499-142190108	CNVer
chr4:48981115	intergenic	none	6.11	chr4:49213211-49214569	CNVer
chr4:48981336	intergenic	none	13.58	chr4:49026784-49031019	CNVer
chr4:48993255	intergenic	none	6.63	chr4:49213211-49214569	CNVer
chr4:49001236	intergenic	none	8.78	chr4:49017970-49020759	CNVer
chr4:49007372	intergenic	none	14.2	chr4:48999825-49002297	CNVer
chr4:49007413	intergenic	none	4.2	chr4:49017970-49020759	CNVer
chr4:49015021	intergenic	none	5.6	chr4:49213211-49214569	CNVer
chr4:49022163	intergenic	none	2.84	chr4:49213211-49214569	CNVer
chr4:49206895	intergenic	none	43.57	chr4:49213211-49214569	CNVer
chr4:69147565	intergenic	none	2.99	chr4:4033386-4035060	CNVer
chr4:72397054	intron	SLC4A4	2.0	chr10:69010337-69012300	CNVer
chr4:104056017	intron	NHEDC1	6.03	chr10:42081559-42083426	CNVer
chr4:104058339	intron	NHEDC1	19.98	chr10:38990419-38991608	CNVer
chr4:104073784	intron	NHEDC1	4.17	chr10:39005828-39006982	CNVer
chr4:120549830	utr-3	FLJ14186	4.81	chr4:119771214-119772908	CNVer
chr4:120552099	intron	FLJ14186	4.47	chr1:659461-660526	CNVer
chr4:132840273	intergenic	none	2.5	chr9:67428247-67432652	CNVer
chr4:132846144	intergenic	none	5.0	chr9:45566439-45567518	CNVer
chr4:132886888	utr-5	LOC100131591	2.19	chr4:132879225-132880382	CNVer
chr4:132976819	intergenic	none	7.91	chr9:45473950-45474978	CNVer
chr4:133009896	intergenic	none	2.62	chr9:67678217-67680066	CNVer
chr4:133034730	intergenic	none	13.28	chr9:42157383-42158383	CNVer
chr4:133068832	intergenic	none	8.8	chr9:67740108-67741512	CNVer
chr4:133072825	intergenic	none	6.93	chr9:42196632-42198736	CNVer
chr4:133096707	intergenic	none	4.89	chr9:69865949-69867013	CNVer
chr4:165400551	intergenic	none	3.34	chr1:239798-243630	CNVer
chr4:165400551	intergenic	none	3.34	chr1:239301-243900	Yoon
chr4:171816479	intergenic	none	3.55	chr1:146586407-146588170	CNVer
chr4:191038051	intergenic	none	55.81	chr9:68060623-68063171	CNVer
chr4:191038446	intergenic	none	3.39	chr9:67998454-68003901	CNVer
chr4:191043480	intergenic	none	21.64	chr9:67993756-67995045	CNVer
chr4:191043480	intergenic	none	11.9	chr9:68067287-68068390	CNVer
chr4:191050347	intergenic	none	5.0	chr9:67986379-67987980	CNVer
chr4:191100462	intron	FRG1	21.48	chr9:68353754-68357626	CNVer
chr4:191105726	intron	FRG1	33.2	chr9:68361452-68362836	CNVer
chr5:274293	intron	SDHA	9.81	chr3:196869411-196872381	CNVer
chr5:282308	intron	SDHA	12.84	chr3:196878463-196879952	CNVer
chr5:284691	intron	SDHA	11.0	chr3:196881250-196884352	CNVer
chr5:293949	intron	SDHA	3.5	chr3:197177607-197183080	CNVer
chr5:795087	intron	ZDHHC11B	9.0	chr5:886625-888016	CNVer
chr5:805098	intron	ZDHHC11B	3.5	chr5:896282-898396	CNVer
chr5:887376	intron	ZDHHC11	6.0	chr5:886625-888016	CNVer
chr5:897710	intron	ZDHHC11	2.5	chr5:896282-898396	CNVer
chr5:899867	intron	ZDHHC11	3.05	chr5:898505-900195	CNVer
chr5:1380208	intron	CLPTM1L	4.91	chr5:1380243-1381946	CNVer
chr5:21529365	near-gene-5	none	2.02	chr5:21528645-21530277	CNVer
chr5:34407264	intergenic	none	35.0	chr5:20868352-20870585	CNVer
chr5:37521982	intron	WDR70	6.74	chr5:21524976-21527051	CNVer
chr5:37521982	intron	WDR70	4.75	chr5:69540732-69542332	CNVer
chr5:49443745	intergenic	none	25.53	chr19:24407258-24423489	CNVer
chr5:49446002	intergenic	none	37.83	chr19:24407258-24423489	CNVer
chr5:49448169	intergenic	none	24.79	chr19:24407258-24423489	CNVer
chr5:49450383	intergenic	none	23.22	chr19:24407258-24423489	CNVer
chr5:49452597	intergenic	none	22.66	chr19:24407258-24423489	CNVer
chr5:49454780	intergenic	none	11.07	chr19:24407258-24423489	CNVer
chr5:49457004	intergenic	none	13.85	chr19:24407258-24423489	CNVer
chr5:49459239	intergenic	none	21.23	chr19:24407258-24423489	CNVer
chr5:49461453	intergenic	none	20.56	chr19:24407258-24423489	CNVer
chr5:79983494	intron	DHFR	3.0	chr5:79981609-79984011	Conrad
chr5:98898838	intron	LOC728104	29.67	chr5:34214322-34215802	CNVer
chr5:98899709	intron	LOC728104	3.25	chr5:70551964-70553208	CNVer
chr5:99739589	intergenic	none	5.24	chr5:34214322-34215802	CNVer
chr5:99740477	intergenic	none	4.0	chr5:21528645-21530277	CNVer
chr5:153999679	intergenic	none	26.04	chr4:4033386-4035060	CNVer
chr5:180763426	intergenic	none	5.44	chr1:222174594-222177176	CNVer
chr5-h2-hap1:1281273	intergenic	none	2.33	chr5:21528645-21530277	CNVer
chr6:24791875	intron	THEM2	31.0	chr22:31257932-31258517	Conrad
chr6:26828379	intergenic	none	2.84	chr6:26843718-26852707	CNVer
chr6:26835650	intergenic	none	2.34	chr6:26859118-26861839	CNVer
chr6:26853359	intergenic	none	4.34	chr6:26827710-26830024	CNVer
chr6:26881854	intergenic	none	2.34	chr6:26859118-26861839	CNVer
chr6:26889333	intergenic	none	3.84	chr6:26843718-26852707	CNVer
chr6:26960448	intergenic	none	2.73	chr5:69245742-69247726	CNVer
chr6:26977236	intergenic	none	4.75	chr5:69228376-69231317	CNVer
chr6:42973733	intergenic	none	3.66	chr4:4033386-4035060	CNVer

chr6:42974012	intergenic	none	12.89	chr8:8093234-8101863	CNVer
chr6:42974012	intergenic	none	12.89	chr11:3426105-3434703	CNVer
chr6:58256538	intergenic	none	8.52	chr6:26827710-26830024	CNVer
chr6:58362115	intergenic	none	2.22	chr5:21528645-21530277	CNVer
chr6:58373999	near-gene-3	none	3.42	chr5:68968291-68969713	CNVer
chr6:89412762	intron	RNGTT	3.0	chr16:33240125-33253092	CNVer
chr6:89412762	intron	RNGTT	3.0	chr22:15401415-15402820	CNVer
chr6:89412762	intron	RNGTT	3.0	chr15:18594643-18595685	CNVer
chr6:108138173	intergenic	none	3.0	chr6:108138045-108139210	Conrad
chr6:108143180	intergenic	none	6.0	chr6:108138045-108139210	Conrad
chr6:108143180	intergenic	none	6.0	chr6:108132828-108141795	Conrad
chr6:151410315	intron	MTHFD1L	7.47	chr9:66755331-66756878	CNVer
chr6:170829739	intergenic	none	2.8	chr1:393017-394466	CNVer
chr7:6974677	intergenic	none	6.25	chr8:12404377-12414508	CNVer
chr7:29681790	intergenic	none	2.0	chr7:32741081-32744772	Conrad
chr7:32743374	intergenic	none	2.9	chr7:32741081-32744772	Conrad
chr7:51429035	near-gene-5	none	2.0	chr1:659461-660526	CNVer
chr7:53162824	intergenic	none	4.18	chr9:42723944-42727628	CNVer
chr7:53168734	intergenic	none	8.55	chr16:32022274-32023327	CNVer
chr7:53170112	intergenic	none	6.16	chr7:61477981-61479977	CNVer
chr7:53172410	intergenic	none	3.58	chr9:69454665-69458531	CNVer
chr7:53172876	intergenic	none	8.0	chr7:64204600-64205715	CNVer
chr7:53172891	intergenic	none	3.95	chr16:33742239-33743375	CNVer
chr7:53173078	intergenic	none	2.87	chr13:17981001-17990854	CNVer
chr7:53187389	intergenic	none	5.41	chr16:32950741-32951758	CNVer
chr7:53191104	intergenic	none	20.54	chr9:69462514-69465468	CNVer
chr7:53194058	intergenic	none	3.15	chr7:61367488-61373808	CNVer
chr7:53197934	intergenic	none	14.65	chr7:64597627-64598678	CNVer
chr7:53200481	intergenic	none	13.11	chr7:64600070-64602181	CNVer
chr7:54261251	intergenic	none	2.0	chr7:54348136-54355770	Conrad
chr7:54350560	intergenic	none	2.0	chr7:54348136-54355770	Conrad
chr7:57684321	intergenic	none	3.59	chr17:22137357-22144896	CNVer
chr7:57707101	intergenic	none	2.67	chr20:25778776-25779855	CNVer
chr7:57726542	intergenic	none	24.91	chr7:57726342-57728045	CNVer
chr7:57781058	intergenic	none	17.89	chr20:25859292-25860770	CNVer
chr7:57797046	intergenic	none	10.94	chr20:25875365-25876407	CNVer
chr7:57802039	intergenic	none	18.61	chr20:25880315-25881485	CNVer
chr7:57804127	intergenic	none	21.14	chr20:28099038-28100281	CNVer
chr7:57875758	intergenic	none	7.57	chr7:57875926-57879690	CNVer
chr7:57908384	intergenic	none	3.59	chr17:22137357-22144896	CNVer
chr7:57921886	intergenic	none	21.96	chr16:32015853-32017059	CNVer
chr7:57925133	intergenic	none	8.14	chr7:61367488-61373808	CNVer
chr7:57926152	intergenic	none	2.5	chr1:146929658-146949571	CNVer
chr7:57933474	intergenic	none	15.7	chr16:33758958-33762607	CNVer
chr7:57938319	intergenic	none	24.81	chr7:64617298-64618615	CNVer
chr7:57942259	intergenic	none	3.45	chr7:61399743-61404853	CNVer
chr7:61063110	intergenic	none	7.36	chr7:61399743-61404853	CNVer
chr7:61069767	intergenic	none	7.68	chr7:64187507-64205734	Conrad
chr7:61075829	intergenic	none	3.53	chr16:33735234-33737733	CNVer
chr7:61077814	intergenic	none	7.68	chr16:32012045-32013573	CNVer
chr7:61373653	intergenic	none	61.08	chr7:64597627-64598678	CNVer
chr7:61375947	intergenic	none	4.03	chr7:57928826-57935924	CNVer
chr7:61398956	intergenic	none	11.62	chr9:42723944-42727628	CNVer
chr7:61402535	intergenic	none	2.55	chr7:61399743-61404853	CNVer
chr7:61407240	intergenic	none	29.9	chr1:147089712-147090923	CNVer
chr7:61407812	intergenic	none	3.98	chr7:64204600-64205715	CNVer
chr7:61408097	intergenic	none	10.2	chr16:33742239-33743375	CNVer
chr7:61442741	intergenic	none	59.08	chr7:64597627-64598678	CNVer
chr7:61461115	intergenic	none	8.38	chr16:33763306-33766812	CNVer
chr7:61470004	intergenic	none	3.65	chr13:17981001-17990854	CNVer
chr7:61476475	intergenic	none	4.95	chr7:64187507-64205734	Conrad
chr7:61481458	intergenic	none	5.71	chr16:32022274-32023327	CNVer
chr7:61482636	intergenic	none	8.0	chr16:34032757-34035162	CNVer
chr7:61530857	intergenic	none	102.8	chr7:61528923-61530951	CNVer
chr7:64192968	intergenic	none	3.9	chr7:64187507-64205734	Conrad
chr7:64204682	intron	LOC100128294	2.96	chr13:17981001-17990854	CNVer
chr7:64205616	intron	LOC100128294	16.8	chr1:147089712-147090923	CNVer
chr7:64208709	intergenic	none	3.96	chr7:61399743-61404853	CNVer
chr7:64209406	intergenic	none	3.89	chr7:61063527-61065585	CNVer
chr7:64211436	intergenic	none	4.6	chr7:61399743-61404853	CNVer
chr7:64215305	intergenic	none	25.02	chr7:64617298-64618615	CNVer
chr7:64219387	intergenic	none	10.89	chr16:33758958-33762607	CNVer
chr7:64239900	intergenic	none	4.05	chr16:32943979-32947198	CNVer
chr7:64240821	intergenic	none	3.49	chr16:33763306-33766812	CNVer
chr7:64363187	intergenic	none	5.07	chr20:3960496-3964421	Conrad
chr7:64602510	intergenic	none	2.1	chr16:32040890-32058514	CNVer
chr7:64605335	intergenic	none	4.08	chr16:33763306-33766812	CNVer
chr7:64608636	intergenic	none	4.49	chr16:33763306-33766812	CNVer
chr7:64609573	intergenic	none	4.38	chr16:32943979-32947198	CNVer
chr7:64613353	intergenic	none	6.32	chr16:33758958-33762607	CNVer
chr7:64619504	intergenic	none	4.29	chr7:64675380-64682932	CNVer
chr7:64652112	intergenic	none	11.32	chr7:64649866-64652666	CNVer
chr7:64655367	intergenic	none	3.1	chr16:32943979-32947198	CNVer
chr7:64675698	intergenic	none	10.89	chr16:33758958-33762607	CNVer
chr7:64680655	intergenic	none	24.81	chr7:64617298-64618615	CNVer
chr7:64684368	intergenic	none	2.97	chr7:61399743-61404853	CNVer
chr7:64684776	intergenic	none	2.65	chr7:64684691-64685851	CNVer
chr7:64686243	intergenic	none	7.42	chr7:61063527-61065585	CNVer
chr7:64690472	intergenic	none	14.01	chr1:147089712-147090923	CNVer

chr7:64751079	intergenic	none	2.22	chr16:32943979-32947198	CNVer
chr7:64754692	intergenic	none	5.87	chr7:64649866-64652666	CNVer
chr7:64888742	intergenic	none	6.0	chr7:64187507-64205734	Conrad
chr7:64900420	intergenic	none	4.0	chr1:120574865-120585820	CNVer
chr7:64900420	intergenic	none	4.0	chr1:142679389-142684202	CNVer
chr7:65984634	near-gene-3	none	3.0	chr7:64898349-64908733	CNVer
chr7:66335561	intron	TYW1	3.0	chr7:66335018-66336838	Conrad
chr7:100423034	utr-5	MUC12	4.85	chr7:100424431-100434217	CNVer
chr7:101941441	intergenic	none	2.0	chr7:102037393-102045017	CNVer
chr7:102040635	intron	RASA4	2.0	chr7:102037393-102045017	CNVer
chr7:102103101	intergenic	none	3.94	chr7:102102814-102104383	CNVer
chr7:102444499	intron	FBXL13	6.0	chr7:102135311-102152748	Conrad
chr8:23227	intergenic	none	42.02	chr5:180809672-180811063	CNVer
chr8:43442	intergenic	none	3.07	chr1:544284-545519	CNVer
chr8:54911	intergenic	none	2.04	chr1:407734-408951	CNVer
chr8:67569	intergenic	none	2.51	chr1:222173302-222174459	CNVer
chr8:152994	intergenic	none	16.9	chr1:247191801-247199100	Yoon
chr8:157406	intergenic	none	2.15	chr1:220901-230600	Yoon
chr8:6866969	intergenic	none	45.84	chr8:6847435-6854770	CNVer
chr8:7144247	near-gene-3	none	4.5	chr8:7653176-7654749	CNVer
chr8:7179869	near-gene-5	none	10.81	chr8:12284101-12286074	CNVer
chr8:7391269	intron	LOC100131608	8.0	chr8:7661310-7664776	CNVer
chr8:7391269	intron	LOC100131608	7.98	chr8:7630719-7632192	CNVer
chr8:7663383	near-gene-5	none	2.78	chr8:7655773-7657584	CNVer
chr8:7669411	utr-5	LOC100133099	2.61	chr8:7661310-7664776	CNVer
chr8:7669411	utr-5	LOC100133099	2.21	chr8:7630719-7632192	CNVer
chr8:7869789	near-gene-3	none	10.39	chr8:12284101-12286074	CNVer
chr8:7905101	intergenic	none	4.5	chr8:7653176-7654749	CNVer
chr8:8104131	intergenic	none	5.92	chr8:8104169-8105213	CNVer
chr8:8105868	intergenic	none	3.56	chr8:12355582-12357292	CNVer
chr8:8107809	intergenic	none	4.74	chr8:12354011-12355074	CNVer
chr8:8111370	intergenic	none	3.5	chr8:12348581-12352214	CNVer
chr8:12280984	intergenic	none	2.0	chr8:12275661-12276963	CNVer
chr8:12356427	intergenic	none	4.5	chr8:12355582-12357292	CNVer
chr8:12357762	intergenic	none	4.64	chr8:8104169-8105213	CNVer
chr8:12426910	intergenic	none	3.21	chr4:4086972-4088634	CNVer
chr8:53104990	intergenic	none	2.0	chr1:16739901-16741500	Yoon
chr8:63177888	intergenic	none	2.0	chr1:553585-560421	CNVer
chr8:63177888	intergenic	none	2.0	chr1:554301-560300	Yoon
chr8:63177888	intergenic	none	2.0	chr1:554403-560267	Conrad
chr8:86748949	intergenic	none	77.92	chr8:86893085-86894819	CNVer
chr8:86748949	intergenic	none	55.29	chr8:86905356-86908485	CNVer
chr8:86854771	intergenic	none	66.32	chr8:86893085-86894819	CNVer
chr8:86854771	intergenic	none	48.66	chr8:86905356-86908485	CNVer
chr8:86866962	intergenic	none	78.49	chr8:86893085-86894819	CNVer
chr8:86866962	intergenic	none	57.63	chr8:86905356-86908485	CNVer
chr8:86879153	intergenic	none	70.52	chr8:86893085-86894819	CNVer
chr8:86879153	intergenic	none	52.35	chr8:86905356-86908485	CNVer
chr8:86891067	intergenic	none	67.46	chr8:86893085-86894819	CNVer
chr8:86891067	intergenic	none	49.32	chr8:86905356-86908485	CNVer
chr8:86903257	intergenic	none	85.32	chr8:86893085-86894819	CNVer
chr8:86903257	intergenic	none	60.77	chr8:86905356-86908485	CNVer
chr8:129696095	intergenic	none	5.0	chr19:48519805-48525213	CNVer
chr9:163699	intron	CBWD1	2.5	chr9:70048367-70051832	CNVer
chr9:185935	intergenic	none	8.34	chr9:70027998-70029749	CNVer
chr9:33512133	near-gene-5	none	16.11	chr9:99025912-99028202	CNVer
chr9:39128067	intron	CNTNAP3	4.03	chr9:65336890-65338996	CNVer
chr9:39130481	intron	CNTNAP3	5.12	chr9:65334387-65335537	CNVer
chr9:39130481	intron	CNTNAP3	4.12	chr9:43792478-43795183	CNVer
chr9:41884176	intergenic	none	5.91	chr9:44403744-44407188	CNVer
chr9:41908593	intergenic	none	7.75	chr9:41908035-41909229	CNVer
chr9:41930102	intergenic	none	5.5	chr9:66307275-66309433	CNVer
chr9:41952336	intergenic	none	8.23	chr9:44243216-44244387	CNVer
chr9:42012846	utr-3	LOC100132442	2.34	chr9:41940093-41941174	CNVer
chr9:42045638	intergenic	none	2.62	chr9:45527764-45529243	CNVer
chr9:42159041	intergenic	none	9.32	chr9:67644677-67648851	CNVer
chr9:42181410	intergenic	none	5.54	chr9:45390928-45392538	CNVer
chr9:42219310	intergenic	none	4.32	chr9:67765962-67767065	CNVer
chr9:42221600	intergenic	none	3.97	chr9:69865949-69867013	CNVer
chr9:42238049	intergenic	none	2.09	chr9:45334426-45335974	CNVer
chr9:42249798	intergenic	none	2.09	chr9:67795537-67804278	CNVer
chr9:42276001	intron	LOC100132149	2.34	chr9:45295377-45296746	CNVer
chr9:42503807	intergenic	none	4.84	chr9:42502910-42504305	CNVer
chr9:42596991	intergenic	none	2.62	chr9:66941450-66942700	CNVer
chr9:42769274	intergenic	none	11.68	chr10:39095189-39096688	CNVer
chr9:42775056	intergenic	none	9.39	chr2:89939919-89941263	CNVer
chr9:42780078	intergenic	none	2.18	chr2:87444411-87463905	CNVer
chr9:42794437	intergenic	none	2.85	chr10:42052946-42056024	CNVer
chr9:42819888	intergenic	none	13.06	chr9:69240909-69244126	CNVer
chr9:42859777	intergenic	none	2.17	chr9:42859646-42860660	CNVer
chr9:42977910	intergenic	none	4.84	chr9:42502910-42504305	CNVer
chr9:44129320	intergenic	none	3.62	chr9:66271475-66274220	CNVer
chr9:44133178	intergenic	none	2.82	chr9:66275301-66277138	CNVer
chr9:44139834	intergenic	none	2.64	chr9:41895796-41899515	CNVer
chr9:44149344	intergenic	none	2.34	chr9:41908035-41909229	CNVer
chr9:44356202	intergenic	none	2.0	chr9:66307275-66309433	CNVer
chr9:44377586	intergenic	none	6.45	chr9:41908035-41909229	CNVer
chr9:44382654	intergenic	none	2.73	chr9:41903048-41904614	CNVer
chr9:44396677	intergenic	none	3.03	chr9:66271475-66274220	CNVer

chr9:44401987	intergenic	none	5.19	chr9:66264696-66270732	CNVer
chr9:44804248	intergenic	none	2.4	chr9:46016555-46018742	CNVer
chr9:44841625	intergenic	none	6.5	chr20:25893278-25894304	CNVer
chr9:45248705	near-gene-5	none	5.07	chr9:45243942-45250716	CNVer
chr9:45264518	intergenic	none	3.84	chr9:45261581-45262960	CNVer
chr9:45268841	intergenic	none	2.75	chr9:67843971-67845182	CNVer
chr9:45335233	intergenic	none	3.6	chr9:45334426-45335974	CNVer
chr9:45349478	intergenic	none	2.25	chr9:67770894-67772233	CNVer
chr9:45353824	intergenic	none	5.82	chr9:67765962-67767065	CNVer
chr9:45353824	intergenic	none	4.72	chr9:45353546-45354560	CNVer
chr9:45368404	intergenic	none	6.67	chr9:42203948-42205363	CNVer
chr9:45391254	intergenic	none	12.59	chr9:45390928-45392538	CNVer
chr9:45413554	intergenic	none	6.03	chr9:67644677-67648851	CNVer
chr9:45431757	intergenic	none	2.26	chr9:45430691-45432043	CNVer
chr9:45619186	near-gene-5	none	3.1	chr9:44932412-44933857	CNVer
chr9:45619186	near-gene-5	none	3.1	chr9:67380351-67381773	CNVer
chr9:45799786	intergenic	none	8.19	chr9:67644677-67648851	CNVer
chr9:45818004	intergenic	none	3.01	chr9:45430691-45432043	CNVer
chr9:46490961	intergenic	none	2.62	chr9:45527764-45529243	CNVer
chr9:46698128	intergenic	none	2.0	chr9:66307275-66309433	CNVer
chr9:46719808	intergenic	none	9.09	chr9:41908035-41909229	CNVer
chr9:66018170	intergenic	none	42.94	chr9:42548199-42549243	CNVer
chr9:66067582	intergenic	none	2.8	chr9:42498639-42501044	CNVer
chr9:66070721	intergenic	none	4.0	chr9:68802584-68805807	CNVer
chr9:66235367	near-gene-5	none	7.45	chr9:45243942-45250716	CNVer
chr9:66246468	intergenic	none	4.17	chr9:67854013-67855207	CNVer
chr9:66251449	intergenic	none	4.84	chr9:45261581-45262960	CNVer
chr9:66256248	intergenic	none	3.0	chr9:67843971-67845182	CNVer
chr9:66262839	near-gene-3	none	2.49	chr9:41881956-41883118	CNVer
chr9:66277938	intron	FLJ20444	20.69	chr9:41895796-41899515	CNVer
chr9:66285280	intron	FLJ20444	8.75	chr9:44243216-44244387	CNVer
chr9:66307121	intergenic	none	5.5	chr9:66307275-66309433	CNVer
chr9:66329469	intergenic	none	6.12	chr9:41908035-41909229	CNVer
chr9:66333959	intergenic	none	2.3	chr9:41903048-41904614	CNVer
chr9:66347980	intergenic	none	2.52	chr9:66271475-66274220	CNVer
chr9:66512545	intergenic	none	5.55	chr21:13288477-13291647	CNVer
chr9:66516755	intergenic	none	11.32	chr9:66721825-66725541	CNVer
chr9:66556668	intergenic	none	3.0	chr9:67005807-67010766	CNVer
chr9:66670354	intergenic	none	11.51	chr10:39095189-39096688	CNVer
chr9:66676095	intergenic	none	9.39	chr2:89939919-89941263	CNVer
chr9:66696257	intergenic	none	2.85	chr10:42052946-42056024	CNVer
chr9:66729560	intron	LOC653458	38.62	chr9:66728130-66730631	CNVer
chr9:66742538	intron	LOC653458	11.12	chr9:69240909-69244126	CNVer
chr9:66782603	intron	LOC653458	2.17	chr9:42859646-42860660	CNVer
chr9:67014342	intergenic	none	7.9	chr9:66706101-66707118	CNVer
chr9:67021425	intron	LOC728611	10.21	chr15:19633034-19634204	CNVer
chr9:67031124	near-gene-5	none	2.27	chr9:67019816-67031845	CNVer
chr9:67052627	intergenic	none	14.01	chr15:18594643-18595685	CNVer
chr9:67428786	intergenic	none	3.24	chr9:67428247-67432652	CNVer
chr9:67472533	intergenic	none	2.62	chr9:45527764-45529243	CNVer
chr9:67659813	intergenic	none	3.04	chr9:45459550-45461216	CNVer
chr9:67672455	near-gene-5	none	17.28	chr9:46411296-46412376	CNVer
chr9:67678435	intergenic	none	6.11	chr9:42130132-42131155	CNVer
chr9:67678435	intergenic	none	4.57	chr9:67678217-67680066	CNVer
chr9:67680088	intergenic	none	5.69	chr9:45439805-45440854	CNVer
chr9:67697228	intergenic	none	3.5	chr9:45421884-45423020	CNVer
chr9:67700833	intergenic	none	12.0	chr9:45804675-45805825	CNVer
chr9:67700833	intergenic	none	11.34	chr9:45418447-45420187	CNVer
chr9:67705032	intergenic	none	6.22	chr9:42157383-42158383	CNVer
chr9:67728927	intergenic	none	3.28	chr9:45390928-45392538	CNVer
chr9:67746386	intergenic	none	2.7	chr9:42199455-42200507	CNVer
chr9:67753956	intergenic	none	7.29	chr9:69851823-69853873	CNVer
chr9:67758623	intergenic	none	2.5	chr9:42211303-42212696	CNVer
chr9:67765951	intergenic	none	2.36	chr9:67765962-67767065	CNVer
chr9:67769273	intergenic	none	6.79	chr9:69865949-69867013	CNVer
chr9:67769273	intergenic	none	3.76	chr9:42221020-42223175	CNVer
chr9:67771770	intergenic	none	3.1	chr9:67770894-67772233	CNVer
chr9:67776798	intergenic	none	18.25	chr9:42228804-42230405	CNVer
chr9:67776798	intergenic	none	17.25	chr9:69873449-69874522	CNVer
chr9:67797427	intergenic	none	2.0	chr14:18894028-18899024	CNVer
chr9:67806823	intergenic	none	3.46	chr9:69903225-69904875	CNVer
chr9:67831742	intergenic	none	12.98	chr9:45280613-45293533	CNVer
chr9:67834567	intergenic	none	15.27	chr9:67834256-67835872	CNVer
chr9:67836333	intergenic	none	46.77	chr9:42292755-42294280	CNVer
chr9:67841163	intergenic	none	8.87	chr9:67840941-67842041	CNVer
chr9:67843738	intergenic	none	3.5	chr9:67843971-67845182	CNVer
chr9:67848952	intergenic	none	9.93	chr9:45261581-45262960	CNVer
chr9:67938584	intergenic	none	103.87	chr9:68361452-68362836	CNVer
chr9:67987003	intergenic	none	9.64	chr9:67986379-67987980	CNVer
chr9:67987038	intergenic	none	12.91	chr9:68069726-68075598	CNVer
chr9:67993826	intergenic	none	18.43	chr9:68067287-68068390	CNVer
chr9:67995794	intergenic	none	16.41	chr9:68064263-68065812	CNVer
chr9:67995917	intergenic	none	8.5	chr9:67995528-67997322	CNVer
chr9:68001700	intergenic	none	16.05	chr9:68054118-68056066	CNVer
chr9:68067319	intergenic	none	10.99	chr9:67993756-67995045	CNVer
chr9:68292574	intergenic	none	65.6	chr9:67998454-68003901	CNVer
chr9:68292953	intergenic	none	19.9	chr9:68054118-68056066	CNVer
chr9:68297469	intergenic	none	4.17	chr9:68296078-68298426	CNVer
chr9:68301623	intergenic	none	4.0	chr9:68060623-68063171	CNVer

chr9:68306343	intergenic	none	10.99	chr9:67993756-67995045	CNVer
chr9:68625230	intergenic	none	5.2	chr9:67472477-67475171	CNVer
chr9:68799872	intergenic	none	10.94	chr9:66073138-66074476	CNVer
chr9:68799872	intergenic	none	8.61	chr9:42492389-42494231	CNVer
chr9:68810649	intergenic	none	9.91	chr9:42502910-42504305	CNVer
chr9:68827526	intergenic	none	2.34	chr9:42519158-42522269	CNVer
chr9:68926788	intergenic	none	2.34	chr9:66963908-66965997	CNVer
chr9:68928578	intergenic	none	2.17	chr9:42859646-42860660	CNVer
chr9:68995285	intergenic	none	3.18	chr9:67005807-67010766	CNVer
chr9:69014892	intergenic	none	2.97	chr10:42052946-42056024	CNVer
chr9:69028389	intergenic	none	2.18	chr2:87444411-87463905	CNVer
chr9:69088554	intergenic	none	16.39	chr7:61063527-61065585	CNVer
chr9:69181331	intergenic	none	5.84	chr9:66448502-66451358	CNVer
chr9:69247022	intergenic	none	82.0	chr9:66721825-66725541	CNVer
chr9:69247022	intergenic	none	57.31	chr21:13276329-13280385	CNVer
chr9:69254032	intergenic	none	11.96	chr21:13284024-13287516	CNVer
chr9:69255181	intergenic	none	10.98	chr21:13288477-13291647	CNVer
chr9:69259447	intergenic	none	7.77	chr9:66721825-66725541	CNVer
chr9:69296407	intergenic	none	10.0	chr9:67005807-67010766	CNVer
chr9:69355570	intergenic	none	13.06	chr9:69240909-69244126	CNVer
chr9:69370204	intergenic	none	4.67	chr9:66706101-66707118	CNVer
chr9:69380397	near-gene-5	none	2.97	chr10:42052946-42056024	CNVer
chr9:69395067	intergenic	none	2.07	chr2:87444411-87463905	CNVer
chr9:69472033	intron	LOC653510	2.14	chr9:68494301-68495887	CNVer
chr9:69581133	intergenic	none	2.97	chr10:42052946-42056024	CNVer
chr9:69595496	intergenic	none	2.18	chr2:87444411-87463905	CNVer
chr9:69804029	intergenic	none	18.54	chr9:67644677-67648851	CNVer
chr9:69825994	intergenic	none	4.99	chr9:45390928-45392538	CNVer
chr9:69828881	intergenic	none	19.59	chr9:67731592-67732808	CNVer
chr9:69866261	intergenic	none	4.47	chr9:69865949-69867013	CNVer
chr9:69882685	intergenic	none	2.81	chr9:45334426-45335974	CNVer
chr9:69921123	intergenic	none	4.17	chr9:45295377-45296746	CNVer
chr9:70028047	intergenic	none	8.34	chr9:70027998-70029749	CNVer
chr9:94694737	intergenic	none	5.13	chr1:142025320-142026647	CNVer
chr9:98969802	intergenic	none	2.12	chr1:142041405-142045305	CNVer
chr9:130654573	intron	CCBL1	6.06	chr4:4033386-4035060	CNVer
chr10:38932946	near-gene-5	none	8.93	chr16:33758958-33762607	CNVer
chr10:38948291	intergenic	none	3.08	chr9:45243942-45250716	CNVer
chr10:38967537	intergenic	none	2.84	chr10:42097806-42099094	CNVer
chr10:38976294	intergenic	none	5.98	chr10:42088872-42089949	CNVer
chr10:38991019	intergenic	none	8.73	chr10:38990419-38991608	CNVer
chr10:38996399	intergenic	none	27.14	chr10:42074411-42076988	CNVer
chr10:39003200	intergenic	none	10.61	chr10:39002877-39003896	CNVer
chr10:39004681	intergenic	none	6.0	chr10:42066095-42067205	CNVer
chr10:39055930	intergenic	none	12.81	chr16:33268646-33270453	CNVer
chr10:39076130	intergenic	none	4.4	chr2:87428172-87440514	CNVer
chr10:39076130	intergenic	none	3.91	chr9:67032919-67040524	CNVer
chr10:39080918	intergenic	none	5.07	chr2:89933842-89936761	CNVer
chr10:39088061	intergenic	none	7.92	chr2:110113086-110114649	CNVer
chr10:39089375	intergenic	none	29.49	chr10:42030558-42031624	CNVer
chr10:39095200	intergenic	none	13.17	chr10:39095189-39096688	CNVer
chr10:39095200	intergenic	none	7.17	chr10:42024985-42028375	CNVer
chr10:39096742	intergenic	none	12.1	chr10:42023152-42024631	CNVer
chr10:39106588	intergenic	none	31.56	chr10:42013148-42014513	CNVer
chr10:41697537	intergenic	none	5.66	chr10:41675055-41680434	CNVer
chr10:41925252	intergenic	none	10.5	chr16:34032757-34035162	CNVer
chr10:41931880	intergenic	none	28.18	chr16:32950741-32951758	CNVer
chr10:41934655	intergenic	none	26.13	chr7:53190063-53193070	CNVer
chr10:41943420	intergenic	none	4.24	chr13:17981001-17990854	CNVer
chr10:41947729	intergenic	none	4.33	chr7:61464579-61475813	CNVer
chr10:41972302	intergenic	none	8.28	chr9:69462514-69465468	CNVer
chr10:41980560	intergenic	none	6.5	chr7:57928826-57935924	CNVer
chr10:41987710	intergenic	none	8.79	chr7:57937378-57940816	CNVer
chr10:42014145	intergenic	none	16.4	chr10:42013148-42014513	CNVer
chr10:42031780	intergenic	none	4.3	chr15:18588821-18592313	CNVer
chr10:42037854	intergenic	none	6.95	chr9:66678904-66680067	CNVer
chr10:42042481	intergenic	none	6.5	chr9:67044163-67045630	CNVer
chr10:42042481	intergenic	none	5.74	chr2:87444411-87463905	CNVer
chr10:42065006	near-gene-5	none	7.4	chr10:39005828-39006982	CNVer
chr10:42080450	intron	LOC653097	19.14	chr10:38990419-38991608	CNVer
chr10:42100161	intergenic	none	9.43	chr10:42099536-42100976	CNVer
chr11:60614	intergenic	none	13.97	chr1:593611-595185	CNVer
chr11:60624	intergenic	none	11.73	chr1:593501-595200	Yoon
chr11:60626	intergenic	none	15.68	chr6:33721-35041	CNVer
chr11:169794	intergenic	none	31.0	chr3:199379408-199380561	CNVer
chr11:169808	intergenic	none	18.0	chr16:88710671-88711967	CNVer
chr11:3418491	intergenic	none	6.41	chr8:12354011-12355074	CNVer
chr11:3427122	intergenic	none	8.9	chr4:9275791-9277046	CNVer
chr11:3435783	intergenic	none	21.53	chr8:12370301-12372031	CNVer
chr11:3437581	intergenic	none	7.62	chr8:12372363-12378948	CNVer
chr11:3484305	intergenic	none	5.95	chr8:12416498-12420432	CNVer
chr11:3491707	intergenic	none	16.74	chr4:4086972-4088634	CNVer
chr11:3575967	intergenic	none	2.15	chr3:126927150-126928480	CNVer
chr11:4216068	intergenic	none	4.45	chr11:4261289-4263042	CNVer
chr11:4227023	intergenic	none	2.9	chr11:4300362-4301537	CNVer
chr11:4238725	intergenic	none	4.34	chr11:4333747-4335157	CNVer
chr11:4255844	intergenic	none	9.04	chr11:4209382-4210923	CNVer
chr11:4266041	near-gene-5	none	17.54	chr11:4209382-4210923	CNVer
chr11:4300936	intergenic	none	3.67	chr11:4300362-4301537	CNVer

chr11:4378527	intergenic	none	2.26	chr11:4326544-4327846	CNVer
chr11:5423396	intergenic	none	2.0	chr16:33240125-33253092	CNVer
chr11:5423396	intergenic	none	2.0	chr22:15401415-15402820	CNVer
chr11:5423396	intergenic	none	2.0	chr15:18594643-18595685	CNVer
chr11:60730305	intron	PGA3	2.0	chr11:60748348-60752986	CNVer
chr11:60747511	intron	PGA4	3.67	chr11:60746482-60747721	CNVer
chr11:60749134	intron	PGA4	2.0	chr11:60748348-60752986	CNVer
chr11:60766337	intron	PGA5	3.67	chr11:60746482-60747721	CNVer
chr11:60767960	intron	PGA5	2.0	chr11:60748348-60752986	CNVer
chr11:62048814	intron	AHNAK	2.0	chr11:62046469-62048814	CNVer
chr11:67321016	intergenic	none	2.17	chr11:71183131-71184533	CNVer
chr11:67354801	intergenic	none	10.34	chr11:3426105-3434703	CNVer
chr11:71149255	intergenic	none	3.45	chr8:12372363-12378948	CNVer
chr11:71152967	intergenic	none	9.34	chr4:9275791-9277046	CNVer
chr11:89184973	intergenic	none	2.34	chr11:89184665-89189576	CNVer
chr11:89291210	intron	LOC399939	3.95	chr11:54784823-54795083	Conrad
chr11:89298719	intron	LOC729384	28.56	chr11:54784823-54795083	Conrad
chr11:90348054	intergenic	none	2.2	chr8:81080152-81081761	CNVer
chr11:98506563	intron	CNTN5	3.0	chr9:46608158-46622405	Conrad
chr12:8301785	intergenic	none	5.0	chr8:12354011-12355074	CNVer
chr12:8304142	intergenic	none	2.0	chr8:12355582-12357292	CNVer
chr12:8308147	intergenic	none	4.45	chr8:8093234-8101863	CNVer
chr12:8348107	intergenic	none	3.74	chr8:12404377-12414508	CNVer
chr12:8351296	intergenic	none	16.8	chr8:12404377-12414508	CNVer
chr12:8355949	intergenic	none	2.0	chr8:12416498-12420432	CNVer
chr12:8366648	intergenic	none	7.51	chr4:4086972-4088634	CNVer
chr12:36191059	intergenic	none	18.65	chr20:28099038-28100281	CNVer
chr12:36201483	intergenic	none	4.35	chr20:25893278-25894304	CNVer
chr12:36202957	intergenic	none	4.17	chr20:28110161-28111358	CNVer
chr12:39165209	intergenic	none	20.96	chr12:39166103-39170290	CNVer
chr12:43366280	intron	NELL2	2.0	chr1:120843071-120846365	CNVer
chr12:52764847	intergenic	none	3.22	chr9:46016555-46018742	CNVer
chr12:56651064	intergenic	none	2.91	chr1:164447869-164461996	CNVer
chr12:69819920	intron	TSPAN8	89.0	chr12:69818942-69819932	Conrad
chr12:71525713	intergenic	none	48.0	chr15:92687327-92689599	Conrad
chr13:17987964	intergenic	none	4.19	chr7:61407779-61409446	CNVer
chr13:17989543	intergenic	none	3.85	chr7:64204600-64205715	CNVer
chr13:17992750	intergenic	none	8.0	chr18:15210407-15221255	CNVer
chr13:17992769	intergenic	none	14.0	chr13:17981001-17990854	CNVer
chr13:17994009	intergenic	none	3.87	chr1:147089712-147090923	CNVer
chr13:17997285	intergenic	none	7.9	chr7:61063527-61065585	CNVer
chr13:17997548	intergenic	none	15.25	chr16:32022274-32023327	CNVer
chr13:18320308	intergenic	none	4.13	chr1:142060549-142062057	CNVer
chr13:60303013	intergenic	none	7.41	chr7:102135311-102152748	Conrad
chr13:63059796	intergenic	none	2.0	chr1:553585-560421	CNVer
chr13:63059796	intergenic	none	2.0	chr1:554301-560300	Yoon
chr13:63059796	intergenic	none	2.0	chr1:554403-560267	Conrad
chr14:18087685	intergenic	none	2.95	chr9:69273676-69278753	CNVer
chr14:18090624	intergenic	none	5.62	chr9:69273676-69278753	CNVer
chr14:18102912	intergenic	none	15.1	chr21:13284024-13287516	CNVer
chr14:18105921	intergenic	none	2.0	chr21:13288477-13291647	CNVer
chr14:18110202	intergenic	none	8.97	chr9:69273676-69278753	CNVer
chr14:18114041	intergenic	none	6.41	chr21:13288477-13291647	CNVer
chr14:18126856	intergenic	none	3.49	chr9:67003473-67004931	CNVer
chr14:18191875	intergenic	none	9.58	chr9:66448502-66451358	CNVer
chr14:18548401	intergenic	none	2.34	chr14:18547665-18550278	CNVer
chr14:18573687	intergenic	none	3.44	chr14:19137997-19140107	CNVer
chr14:18609579	intergenic	none	2.34	chr14:19088348-19104431	CNVer
chr14:18769363	intergenic	none	4.57	chr14:18769109-18770171	CNVer
chr14:18819236	intron	LOC642669	2.78	chr9:67795537-67804278	CNVer
chr14:18846141	intergenic	none	2.17	chr9:45295377-45296746	CNVer
chr14:18877843	intergenic	none	2.82	chr14:18446674-18447927	CNVer
chr14:18892093	intergenic	none	5.2	chr14:18891569-18893565	CNVer
chr14:18984337	intergenic	none	26.55	chr14:18983822-18997002	CNVer
chr14:19139497	intergenic	none	2.94	chr14:19137997-19140107	CNVer
chr14:19152752	intergenic	none	2.14	chr14:18555165-18560919	CNVer
chr14:19167056	intergenic	none	4.37	chr14:19165798-19167632	CNVer
chr14:19179216	intergenic	none	2.4	chr14:19171146-19179499	CNVer
chr14:19242945	intergenic	none	2.7	chr14:18455004-18456974	CNVer
chr14:27388750	intergenic	none	8.42	chr9:66944283-66946395	CNVer
chr14:47927785	intergenic	none	2.0	chr11:4320231-4323794	CNVer
chr14:51291428	intergenic	none	4.09	chr11:71006200-71007715	CNVer
chr14:105164838	intergenic	none	4.0	chr14:105183371-105184609	CNVer
chr14:105648610	intergenic	none	28.8	chr16:31977525-31981507	CNVer
chr14:105659791	intergenic	none	6.67	chr16:33570084-33572189	CNVer
chr14:105666380	intergenic	none	18.0	chr16:32903288-32904526	CNVer
chr14:105691803	intergenic	none	4.5	chr16:32822500-32823976	CNVer
chr14:105703249	intergenic	none	4.0	chr16:33671680-33680640	CNVer
chr14:105829355	intergenic	none	8.9	chr16:33722458-33725406	CNVer
chr14:105942055	intergenic	none	8.9	chr16:32903288-32904526	CNVer
chr15:18270402	intergenic	none	17.0	chr21:13276329-13280385	CNVer
chr15:18279051	intergenic	none	5.1	chr21:13288477-13291647	CNVer
chr15:18367155	intergenic	none	9.46	chr7:64653493-64654919	CNVer
chr15:18369501	intergenic	none	2.19	chr16:33763306-33766812	CNVer
chr15:18376689	intergenic	none	17.88	chr7:64597627-64598678	CNVer
chr15:18408263	intergenic	none	8.54	chr16:33758958-33762607	CNVer
chr15:18522130	intergenic	none	3.0	chr15:18519412-18522967	CNVer
chr15:18577512	intergenic	none	9.79	chr2:87470066-87471179	CNVer
chr15:18578516	intergenic	none	2.81	chr2:89955932-89957203	CNVer

chr15:18632542	intergenic	none	2.0	chr15:19642865-19644797	CNVer
chr15:18754622	intergenic	none	4.07	chr16:33273657-33275215	CNVer
chr15:18758897	intergenic	none	3.66	chr16:33265033-33266295	CNVer
chr15:18767826	intergenic	none	2.91	chr15:19619196-19624831	CNVer
chr15:18767826	intergenic	none	2.5	chr2:87428172-87440514	CNVer
chr15:18775269	intergenic	none	10.0	chr9:67044163-67045630	CNVer
chr15:18775457	intergenic	none	5.15	chr15:19597380-19613724	CNVer
chr15:18802750	intergenic	none	34.93	chr2:89954336-89955548	CNVer
chr15:18988951	intergenic	none	2.75	chr15:20945989-20948032	CNVer
chr15:19533557	intergenic	none	3.0	chr15:18519412-18522967	CNVer
chr15:19588773	intergenic	none	10.46	chr2:87470066-87471179	CNVer
chr15:19643781	intergenic	none	2.0	chr15:19642865-19644797	CNVer
chr15:19809756	intergenic	none	19.5	chr14:18885447-18890644	CNVer
chr15:20202008	intergenic	none	3.0	chr16:32124384-32127152	CNVer
chr15:20274220	intergenic	none	2.45	chr15:20978675-20979684	CNVer
chr15:20947171	intergenic	none	2.09	chr15:20945989-20948032	CNVer
chr15:26137336	CDS	HERC2	25.98	chr16:32073282-32076834	CNVer
chr15:26369230	intergenic	none	3.0	chr16:32124384-32127152	CNVer
chr15:45153979	intergenic	none	33.44	chr20:25852910-25854237	CNVer
chr15:70727165	intron	LOC646670	3.87	chr15:73354138-73355897	CNVer
chr15:72168333	intergenic	none	3.87	chr15:73354138-73355897	CNVer
chr15:80517468	utr-3	LOC440295	4.03	chr15:82606345-82625912	Conrad
chr15:80727385	utr-3	LOC643696	5.35	chr15:82606345-82625912	Conrad
chr15:80903993	utr-3	LOC728636	4.06	chr15:82606345-82625912	Conrad
chr15:82625283	intergenic	none	6.47	chr15:82606345-82625912	Conrad
chr15:82629009	near-gene-3	none	2.5	chr15:82662353-82663978	CNVer
chr15:82704090	utr-3	LOC161527	5.37	chr15:82606345-82625912	Conrad
chr15:82774703	intergenic	none	4.87	chr15:82606345-82625912	Conrad
chr15:82852005	intergenic	none	4.87	chr15:82606345-82625912	Conrad
chr15:100309221	intergenic	none	2.02	chr1:27301-41300	Yoon
chr16:5118822	intergenic	none	9.5	chr8:12372363-12378948	CNVer
chr16:8554151	intergenic	none	9.0	chr16:8552624-8554150	CNVer
chr16:11937281	intron	LOC729978	3.51	chr16:68575585-68576596	CNVer
chr16:18639960	intergenic	none	11.9	chr16:16633477-16638823	CNVer
chr16:18808398	CDS	SMG1	3.08	chr16:21395241-21396698	CNVer
chr16:18908010	intron	TMC7	10.0	chr16:22415203-22420730	CNVer
chr16:21341708	intron	LOC23117	3.78	chr16:21341374-21342482	CNVer
chr16:21363615	intergenic	none	3.04	chr16:22412069-22413289	CNVer
chr16:21365078	intergenic	none	2.11	chr16:29445210-29446218	CNVer
chr16:21648010	intron	OTOA	3.0	chr16:21648369-21649569	CNVer
chr16:21654826	intron	OTOA	3.0	chr16:22465696-22472901	CNVer
chr16:21773986	intergenic	none	3.78	chr16:21341374-21342482	CNVer
chr16:21795877	intergenic	none	4.04	chr16:22412069-22413289	CNVer
chr16:21797340	intergenic	none	3.11	chr16:29445210-29446218	CNVer
chr16:22377160	intergenic	none	3.0	chr16:22376648-22377829	CNVer
chr16:22412714	intergenic	none	2.86	chr16:22412069-22413289	CNVer
chr16:22434443	intergenic	none	4.78	chr16:21341374-21342482	CNVer
chr16:22464752	intergenic	none	3.0	chr16:21648369-21649569	CNVer
chr16:28388409	intron	LOC440350	2.2	chr16:68580085-68581337	CNVer
chr16:28558582	intergenic	none	2.07	chr16:21341374-21342482	CNVer
chr16:29311014	intergenic	none	3.11	chr16:22444237-22445368	CNVer
chr16:29315636	intergenic	none	3.26	chr16:68580085-68581337	CNVer
chr16:29327701	intergenic	none	2.84	chr16:22425993-22427332	CNVer
chr16:29422213	intergenic	none	3.85	chr16:21341374-21342482	CNVer
chr16:29444190	intergenic	none	3.59	chr16:22412069-22413289	CNVer
chr16:30162182	intergenic	none	2.85	chr16:21341374-21342482	CNVer
chr16:30184174	intergenic	none	2.59	chr16:22412069-22413289	CNVer
chr16:30199109	intergenic	none	2.46	chr16:22397431-22399563	CNVer
chr16:31908935	intergenic	none	2.5	chr16:31908458-31909677	CNVer
chr16:31937283	intergenic	none	2.17	chr16:31937306-31938543	CNVer
chr16:31975031	intergenic	none	2.27	chr16:32918335-32920760	CNVer
chr16:31989145	intergenic	none	2.14	chr16:32930939-32933558	CNVer
chr16:31999436	intergenic	none	2.84	chr16:31997001-32000428	CNVer
chr16:32010957	intergenic	none	4.24	chr9:69462514-69465468	CNVer
chr16:32024661	intergenic	none	5.43	chr7:61399743-61404853	CNVer
chr16:32037894	intron	LOC647138	2.55	chr7:64653493-64654919	CNVer
chr16:32053784	intergenic	none	4.32	chr7:61367488-61373808	CNVer
chr16:32064400	intergenic	none	9.65	chr16:33742239-33743375	CNVer
chr16:32064410	intergenic	none	6.56	chr7:64204600-64205715	CNVer
chr16:32065841	intergenic	none	2.03	chr16:32960738-32963917	CNVer
chr16:32173605	intron	LOC729264	6.3	chr16:33164276-33171437	CNVer
chr16:32178512	intergenic	none	2.05	chr16:32588271-32592679	CNVer
chr16:32223088	intergenic	none	3.54	chr10:42045465-42052536	CNVer
chr16:32227861	intergenic	none	4.97	chr10:39059921-39065281	CNVer
chr16:32236256	intergenic	none	3.0	chr16:33232577-33234387	CNVer
chr16:32243971	intergenic	none	5.75	chr2:89939919-89941263	CNVer
chr16:32253250	intergenic	none	3.0	chr15:18598093-18607933	CNVer
chr16:32254866	intergenic	none	2.45	chr9:42786258-42794004	CNVer
chr16:32260111	near-gene-5	none	5.41	chr2:89923972-89928659	CNVer
chr16:32262854	intergenic	none	4.54	chr2:87428172-87440514	CNVer
chr16:32268399	intergenic	none	3.01	chr16:33265033-33266295	CNVer
chr16:32269138	intergenic	none	9.25	chr16:33225775-33228451	CNVer
chr16:32323615	intergenic	none	14.85	chr10:42066095-42067205	CNVer
chr16:32337789	intergenic	none	47.97	chr10:38990419-38991608	CNVer
chr16:32340912	intergenic	none	12.89	chr10:38987061-38988298	CNVer
chr16:32357908	intergenic	none	8.26	chr10:42094339-42096643	CNVer
chr16:32361390	intergenic	none	15.89	chr10:42097806-42099094	CNVer
chr16:32399681	intergenic	none	21.51	chr16:32027598-32032299	CNVer
chr16:32562675	intergenic	none	4.0	chr16:32978959-32987017	CNVer

chr16:32700055	intergenic	none	6.63	chr7:64204600-64205715	CNVer
chr16:32700315	intergenic	none	9.59	chr16:33742239-33743375	CNVer
chr16:32704782	intergenic	none	2.08	chr7:61464579-61475813	CNVer
chr16:32716920	intergenic	none	20.22	chr7:64597627-64598678	CNVer
chr16:32723557	intron	LOC647176	5.32	chr7:64602760-64607902	CNVer
chr16:32727284	intron	LOC647176	8.85	chr7:64653493-64654919	CNVer
chr16:32747172	intergenic	none	52.7	chr1:147089712-147090923	CNVer
chr16:32748906	intergenic	none	3.06	chr1:146929658-146949571	CNVer
chr16:32754429	intergenic	none	8.5	chr1:147097193-147101336	CNVer
chr16:32772617	intergenic	none	2.67	chr16:33712151-33718022	CNVer
chr16:32827641	intergenic	none	4.67	chr16:32827193-32828287	CNVer
chr16:32830208	intergenic	none	2.67	chr16:33650807-33652824	CNVer
chr16:32863280	intergenic	none	3.54	chr16:33617638-33619260	CNVer
chr16:32942501	intergenic	none	2.34	chr16:31997001-32000428	CNVer
chr16:32954248	intergenic	none	3.16	chr9:69462514-69465468	CNVer
chr16:32965433	intergenic	none	3.64	chr9:42718012-42721677	CNVer
chr16:32974962	intergenic	none	3.83	chr7:57928826-57935924	CNVer
chr16:32981070	intron	LOC388259	2.3	chr7:64653493-64654919	CNVer
chr16:32982193	intron	LOC388259	21.1	chr16:32038612-32039677	CNVer
chr16:32983959	intron	LOC388259	5.29	chr7:64602760-64607902	CNVer
chr16:32984263	intron	LOC388259	3.76	chr7:64649866-64652666	CNVer
chr16:32991235	intergenic	none	20.01	chr7:64597627-64598678	CNVer
chr16:33007314	intergenic	none	9.59	chr16:33742239-33743375	CNVer
chr16:33007324	intergenic	none	6.63	chr7:64204600-64205715	CNVer
chr16:33225057	intergenic	none	6.53	chr10:39059921-39065281	CNVer
chr16:33241402	intergenic	none	3.35	chr2:89939919-89941263	CNVer
chr16:33248444	intergenic	none	2.8	chr9:67044163-67045630	CNVer
chr16:33251907	intergenic	none	2.35	chr9:42786258-42794004	CNVer
chr16:33258395	intergenic	none	3.0	chr9:67032919-67040524	CNVer
chr16:33265663	intergenic	none	13.0	chr16:33265033-33266295	CNVer
chr16:33268546	intergenic	none	4.04	chr16:33268646-33270453	CNVer
chr16:33393397	intergenic	none	2.09	chr16:33392351-33398613	Conrad
chr16:33401821	intergenic	none	12.66	chr9:66706101-66707118	CNVer
chr16:33408595	intron	LOC653557	2.43	chr15:19633034-19634204	CNVer
chr16:33428861	intergenic	none	3.28	chr16:32040890-32058514	CNVer
chr16:33431628	intergenic	none	5.17	chr16:32988816-33003467	CNVer
chr16:33431628	intergenic	none	3.84	chr1:146929658-146949571	CNVer
chr16:33445506	intergenic	none	24.1	chr2:97176540-97178359	CNVer
chr16:33455106	intergenic	none	10.0	chr2:97524693-97526000	CNVer
chr16:33485137	intron	LOC401847	9.29	chr7:64602760-64607902	CNVer
chr16:33490101	intron	LOC401847	3.22	chr1:146929658-146949571	CNVer
chr16:33492794	intron	LOC401847	31.81	chr7:64597627-64598678	CNVer
chr16:33560620	intergenic	none	2.43	chr16:31977525-31981507	CNVer
chr16:33608144	intergenic	none	2.81	chr16:32873843-32874861	CNVer
chr16:33618697	intergenic	none	2.35	chr16:33617638-33619260	CNVer
chr16:33629389	intergenic	none	2.5	chr16:31908458-31909677	CNVer
chr16:33654705	intergenic	none	3.67	chr16:32827193-32828287	CNVer
chr16:33695675	intergenic	none	2.67	chr16:33691875-33702157	CNVer
chr16:33740193	intergenic	none	5.73	chr9:69459538-69461287	CNVer
chr16:33743318	intergenic	none	9.22	chr1:147089712-147090923	CNVer
chr16:33746766	intergenic	none	4.66	chr7:61063527-61065585	CNVer
chr16:33746770	intergenic	none	12.46	chr16:32022274-32023327	CNVer
chr16:33746814	intergenic	none	3.0	chr7:61399743-61404853	CNVer
chr16:33748630	intergenic	none	3.41	chr7:64684691-64685851	CNVer
chr16:33749247	intergenic	none	5.0	chr16:34032757-34035162	CNVer
chr16:33753592	intergenic	none	28.81	chr9:42723944-42727628	CNVer
chr16:33758962	intergenic	none	10.34	chr7:57928826-57935924	CNVer
chr16:33765427	intergenic	none	5.41	chr7:64602760-64607902	CNVer
chr16:34034121	intergenic	none	3.73	chr16:34032757-34035162	CNVer
chr16:45016134	intergenic	none	3.2	chr16:32968567-32977402	CNVer
chr16:45016916	intergenic	none	6.18	chr7:64211541-64213222	CNVer
chr16:45017335	intergenic	none	5.7	chr7:61399743-61404853	CNVer
chr16:45023409	intergenic	none	29.95	chr16:32950741-32951758	CNVer
chr16:45027061	intergenic	none	6.0	chr16:32968567-32977402	CNVer
chr16:45032654	intergenic	none	7.6	chr7:64687738-64692238	CNVer
chr16:45034206	intergenic	none	4.74	chr9:69454665-69458531	CNVer
chr16:45034238	intergenic	none	68.77	chr1:147089712-147090923	CNVer
chr16:45053586	intergenic	none	4.05	chr16:32960738-32963917	CNVer
chr16:68557695	intergenic	none	2.84	chr16:68557770-68559166	CNVer
chr16:68597214	intergenic	none	3.37	chr16:21355308-21356358	CNVer
chr16:68771707	intron	MGC34761	6.5	chr16:68548363-68549979	CNVer
chr16:68780579	intergenic	none	11.59	chr16:68557770-68559166	CNVer
chr16:68800833	intergenic	none	3.18	chr16:21355308-21356358	CNVer
chr16:68802662	intergenic	none	3.05	chr16:21356726-21358160	CNVer
chr16:68825422	intergenic	none	3.61	chr16:22397431-22399563	CNVer
chr16:68829895	intergenic	none	3.05	chr16:22393805-22394865	CNVer
chr16:72975363	intron	LOC440348	5.81	chr16:22444237-22445368	CNVer
chr16:72996952	intergenic	none	7.29	chr16:68557770-68559166	CNVer
chr16:73006411	intron	LOC497190	3.0	chr16:68548363-68549979	CNVer
chr16:76346354	intergenic	none	99.0	chr1:67224801-67226000	Yoon
chr16:88024361	intron	ANKRD11	2.9	chr16:88023000-88024446	CNVer
chr16:88810064	utr-3	LOC100132826	3.07	chr1:226555-227613	CNVer
chr17:253356	intergenic	none	37.9	chr17:252700-253743	CNVer
chr17:792602	CDS	”NXN,LOC100130876”	11.1	chr17:790931-792184	CNVer
chr17:1588795	near-gene-3	none	4.0	chr17:1589538-1591177	CNVer
chr17:15573073	intergenic	none	26.91	chr17:15615770-15617952	CNVer
chr17:15577829	intron	TBC1D26	2.0	chr17:18485798-18487036	CNVer
chr17:15597704	intergenic	none	10.0	chr17:18474541-18475847	CNVer
chr17:16511625	intergenic	none	19.2	chr17:15593438-15595849	CNVer

chr17:18322796	intron	LOC654346	2.4	chr17:20293419-20310020	CNVer
chr17:20294465	intron	LOC284194	2.67	chr17:20293419-20310020	CNVer
chr17:20309270	intron	LOC284194	2.8	chr17:20293419-20310020	CNVer
chr17:21272628	intergenic	none	53.9	chr17:22337007-22339323	CNVer
chr17:21285651	intergenic	none	5.67	chr17:22350645-22352583	CNVer
chr17:21289098	intergenic	none	18.91	chr17:22353951-22355269	CNVer
chr17:21831217	near-gene-5	none	2.0	chr17:21826401-21831109	CNVer
chr17:21957109	intergenic	none	4.2	chr17:22357430-22359549	CNVer
chr17:21995836	intergenic	none	7.1	chr17:20712217-20715670	CNVer
chr17:22174427	intergenic	none	4.57	chr1:121056543-121057818	CNVer
chr17:22176760	intergenic	none	24.77	chr1:121056543-121057818	CNVer
chr17:22179182	intergenic	none	5.02	chr1:121056543-121057818	CNVer
chr17:22291891	intergenic	none	17.37	chr16:33735234-33737733	CNVer
chr17:22292789	intergenic	none	4.28	chr9:69462514-69465468	CNVer
chr17:22297592	intergenic	none	25.33	chr7:64187507-64205734	Conrad
chr17:22302064	intergenic	none	24.1	chr7:61063527-61065585	CNVer
chr17:22316594	intergenic	none	14.62	chr7:64653493-64654919	CNVer
chr17:22328179	intergenic	none	2.69	chr16:32960738-32963917	CNVer
chr17:22332063	intergenic	none	8.06	chr1:147097193-147101336	CNVer
chr17:31455511	intron	CCL4	7.0	chr17:31663763-31668522	CNVer
chr17:31548566	near-gene-5	none	2.34	chr17:31547702-31550535	CNVer
chr17:33288971	intron	LOC284100	5.0	chr17:31800198-31808012	CNVer
chr17:33639386	intron	LOC440434	2.0	chr17:42990399-42991431	CNVer
chr17:40948288	intergenic	none	4.41	chr17:40952056-40953228	CNVer
chr17:40952281	intergenic	none	5.4	chr17:40952056-40953228	CNVer
chr17:57701974	intergenic	none	3.46	chr17:33417430-33420960	CNVer
chr17:57701974	intergenic	none	3.46	chr17:33361269-33364067	CNVer
chr17:57707657	intergenic	none	2.17	chr17:33550024-33552725	CNVer
chr17:57708290	intergenic	none	11.2	chr17:31882929-31883987	CNVer
chr18:11607866	intron	LOC729602	2.37	chr16:68792723-68794039	CNVer
chr18:11629458	intergenic	none	2.25	chr16:28281151-28282310	CNVer
chr18:14203236	intergenic	none	9.22	chr1:142058772-142060172	CNVer
chr18:14311536	intergenic	none	12.71	chr1:142012764-142014141	CNVer
chr18:14735039	intergenic	none	10.0	chr16:33215607-33216611	CNVer
chr18:14861505	intergenic	none	2.34	chr9:46608158-46622405	Conrad
chr18:15153992	intergenic	none	2.64	chr7:64684691-64685851	CNVer
chr18:15158207	intergenic	none	74.28	chr16:32950741-32951758	CNVer
chr18:15161503	intergenic	none	10.34	chr9:69462514-69465468	CNVer
chr18:15167538	intergenic	none	8.82	chr16:32960738-32963917	CNVer
chr18:15199392	intergenic	none	17.42	chr7:61063527-61065585	CNVer
chr18:15199823	intergenic	none	45.52	chr16:32022274-32023327	CNVer
chr18:15201231	intergenic	none	6.57	chr7:64687738-64692238	CNVer
chr18:15201622	intergenic	none	5.91	chr7:61066191-61067570	CNVer
chr18:15206524	intergenic	none	2.5	chr1:147089712-147090923	CNVer
chr18:15370514	intergenic	none	10.11	chr9:67003473-67004931	CNVer
chr18:15395391	intergenic	none	6.77	chr21:13276329-13280385	CNVer
chr18:15398440	intergenic	none	3.6	chr21:13276329-13280385	CNVer
chr18:45604808	utr-3	MYO5B	12.1	chr9:67843971-67845182	CNVer
chr19:51506	intergenic	none	2.37	chr1:47901-51700	Yoon
chr19:15646131	intron	CYP4F12	6.45	chr19:15903372-15905784	CNVer
chr19:24401175	intergenic	none	2.85	chr5:49453653-49455217	CNVer
chr19:24401175	intergenic	none	2.65	chr5:49449080-49450676	CNVer
chr19:24401175	intergenic	none	2.1	chr5:49450928-49453354	CNVer
chr19:24415480	intergenic	none	6.35	chr5:49453653-49455217	CNVer
chr19:24415480	intergenic	none	6.12	chr5:49449080-49450676	CNVer
chr19:24415480	intergenic	none	4.05	chr5:49450928-49453354	CNVer
chr19:32501446	intergenic	none	3.0	chr7:119283858-119286463	CNVer
chr19:42475745	intergenic	none	3.67	chr19:42452644-42453645	CNVer
chr19:45066590	intron	FCGBP	2.92	chr19:45066393-45067562	CNVer
chr19:45076537	CDS	FCGBP	5.92	chr19:45076592-45078743	CNVer
chr19:45082299	intron	FCGBP	2.09	chr19:45066393-45067562	CNVer
chr19:48110123	intron	PSG6	3.0	chr19:48461626-48463250	CNVer
chr19:48278820	near-gene-5	none	2.9	chr19:48464436-48466188	CNVer
chr19:48376328	intron	PSG5	7.3	chr19:48216600-48218724	CNVer
chr19:48379110	intron	PSG5	7.5	chr19:48461626-48463250	CNVer
chr19:48456784	intron	PSG9	2.9	chr19:48212364-48215112	CNVer
chr19:49606747	intergenic	none	5.78	chr4:49207006-49211872	CNVer
chr19:55293307	intergenic	none	4.67	chr19:53142491-53144742	CNVer
chr19:55296372	intergenic	none	4.67	chr19:53142491-53144742	CNVer
chr19:55299436	intergenic	none	2.85	chr19:53142491-53144742	CNVer
chr19:55330777	intergenic	none	8.85	chr19:53100179-53101298	CNVer
chr19:60029548	intron	KIR3DL1	4.0	chr19:59949644-59952473	CNVer
chr19:60034686	near-gene-3	none	2.0	chr19:59987430-59989162	CNVer
chr19:60047605	intron	KIR2DS4	2.5	chr19:59971903-59984807	CNVer
chr19:60063848	intron	KIR3DL2	8.0	chr19:59971903-59984807	CNVer
chr20:25716731	near-gene-5	none	3.65	chr20:25778776-25779855	CNVer
chr20:25778957	intergenic	none	5.96	chr20:25778776-25779855	CNVer
chr20:25805427	intergenic	none	5.77	chr7:57726342-57728045	CNVer
chr20:25860116	intergenic	none	35.07	chr20:25859292-25860770	CNVer
chr20:25875031	intergenic	none	40.51	chr20:25875365-25876407	CNVer
chr20:25880694	intergenic	none	12.53	chr20:25880315-25881485	CNVer
chr20:25883230	intergenic	none	21.0	chr20:28099038-28100281	CNVer
chr20:25884185	intergenic	none	15.17	chr20:25884211-25885333	CNVer
chr20:25995776	intergenic	none	4.65	chr20:25778776-25779855	CNVer
chr20:26066908	intergenic	none	8.0	chr17:20712217-20715670	CNVer
chr20:26203261	intergenic	none	9.91	chr7:57875926-57879690	CNVer
chr20:28082620	intergenic	none	23.34	chr20:25867003-25868543	CNVer
chr20:28091529	intergenic	none	47.93	chr20:25875365-25876407	CNVer
chr20:28099506	intergenic	none	18.92	chr20:28099038-28100281	CNVer

chr20:28100795	intergenic	none	3.9	chr20:25884211-25885333	CNVer
chr20:28110201	intergenic	none	9.54	chr20:28110161-28111358	CNVer
chr20:62416466	intergenic	none	4.22	chr1:222179551-222180909	CNVer
chr21:9756807	intergenic	none	3.0	chr9:66728130-66730631	CNVer
chr21:9777067	intergenic	none	9.5	chr21:13276329-13280385	CNVer
chr21:9780651	intergenic	none	4.25	chr21:13284024-13287516	CNVer
chr21:9912271	intergenic	none	23.91	chr7:64617298-64618615	CNVer
chr21:13280175	intergenic	none	12.25	chr21:13284024-13287516	CNVer
chr21:13287615	intergenic	none	14.72	chr21:13288477-13291647	CNVer
chr21:13445572	intergenic	none	13.9	chr7:61477981-61479977	CNVer
chr21:13447950	intergenic	none	11.1	chr16:32022274-32023327	CNVer
chr21:13644579	intergenic	none	2.53	chr9:44243216-44244387	CNVer
chr21:14199647	intergenic	none	28.64	chr1:142025320-142026647	CNVer
chr22:14446383	intergenic	none	2.67	chr9:45295377-45296746	CNVer
chr22:14462275	near-gene-3	none	3.04	chr9:69903225-69904875	CNVer
chr22:14469303	intron	LOC644525	2.07	chr9:69896618-69897954	CNVer
chr22:14657975	intron	A26C3	7.07	chr14:19071777-19080783	CNVer
chr22:14717379	intergenic	none	2.44	chr14:19137997-19140107	CNVer
chr22:14757071	intergenic	none	2.4	chr14:19171146-19179499	CNVer
chr22:14841110	intergenic	none	2.46	chr14:18885447-18890644	CNVer
chr22:15263079	intergenic	none	4.91	chr9:46608158-46622405	Conrad
chr22:15292108	intergenic	none	15.67	chr10:42088872-42089949	CNVer
chr22:15303980	intergenic	none	11.67	chr10:42081559-42083426	CNVer
chr22:15306401	intergenic	none	18.68	chr10:38990419-38991608	CNVer
chr22:15309515	intergenic	none	16.21	chr10:38993653-38994719	CNVer
chr22:15313416	intergenic	none	9.39	chr10:38997293-38998554	CNVer
chr22:15318908	intergenic	none	5.27	chr10:39002877-39003896	CNVer
chr22:15319974	intergenic	none	10.27	chr10:42066095-42067205	CNVer
chr22:15322087	intergenic	none	15.06	chr10:39005828-39006982	CNVer
chr22:15404263	intergenic	none	2.4	chr2:110113086-110114649	CNVer
chr22:15728256	intergenic	none	17.25	chr22:15401415-15402820	CNVer
chr22:15728256	intergenic	none	17.25	chr15:18594643-18595685	CNVer
chr22:15730864	intergenic	none	3.17	chr2:110113086-110114649	CNVer
chr22:15730929	intergenic	none	2.24	chr15:18588821-18592313	CNVer
chr22:15731855	intergenic	none	11.4	chr10:42030558-42031624	CNVer
chr22:17047934	intergenic	none	6.58	chr22:17117115-17118487	CNVer
chr22:17047934	intergenic	none	5.58	chr22:18721677-18722916	CNVer
chr22:17061000	intergenic	none	2.82	chr22:17252766-17253912	CNVer
chr22:17069964	intergenic	none	8.74	chr22:18703568-18705599	CNVer
chr22:17095912	intergenic	none	7.7	chr22:18703568-18705599	CNVer
chr22:17111160	intergenic	none	6.55	chr22:19998693-19999709	CNVer
chr22:17158167	intergenic	none	2.0	chr22:17155766-17165358	CNVer
chr22:17165168	intron	LOC100132900	2.0	chr22:17155766-17165358	CNVer
chr22:17187230	intron	LOC100132900	9.0	chr22:19935812-19938786	CNVer
chr22:17253002	intron	LOC653135	3.12	chr22:17252766-17253912	CNVer
chr22:17401655	intron	LOC402036	3.0	chr22:18987877-18993362	CNVer
chr22:18883590	intergenic	none	2.19	chr22:18882807-18884156	CNVer
chr22:18976441	intron	LOC729461	31.7	chr22:19998693-19999709	CNVer
chr22:19026553	intron	LOC653203	2.82	chr22:17252766-17253912	CNVer
chr22:19797726	intergenic	none	2.02	chr22:19980377-19983416	CNVer
chr22:19857812	CDS	LOC284861	4.64	chr22:17250569-17251657	CNVer
chr22:19904418	intron	GGT2	2.5	chr22:19902889-19908680	CNVer
chr22:20939175	intergenic	none	6.34	chr2:87470066-87471179	CNVer
chr22:20956954	intergenic	none	5.7	chr2:87444411-87463905	CNVer
chr22:20976139	intergenic	none	2.15	chr16:33217124-33224084	CNVer
chr22:20976467	intergenic	none	5.25	chr2:89923972-89928659	CNVer
chr22:20978489	intergenic	none	5.54	chr2:89923972-89928659	CNVer
chr22:20990279	intergenic	none	6.13	chr15:19633034-19634204	CNVer
chr22:20997357	intergenic	none	3.66	chr9:66706101-66707118	CNVer
chr22:21985186	CDS	BCR	3.67	chr22:19801471-19802780	CNVer
chr22:22001078	intergenic	none	5.87	chr22:19817056-19833887	CNVer
chr22:22001078	intergenic	none	5.87	chr22:19958604-19962441	CNVer
chr22:22147471	intergenic	none	52.32	chr22:17242480-17245812	CNVer
chr22:23337082	CDS	GGT1	3.0	chr22:17155766-17165358	CNVer
chr22:24016910	intergenic	none	17.0	chr22:24017103-24020287	CNVer
chr22:24250547	intergenic	none	42.0	chr22:23993113-23995153	CNVer
chr22:40849915	intron	LOC100132273	6.4	chr22:40856469-40864011	CNVer
chrM:1242	intergenic	none	138.9	chr5:79981609-79984011	Conrad
chrY:11706269	intergenic	none	8.63	chr9:69896618-69897954	CNVer
chrY:11746975	intergenic	none	3.69	chr1:142087471-142088768	CNVer
chrY:11748572	intergenic	none	3.17	chr1:142184499-142190108	CNVer
chrY:11797993	intergenic	none	7.48	chr1:141991684-141995184	CNVer
chrY:11828562	intergenic	none	5.69	chr1:141959533-141967507	CNVer
chrY:11892322	intergenic	none	4.48	chr3:75981170-75999076	CNVer
chrY:11894805	intergenic	none	12.59	chr3:75981170-75999076	CNVer
chrY:11969214	intergenic	none	19.86	chr10:38987061-38988298	CNVer
chrY:11973230	intergenic	none	33.28	chr10:38990419-38991608	CNVer
chrY:11975967	intergenic	none	19.2	chr10:38993653-38994719	CNVer
chrY:11982616	intergenic	none	4.47	chr10:39002877-39003896	CNVer
chrY:11983898	intergenic	none	10.1	chr10:42066095-42067205	CNVer
chrY:11985666	intergenic	none	8.71	chr10:39005828-39006982	CNVer
chrY:12052887	intergenic	none	2.5	chr9:42786258-42794004	CNVer
chrY:12052887	intergenic	none	2.5	chr9:67032919-67040524	CNVer
chrY:12052887	intergenic	none	2.5	chr2:89923972-89928659	CNVer
chrY:57378745	intergenic	none	32.14	chr2:97200363-97203926	CNVer
chrY:57402680	intergenic	none	2.0	chr18:96741-100669	CNVer

Table S4. Primer pairs for PCR amplification. Totally 19 primer pairs are designed using NCBI Primer-BLAST (http://www.ncbi.nlm.nih.gov/tools/primer-blast/) on reconstructed contig sequences. ^{a} Target region for amplification. Left=5' region of breakpoint + 5' region of duplicate. Right = 3' region of duplicate + 3' region of breakpoint. Center = 5' region of duplicate + 3' region of duplicate (to capture a partial inversion). Across = 5' region of breakpoint + 3' region of breakpoint. ^{b} Melting temperature of primers.

Region	Target^a	Pair #	Strand	Sequence	Length	Tm($^\circ$C)^b	GC%
chr8:63177986	Left	Pair 1	Forward	TGGTCCAGAAAGGGTGTGGCCTATC	25	59.94	56
			Reverse	CTCTACCCCCTCTAGAGCCCACTGT	25	60	60
		Pair 2	Forward	GAGCAGGGTGTGCATCTTTCCACAG	25	60.61	56
			Reverse	CCCCCTCTAGAGCCCACTGTAAAGC	25	60.06	60
	Right	Pair 3	Forward	ACTTGCGCTGCATGTGCCATTAAGA	25	60.01	48
			Reverse	TTGCCCTTGGTTTCATTGGAGGGTG	25	59.6	52
		Pair 4	Forward	CGTCTTGTAGACCTACTTGCGCTGC	25	59.9	56
			Reverse	TGCCCTTGGTTTCATTGGAGGGTGT	25	60.52	52
	Across	Pair 5	Forward	GAGCAGGGTGTGCATCTTTCCACAG	25	60.01	56
			Reverse	TTGCCCTTGGTTTCATTGGAGGGTG	25	59.6	52
		Pair 6	Forward	TGGTCCAGAAAGGGTGTGGCCTATC	25	59.94	56
			Reverse	TGCCCTTGGTTTCATTGGAGGGTGT	25	60.52	52
chr16:76346444	Left	Pair 7	Forward	TGAAGGCAACCTAGACACCAAACAAGT	27	58.63	44.44
			Reverse	CGGACTGCTTCCCTTCACCAATGTG	25	60.01	56
		Pair 8	Forward	TGAAGGCAACCTAGACACCAAACAAG	26	57.5	46.15
			Reverse	TCGGACTGCTTCCCTTCACCAATGT	25	60.12	52
	Right	Pair 9	Forward	TTTAGTCTCCCCGCCTCATTCCCAC	25	60	56
			Reverse	GATGTTATGGCGCACGCAAGCAATG	25	60.23	52
		Pair 10	Forward	TCCCTTCTCCCATTCCTTGTCTGCC	25	59.94	56
			Reverse	TAGAGGGGTGATGTTATGGCGCACG	25	60.62	56
	Center	Pair 11	Forward	CCATCTGCTTGCTAAAGCTACTGCCA	26	59.36	50
			Reverse	GTGGGAATGAGGCGGGGAGACTAAA	25	60	56
		Pair 12	Forward	ACCATCTGCTTGCTAAAGCTACTGCC	26	59.36	50
			Reverse	GTGCTTTGTTGGGGGTGGGAATGAG	25	60.17	56
	Across	Pair 13	Forward	TGTCTTCTCAGGGAGGAGGTTGGGA	25	60.06	56
			Reverse	TACATGGCTCAGCCCCCATGTGAAT	25	60	52
		Pair 14	Forward	TTGTCTTCTCAGGGAGGAGGTTGGG	25	59.36	56
			Reverse	AGGAAGATGGTCTCGCAGCCATGAA	25	60	52
chr5:34407364	Left	Pair 15	Forward	AGGGAGAAGATTGTCTTTCATGGTTTTTCA	30	57.18	36.67
			Reverse	AGTTTCTTCCATCACAGATGAGGTTGC	27	57.49	44.44
	Right	Pair 16	Forward	CCTCCAGCATTCCACACCCCATACT	25	60	56
			Reverse	CCTTCATGATGGGTCATGTATCCTCGTT	25	58.38	46.43
		Pair 17	Forward	GCCCTACTGCTGAGAGAGGAGCAAA	25	60	56
			Reverse	CTTCATGATGGGTCATGTATCCTCGTTT	28	57.07	42.86
chr16:18640440	Left	Pair 18	Forward	AGCTGATTCTGAGTTCACCTCCTCTC	26	57.65	50
			Reverse	GTGAGAGAACAAGACTTTGCCCCGC	25	60.23	56
	Right	Pair 19	Forward	AGTAATAGGAGATTGTGGCCAGCGCA	26	60.06	50
			Reverse	TGTGTCCCCACCCAAGTCTTATCTCA	26	58.89	50
chr12:71525450	Left	Pair 20	Forward	AACTCCCATGACACACAGTTTATCT	25	59.87	36
			Reverse	CTGAGATTTTTGTCAGCTTGGTTCT	25	60.11	40
	Right	Pair 21	Forward	ACATCAACTATCAGTGGTCATGAAT	25	59.99	36
			Reverse	GGGTTTCACCTTGGTCTCGAT	25	59.99	40
chr22:24250276	Left	Pair 22	Forward	CATCCATCCCTACTAGATCTGCATC	21	60.34	57.14
			Reverse	ATTTGTCTTTTTGTGACTGGCTGAT	24	59.73	45.83
	Right	Pair 23	Forward	CCAGTTTGAATAACTGATTAGTGGCA	21	59.23	52.38
			Reverse	TGGCAACTACCATTCTATTTCCTGT	20	58.94	55
chr6:24791647	Left	Pair 24	Forward	ATGTGAACCCCAAAAATTTGAGACA	24	57.65	50
			Reverse	ACTCATTTGTTTATTCACTGAGCCG	20	60.23	56
	Right	Pair 25	Forward	AGTGAATAAACAAATGAGTGCGTGT	24	60.06	50
			Reverse	TACAAGGAAAATTCAAGCTGGGAAC	24	58.89	50
chr14:105164829	Left	Pair 26	Forward	CCTGACCTCTGAGGAGGCTAA	21	60.34	57.14
			Reverse	GCATCACTCAGCTCCTAGATTTTG	24	59.73	45.83
	Right	Pair 27	Forward	GCTCTGATTCCCTGAGGCTAA	21	59.23	52.38
			Reverse	GGAACTCAGGAAACAGGGGA	20	58.94	55
chr7:61530853	Left	Pair 28	Forward	TCCATTCTAGGAAAGGGCATTTGT	24	60.26	41.67
			Reverse	TGCACACATCCCAAACGTGG	20	61.45	55
	Right	Pair 29	Forward	GTGAAGCTGTATTTGGGATTGCAT	24	60.14	41.67
			Reverse	AGTTTCTCAGAGAGCCTCACTCTA	24	60.26	45.83
chrY:57378673	Left	Pair 30	Forward	CTCTCTCCCATAGACACTTTCACAA	25	60.05	44
			Reverse	TAAAAGGCTGTGTCTACCAAAGAGT	25	60.16	40
	Right	Pair 31	Forward	TCTCTGGAAAGCATAGACATAGTGG	25	59.93	44
			Reverse	TCCCCTTAAAGCATGTCTTTTTGAC	25	59.99	40

3 Supplementary Methods

3.1 Parameter initialization for profile Hidden Markov Model

A profile HMM is described by the tuple (Q, V, π, A, B) referring to States, Symbols, Initial state distribution, Transition and Emission probabilities, respectively. Q=$\{q_1, q_2, ..., q_n\}$ is the set of states, V=$\{v_1, v_2, ..., v_m\}$ is the output alphabet, $\pi(i)$ is the probability of being in state q_i at time $t = 0$(initial state), A=$\{a(q_i, q_j)|1 \leq i, j \leq n\}$ is the set of transition probabilities, where $a_(q_i, q_j)$ is the probability of entering state q_j at time $t + 1$ from state q_i at time t, and B=$\{b(q_j, q_k)\}$ is a set of output probabilities, where $b(q_j, v_k)$ is the probability of producing v_k at time t when in state q_j at time t. Here, each state q_i corresponds to a single base pair of a duplicate D, and the output alphabets consist of a single nucleotide ('A', 'T', 'G', and 'C') including any probabilistic combinations (e.g. 'M'='A' or 'C', 'R'='A' or 'G', and 'N'=any nucleotide), and an empty character (*null*).

To initialize background emission probabilities (b_0), we calculate a number of observation (c_k) of each alphabet v_k in the template sequence (H).

$$b_0(v_k) = \frac{c_k}{\sum_k c_k} \tag{1}$$

Background transition probabilities can be initialized by assigning equal probabilities to the all possible transitions of the current state.

$$a(M_i, M_{i+1}) = a(M_i, D_{i+1}) = a(M_i, I_i) = \frac{1}{3} \tag{2}$$

$$a(I_i, I_i) = a(I_i, M_{i+1}) = \frac{1}{2} \tag{3}$$

$$a(D_i, M_{i+1}) = a(D_i, D_{i+1}) = \frac{1}{2} \tag{4}$$

3.2 Parameter training for profile Hidden Markov Model

Training a PHMM includes updating emission and transition probabilities at the site of new observations. Assume that we observed O_k time of an alphabet v_k at a given match state. The total number of observation O is now $\sum O_k$. The updated emission probability $b(v_k)$ after O times of observation should satisfy following constraints.

$$b(v_k) \propto \frac{O_k}{O} \tag{5}$$

$$\lim_{O \to \infty, O_k = 0} b(v_k) \geq \mu, \lim_{O \to \infty, O_k = O} b(v_k) \leq 1 - \mu \tag{6}$$

$$\sum_k b(v_k) = 1 \tag{7}$$

Expression 5 describes that the emission probability should be proportional to the observation frequency (pseudocount). The Inequality 6 limits the range of $b(v_k)$ by applying a minimum and a maximum value μ, which corresponds to the known sequencing error rate. Because the output is not a direct observation, there always is a chance to observe difference alphabets even though a state is perfectly trained to emit a single output. More importantly, if an emission probability of 0 (or 1) is allowed for each match state, we would not be able to variate the PHMM model because any sequences with a single variation will not be assigned to the model. Considering the constrains, we can set up the first emission function:

$$b(v_k) = \frac{xO_k + yc_k}{zO + wc} + \mu, \tag{8}$$

where x,y,z, and w are constants for controlling weights. We can apply the initial emission probability in Equation 1 given no observations ($O = \sum O_k = 0$) to Equation 8.

$$b_0(v_k) = \frac{yc_k}{wc} + \mu = \frac{c_k}{c} \tag{9}$$

By applying $y = w - wc\mu/c_k$ to Equation 8, we get:

$$b(v_k) = \frac{xO_k + w(c_k - \mu C)}{yO + wc} + \mu \tag{10}$$

And from Expression 7, we get:

$$\sum_k b(v_k) = \sum_k (\frac{xO_k + w(c_k - \mu C)}{yO + wc} + \mu) = \frac{xO + wc - wmc\mu}{yO + wc} + m\mu = 1, \tag{11}$$

where m is the total number of alphabets. By applying $x = (1 - m\mu)y$ from Equation 11 to Equation 10, we get:

$$b(v_k) = \frac{(1 - m\mu)yO_k + w(c_k - c\mu)}{yO + wc} + \mu \tag{12}$$

If we rewrite the equation using $\rho = y/w$, we finally get:

$$b(v_k) = \frac{(1 - m\mu)\rho O_k + (c_k - c\mu)}{\rho O + c} + \mu \tag{13}$$

Here, the only parameter is ρ, which is used to control weight between the effect from observation (O_k) and the background information (c_k). The bigger ρ provides faster training from same number of observations, and make the probability converge to its limit value ($1 - \mu$) more rapidly. Training transition probabilities $a(q_i, q_j)$ can be defined in a similar way particularly by regarding the observations as the number of observed transitions instead of observed outputs.

3.3 Training transition probabilities

To update transition probabilities, we use observations of transitions $a(q_i, q_j)$ for training where the range of q_j is differently defined depending on the type of q_i. For example, if q_i is a match state M_i, the set of possible transition A_i is $\{M_{i+1}, I_i, D_{i+1}\}$ (see Figure S11). So the transition probability after observation can be calculated from Equation **??** when we substitute $b(v_k)$, O_k and O to $a(q_i, q_j)$, O_{ij} and O_i respectively:

$$O_{ij} = \text{\# of observation } a(q_i, q_j) \tag{14}$$

$$O_i = \sum_{j \in A_i} O_{ij}. \tag{15}$$

3.4 Prediction of breakpoint zygosity

Consider a candidate breakpoint region, where a duplicon is inserted in the donor and not the reference. For a homozygous insertion, we do not expect to see any concordant read straddling the breakpoint. For a heterozygous insertion, we expect the coverage to reduce by half (Figure S8).

To check zygosity, we model the clone depth distribution around breakpoints. Define λ as an expected number of clones that start at a specific location. The expected coverage at location x, $C(x)$, can be defined by the expected number of clones that start between $x - l$ and x, where l is the clone length (insert size):

$$C(x) = \lambda l. \tag{16}$$

Assuming that the clone depth follows a Poisson distribution, the probability that we observe k clones spanning position x without any insertion breakpoints is:

$$P(k \text{ clones span } x) = f(k; \lambda l) = \frac{C(x)^k e^{-C(x)}}{k!} \tag{17}$$

$$= \frac{(\lambda l)^k e^{-\lambda l}}{k!}. \tag{18}$$

Likewise, the probability that we observe k clones at a position i bp ($i < l$) away from the breakpoint b is:

$$f(k; \lambda i) = \frac{(\lambda i)^k e^{-\lambda i}}{k!}, \tag{19}$$

because the clone start point must be located in $[b - i - l, b - l]$ allowing only i bp range instead of l. The probability of observing data at $\pm l$ bp around the breakpoint given a zygosity z can be calculated as below:

$$P(\text{obs}|z) = \prod_{i=b-l}^{b+l} f(k_i; \lambda i) \tag{20}$$

$$= \begin{cases} \prod_i \frac{(\lambda l)^{k_i} e^{-\lambda l}}{k_i!} & z = \text{normal} \\ \prod_i \frac{(\lambda(i+l)/2)^{k_i} e^{-\lambda(i+l)/2}}{k_i!} & z = \text{heterozygous} \\ \prod_i \frac{(\lambda i)^{k_i} e^{-\lambda i}}{k_i!} & z = \text{homozygous.} \end{cases} \tag{21}$$

So the probability of zygosity z of breakpoint given observation can be calculated from priors:

$$P(z|\text{obs}) = \frac{P(\text{obs}|z)P(z)}{\sum P(\text{obs}|z)P(z)}. \tag{22}$$

$$\tag{23}$$

Finally, REPREVER$_{\text{LOC}}$ calls the zygosity of maximal likelihood.

3.5 PCR amplification of duplicated regions:

Totally 19 primer pairs are designed for PCR amplification of the four test cases (Table S4) using NCBI Primer-BLAST (`http://www.ncbi.nlm.nih.gov/tools/primer-blast/`). Consensus sequences reconstructed by REPREVER$_{\text{SEQ}}$ were used as templates. For each consensus template, primer sequences are selected by following criteria: 1) primer length is 25 to 30 bp. 2) product size is 300 to 700 bp. 3) melting temperature is 57 to 63°C. 4) 50 bp breakpoint flanking regions are excluded. For regions in known segmental duplication, least similar sequences are used to build primers yielding also limited primer quality.

PCR reactions were performed on 100ng of NA18507 genomic DNA template in a 40ul PCR reaction consisting of 2ul (100ng) each primer, 4ul (25mM) dNTPs, 4ul (1X) PCR buffer, 1ul DMSO, 0.4ul (1.2 U) PicoMaxx High-Fidelity Polymerase (Agilent Technologies) and 25.3ul water. Cycling conditions for the PCR reaction were as follows: 1 cycle of 94°C for 5 minutes followed by 30 cycles of 94°C for 30 seconds, 62°C for 30 seconds

and 72°C for 1 minute and finally 1 cycle of 94°C for 7 minutes and 10°C forever. 2ul of PCR reactions were run on a 1.5agarose gel for visualization. Where single PCR bands were observed, products were purified using the MinElute PCR Purification Kit (Qiagen) using the manufacturer's recommended protocol. If gel purification was needed, the product was extracted from the gel and purified using the QIAquick Gel Extraction Kit (Qiagen).

3.6 Sanger sequencing of duplicons:

For each successfully amplified region, 50ng of purified PCR product (prepared as described above) mixed with 50ng of forward primer were submitted to The Centre for Applied Genomics' Sanger sequencing facility (Toronto, Ontario, Canada; http://www.tcag.ca/facilities/ dnaSequencingSynthesis.html). Sequencing reactions were performed using Big Dye Terminator v3.1 cycle sequencing enzyme (Life Technologies) and run on a 96-capillary 3730xl DNA Analyzer (Applied Biosystems/Life Technologies).

3.7 Complete information of data preparation and processing

3.7.1 Simulated data and test:

One thousand artificial reference 'genomes' **R** were constructed as follows: each genome had five, 50 kbp 'chromosomes', selected at random from chr1 of hg18, and satisfying the following properties. Each region had less than 10% non-ACGT characters, and at most 50% of the region marked as being repetitive (marked by RepeatMasker Jurka et al. (2005), simpleRepeats, microsatellite Benson (1999), or segmental duplications Bailey et al. (2001)). To create artificial donor genomes $\mathbf{D}$ including duplicons, we first introduce duplicons in R and mutate the entire region. For duplicons, a random 1-3 kbp region H (less than 20% repetitive) was selected, and for duplicating into 1-4 copies. Note that there are no limits in the number of duplicons to be reconstructed by REPREVER$_{\text{SEQ}}$, but duplicons up to four copies are used in the simulation for computational convenience ($l < 5$). Each copy was truncated, and mutated to form a duplicon. Mutations were introduced using a mismatch rate of 0-4%, a gap-open 0-0.5%, and probability of a gap-extension 0-33%. Finally, the duplicons were inserted at randomly chosen chromosomes and starting positions at least 1 kbp from the original copy (non-tandem duplications). Next, the donor genome is mutated away from **R** using rates 0.107%, 0.014% for substitution, and indels, respectively (parameters chosen from Levy Levy et al. (2007)). Finally, 50k ($100x2$ bp, 500 ± 20 bp insert) paired-end reads were simulated using *Maq-simulate* Li et al. (2008). Sequencing error was emulated by building 'simupar.dat' from a part of conventional Illumina paired end sequence data (SRR034939). The generated reads were mapped to **R** with 'bwa samse' and converted into BAM format using SAMtools Li et al. (2009).

To evaluate accuracy in simulated data, we compared each *inferred* duplicon sequences I_i against the true (but unknown) donor duplicons A_i using Blast (blastn ver. 2.2.25) to compute $\%$-identity, aligned-lengths and bit-scores. The accuracy of breakpoint location was also calculated from positional difference between the true and inferred breakpoints. Finally, we classified each instance according to the similarity between the template sequence A_H and A_i to investigate performance as a function of divergence.

3.7.2 Sequencing data:

Paired end sequencing (SRX016231, Illumina 100x2 base and $\sim$ 500 bp insert, 40x) of Yoruban individual (NA18507) was downloaded from NCBI's Sequence Read Archive website (`http://www.ncbi.nlm.nih.gov/sra?term=SRX016231`). Human genome reference assembly (NCBI36/hg18) was downloaded from UCSC Genome Browser. To map the sequencing data to the reference genome, a paired end mapping version of BWA (bwa *aln* followed by bwa *sampe*) was used to reduce the number of false discordant mate-pair calls (Li and Durbin, 2009). A slightly loosen parameter for maximum edit distance (6%) was used to increase the initial

coverage. All other parameters were set to default. Another whole genome sequencing dataset (SRR034939, 150x2 base and $\sim$ 350 bp insert, 20x) of European individual (NA12878) was downloaded from SRA and processed similarly. After getting the whole genome mapping file (.bam), we used SAMtools to convert formats for tools in REPREVER. For additional processing, SAM-JDK library (ver. 1.40) was downloaded from Picard (`http://picard.sourceforge.net`) and imported to REPREVER toolkit.

3.7.3 Candidate copy number increased regions:

Three independent CNV callsets were used as REPREVER input. First, a CNV list of 500 regions was downloaded from the online version of the study Yoon et al. (2009). We selected 100 regions marked with "duplication" out of 500, and further reduced to 85 by filtering out short ($<$ 1 kb) duplications. Second, a list of 206 regions that are predicted to have absolute copy number bigger than 2 (CN $>$ 2) in NA18507 genome was downloaded from the project website (`http://www.sanger.ac.uk/research/areas/humangenetics/cnv/highres\_discovery.html`) Conrad et al. (2010). A third dataset was generated by running CNVer v0.8.1 Medvedev et al. (2010) on the NA18507 sequencing dataset. As required by CNVer, we first re-mapped the reads using bowtie to report all good alignments using the suggested parameters. We ran CNVer with parameter min_mps=5, thus requiring a cluster to contain at least five supporting mappings. Out of initial 5325 CNVs we selected 2022 copy number increased regions (relative donor copy change$>$0) and further reduced to 1876 long ($>$ 1 kb) regions.

3.7.4 Fosmid clone sequences and validated insertions

A dataset of 226 fully sequenced fosmids from NA18507 was downloaded from NCBI GenBank via Human Genome Structural Variation Project homepage (`http://hgsv.washington.edu/`). We conducted the following procedures to discover insertion sequences that are contained in the fosmid set. 1) We ran Blat for each fosmid sequence against the hg18 reference genome to determine mapping sites. The best match is selected using Blat match-score constrained by the fosmid's origins. 2) For each best-match, we compared the size of region used in query (ℓ_f) and match (ℓ_r). We classified the fosmid match into 4 classes: i) normal, where $|\ell_f$-$\ell_r| < 1000$, ii) deletion, where ℓ_r-$\ell_f > 1000$, iii) insertion, where ℓ_f-$\ell_r > 1000$ and iv) others (e.g. no preferential matches or split-match). From this step, we found 16 fosmids that have potential insertion sequences (79 normal, 61 deletion, and 70 others). 3) We conducted pairwise alignment between the matched fosmid and the corresponding reference sequences using Strecher Rice et al. (2000) to confirm the exact insertion location and sequence. 4) The inserted sequence is queried against hg18 reference using Blat to check i) novelty and ii) type of the sequence (e.g. repeat element). We found 12/16 do not exist in the reference, confirming they are not duplicated insertions; all of the 4 previously validated insertions from a subset of the 226 fosmids Kidd et al. (2008) were discovered here again. Out of 4 remaining duplicated sequences, 2 could not be determined due to mapping ambiguity (e.g. microsatellite), leaving 2 as working examples.

Another dataset of 454 fully sequenced fosmids from NA12878 was downloaded and processed similarly to discover 74 insertions. Out of 74, we found 7 were unambiguously duplicated (61 novel, 6 undetermined).

3.8 Comparison with split-read based algorithms

There are more tools for detecting structural variations. Pindel (Ye *et al* 2009) and PRISM (Yue *et al* 2012) are two well known tools based on split-read algorithm. We tested these tools on calling non-tandem duplications. Pindel targets (a) large deletions (1bp$\sim$10kb) and (b) medium sized insertions (1$\sim$20bp). More recently (2011), the same authors announced that the Pindel has been improved to target more SV types (`https://trac.nbic.nl/pindel/wiki/BackgroundInformation`), which includes (c) tandem duplications,

Table S5: Comparison result with split-based tools (Pindel and PRISM).

SV type	Pindel	PRISM
Deletions	41984	
Insertions (short)	310202	784319
Insertions (large)	944	
Tandem duplications	3613	407
Inversions	469	172
PCR validated interspersed duplications	1/9	0/9

(d) inversions and (e) large insertions. Please note that read signatures of tandem duplication are completely different from those of interspersed duplication; algorithms for one type cannot be used for detecting the other type. PRISM targets arbitrary-sized (a) inversions, (b) deletions and (c) tandem duplication; PRISM_CTX, an independent tool from PRISM, targets inter-chromosome translocation events. DELLY (Tobias et al, 2012) targets (a) deletions, (b) tandem duplications, (c) inversions, and (d) translocations; note that translocation is also far from interspersed duplication where it does not accompany homologous regions and copy number changes

We compared Pindel and PRISM on the same NA18507 dataset. As shown the Table below, both tools did not report any breakpoints explicitly marked "interspersed duplication". In Pindel, only one PCR validated breakpoint "chr1:63177985" was called as a large insertion. PRISM did the same study in its paper to detect 784319 indels, 172 inversions and 407 tandem duplications. However insertion sizes were only constrained to less than 100 bp reporting no interspersed duplications. Therefore, there is no other current tool that is specialized in calling duplicons targeted in our study.

4 References

References

Bailey, J. A., Yavor, A. M., Massa, H. F., Trask, B. J., and Eichler, E. E., 2001. Segmental duplications: organization and impact within the current human genome project assembly. *Genome Res.*, **11**:1005–1017.

Benson, G., 1999. Tandem repeats finder: a program to analyze DNA sequences. *Nucleic Acids Res.*, **27**:573–580.

Bentley, D. R., Balasubramanian, S., Swerdlow, H. P., Smith, G. P., Milton, J., Brown, C. G., Hall, K. P., Evers, D. J., Barnes, C. L., Bignell, H. R., *et al.*, 2008. Accurate whole human genome sequencing using reversible terminator chemistry. *Nature*, **456**:53–59.

Conrad, D. F., Pinto, D., Redon, R., Feuk, L., Gokcumen, O., Zhang, Y., Aerts, J., Andrews, T. D., Barnes, C., Campbell, P., *et al.*, 2010. Origins and functional impact of copy number variation in the human genome. *Nature*, **464**:704–712.

Hajirasouliha, I., Hormozdiari, F., Alkan, C., Kidd, J. M., Birol, I., Eichler, E. E., and Sahinalp, S. C., 2010. Detection and characterization of novel sequence insertions using paired-end next-generation sequencing. *Bioinformatics*, **26**:1277–1283.

Jurka, J., Kapitonov, V. V., Pavlicek, A., Klonowski, P., Kohany, O., and Walichiewicz, J., 2005. Repbase Update, a database of eukaryotic repetitive elements. *Cytogenet. Genome Res.*, **110**:462–467.

Kidd, J. M., Cooper, G. M., Donahue, W. F., Hayden, H. S., Sampas, N., Graves, T., Hansen, N., Teague, B., Alkan, C., Antonacci, F., *et al.*, 2008. Mapping and sequencing of structural variation from eight human genomes. *Nature*, **453**:56–64.

Krzywinski, M., Schein, J., Birol, I., Connors, J., Gascoyne, R., Horsman, D., Jones, S. J., and Marra, M. A., 2009. Circos: an information aesthetic for comparative genomics. *Genome Res.*, **19**:1639–1645.

Levy, S., Sutton, G., Ng, P. C., Feuk, L., Halpern, A. L., Walenz, B. P., Axelrod, N., Huang, J., Kirkness, E. F., Denisov, G., *et al.*, 2007. The diploid genome sequence of an individual human. *PLoS Biol.*, **5**:e254.

Li, H., Handsaker, B., Wysoker, A., Fennell, T., Ruan, J., Homer, N., Marth, G., Abecasis, G., and Durbin, R., 2009. The Sequence Alignment/Map format and SAMtools. *Bioinformatics*, **25**(16):2078–2079.

Li, H., Ruan, J., and Durbin, R., 2008. Mapping short DNA sequencing reads and calling variants using mapping quality scores. *Genome Res.*, **18**:1851–1858.

Li, Y., Zheng, H., Luo, R., Wu, H., Zhu, H., Li, R., Cao, H., Wu, B., Huang, S., Shao, H., *et al.*, 2011. Structural variation in two human genomes mapped at single-nucleotide resolution by whole genome de novo assembly. *Nat. Biotechnol.*, **29**(8):723–730.

McKernan, K. J., Peckham, H. E., Costa, G. L., McLaughlin, S. F., Fu, Y., Tsung, E. F., Clouser, C. R., Duncan, C., Ichikawa, J. K., Lee, C. C., *et al.*, 2009. Sequence and structural variation in a human genome uncovered by short-read, massively parallel ligation sequencing using two-base encoding. *Genome Res.*, **19**:1527–1541.

Medvedev, P., Fiume, M., Dzamba, M., Smith, T., and Brudno, M., 2010. Detecting copy number variation with mated short reads. *Genome Res.*, **20**:1613–1622.

Rice, P., Longden, I., and Bleasby, A., 2000. EMBOSS: the European Molecular Biology Open Software Suite. *Trends Genet.*, **16**(6):276–277.

Ye, K., Schulz, M. H., Long, Q., Apweiler, R., and Ning, Z., 2009. Pindel: a pattern growth approach to detect break points of large deletions and medium sized insertions from paired-end short reads. *Bioinformatics*, **25**(21):2865–2871.

Yoon, S., Xuan, Z., Makarov, V., Ye, K., and Sebat, J., 2009. Sensitive and accurate detection of copy number variants using read depth of coverage. *Genome Res.*, **19**(9):1586–1592.
